# The Effect of Copper on the Color of Shrimps: Redder Is Not Always Healthier

**DOI:** 10.1371/journal.pone.0107673

**Published:** 2014-09-17

**Authors:** Ana Martínez, Yanet Romero, Tania Castillo, Maite Mascaró, Isabel López-Rull, Nuno Simões, Flor Arcega-Cabrera, Gabriela Gaxiola, Andrés Barbosa

**Affiliations:** 1 Instituto de Investigaciones en Materiales, Universidad Nacional Autónoma de México, México DF, México; 2 Unidad Multidisciplinaria de Docencia e Investigación, Sisal, Universidad Nacional Autónoma de México, Mérida, Yucatán, México; 3 Centro Tlaxcala de Biología de la Conducta, Universidad Autónoma de Tlaxcala, Tlaxcala, Tlaxcala, México; 4 Unidad de Química Sisal, Facultad de Química, Universidad Nacional Autónoma de México, Mérida, Yucatán, México; 5 Departamento de Ecología Evolutiva, Museo Nacional de Ciencias Naturales, CSIC, Madrid, Spain; Arizona State University, United States of America

## Abstract

The objective of this research is to test the effects of copper on the color of pacific white shrimp (*Litopenaeus vannamei*) *in* *vivo*. Forty-eight shrimps (*L. vannamei*) were exposed to a low concentration of copper (1 mg/L; experimental treatment) and forty-eight shrimps were used as controls (no copper added to the water). As a result of this experiment, it was found that shrimps with more copper are significantly redder than those designated as controls (hue _(500–700 nm)_: P = 0.0015; red chroma _(625–700 nm)_: P<0.0001). These results indicate that redder color may result from exposure to copper and challenge the commonly held view that highly pigmented shrimps are healthier than pale shrimps.

## Introduction

Yellow, orange and red pigmentation, manifest in animals and plants, is mostly caused by carotenoids [1]–[4]. These colorful substances are extensively present in nature and are considered useful antioxidants or antiradicals, preventing diseases caused by oxidative stress [5]–[8]. In particular, the intensity of red pink coloration of crustaceans (as shrimps) is controlled by the concentration of astaxanthin [9], a natural carotenoid that also acts as an effective antioxidant [6]–[8]. Crustaceans do not synthesize carotenoids *de novo*. Many crustaceans can synthesize astaxanthin from precursors as ß-carotene ingested from dietary, and as a consequence, they accumulate carotenoids from food [9]. Therefore, high astaxanthin content indicates the availability of a suitable diet during the growth and development of crustaceans, which is why the color of shrimps is regarded as an indication of health, thus affecting its commercial value [10]–[14]. More intense color suggests better taste and improved food quality [11]. Consequently, an important effort is made in the food industry to intensify the color of products [12]–[13]. Among crustaceans, an example can be found in Pacific white shrimp (*Penaeus vannamei),* in which a carotenoid supplementation using Aztec marigold “cempasúchil” (*Tagetes erecta)* results in redder individuals [13]. Furthermore, there is a variation in price conforming to the redness of these animals [14], so that the more intense the red color, the higher the price. Color of shrimps is so important that several studies have been devoted to the effect of light radiation on the color of organisms [15]–[17], as well as to the relationship between body color, carotenoid concentration and dietary supplementation [18], [19].

Heavy metals such as copper have an impact on the metabolism of aquatic species [20]–[26]. Experiments evaluating the toxicity of copper in shrimps indicate that high concentrations of copper particularly affect osmoregulation, molting frequency and survival [20]–[23]. Recent results from computational chemistry [27] indicate that the presence of metals such as copper, lead, mercury and cadmium, combined with astaxanthin causes the formation of novel complexes that are redder (larger lambda maxima) in appearance. The two oxygen atoms on the terminal cyclohexene ring of astaxanthin chelate the metal and form these complexes, as described in experiments by Polyakov et al. [28], who likewise assess the effect of metal on coloration. These results may have important implications, as metal pollutants are commonly present in aquatic ecosystems [24], [29]. Whilst theoretical and experimental results exist, testifying to the modification of astaxanthin in the presence of metal atoms, it is necessary to corroborate that this modification actually occurs among live animals, altering their natural coloration. Despite ample studies, describing the effects of heavy metals on aquatic organisms together with studies that focus on the body color of shrimps, the effect of exposure to heavy metals on the body pigmentation of shrimps remains to be investigated. Therefore the objective of this research is to test *in* *vivo,* the effects of copper on the color of the pacific white shrimp (*Litopenaeus vannamei*).

## Materials and Methods

Seawater was extracted from an unprotected marine area, where the laboratory facilities are located (21°09′N 90°01′W). In conformity with Mexican federal and state laws, as this is neither a protected area, nor private land, we did not require any type of permission. Our study did not involve endangered or protected species.

Two independent closed circuit water circulation systems, each with 6 glass aquaria (39×33×19 cm D×W×H; ≈ 25L) and a reservoir; 47 L) were prepared. Seawater was extracted directly from the sea and filtered (sand filter 20 and 30 microns and then subjected to a bag filter 25, 10 and 5 microns). Water was maintained at 25±1°C throughout the entire experiment. 12×12 light/dark period was controlled with white light lamps. Copper was added to one of the independent closed circuits (1 mg/L; experimental treatment) as CuSO_4_•5H_2_O (Sigma Aldrich Technical Grade), one day prior to the initiation of the experiment. This concentration of copper far exceeds that found naturally in the ocean (approximately 0.00025 mg/L, [30]), but is much lower than the median lethal concentration (96 h LC50; 37.3 mg/L [29]), thus ensuring shrimp survival and making it possible to analyze the effect of low concentration of copper on the color of shrimps.

Shrimps pertained to the *Litopenaeus vannamei* species, which is neither endangered, nor under legal protection. They were grown in floc in outdoor tanks. Floc contains microalgae, nematodes and some copepod bloom [31] and is a source of carotenoids [32]. Ninety-six shrimps (2.5–8.5 g wet body weight) were taken from these outdoors tanks and randomly and evenly assigned to one of the independent closed circuit water circulation systems, described previously in this section. Each glass aquarium contained 8 shrimps. During the experiment, shrimps were fed with pellets containing vitamins but not carotenoids. This means that their only source of carotenoids was derived from floc during the growing process.

Forty-eight shrimps (*L. vannamei*) were exposed to copper at a concentration of 1 mg/L (experimental), whilst forty-eight shrimps were maintained in clean seawater (controls). All shrimps were similar in size (6.95–10.32 cm) and were at the intermolt stage. Two experimental shrimps died during the experiment and were excluded from the analyses. Color was assessed at two stages: after 4 and 9 days under experimental conditions. On day four, 4 shrimps were collected from each aquarium to assess color, so that 4 shrimps remained in each glass aquarium. Those remaining were collected on day 9 (end of the experiment). Shrimps were sacrificed (submerged in boiling distilled water (100°C) for one minute immediately after collection), in order to prevent possible *post mortem* changes in color.

The most objective and reliable method for assessing color is spectrophotometry, which measures the distribution of wavelengths reflected, via a digital device known as a spectrophotometer. Spectrophotometry can also measure color, other than that which is visible to humans (e.g. ultraviolet and infrared wavelengths), and is therefore useful in the case of animals, as it may be that they perceive colors beyond the spectrum visible to humans [33], [34]. Reflectance spectra were obtained from 300 to 800 nm using a spectrophotometer (Ocean Optics USB2000). The diameter of the illuminated region was 6 mm and measurements were taken at a 45° angle, using an attachment designed for the purpose.

In this investigation, color was assessed in the head region immediately after sacrifice, using the spectrophotometer. Using the reflectance spectra, we calculated three colorimetric variables [35]: hue (the wavelength at maximum reflectance); red chroma saturation (the proportion of total reflectance in the red region; *i.e.* the proportion of light reflected at the wavelengths of red chroma); and light (the total amount of light reflected). Shrimp color was measured in the head area just below the eye three times over, and the arithmetic mean of these measurements was used for subsequent analysis. Red shrimp coloration was tested using a General Linear Mixed Model (GLMM), where hue, red chroma and total light were the dependent variables; tank was included as a random factor, whereas time (4 or 9 days of exposure) and treatment were applied as fixed factors, and weight and body size as covariate. The copper concentration in shrimps was measured using the atomic absorption technique. Shrimp heads were separated from their bodies. Heads were freeze dried in a Labconco FreeZone 2.5 freeze dryer. Dried shrimp heads were weighed and placed in Teflon vessels containing 9 mL of HNO_3_ (JT Baker ACS) for total digestion, in a microwave digestion system. Total copper was determined by absorption spectrometry, applying the acetylene flame technique [36].

## Results and Discussion

**Figure 1:**
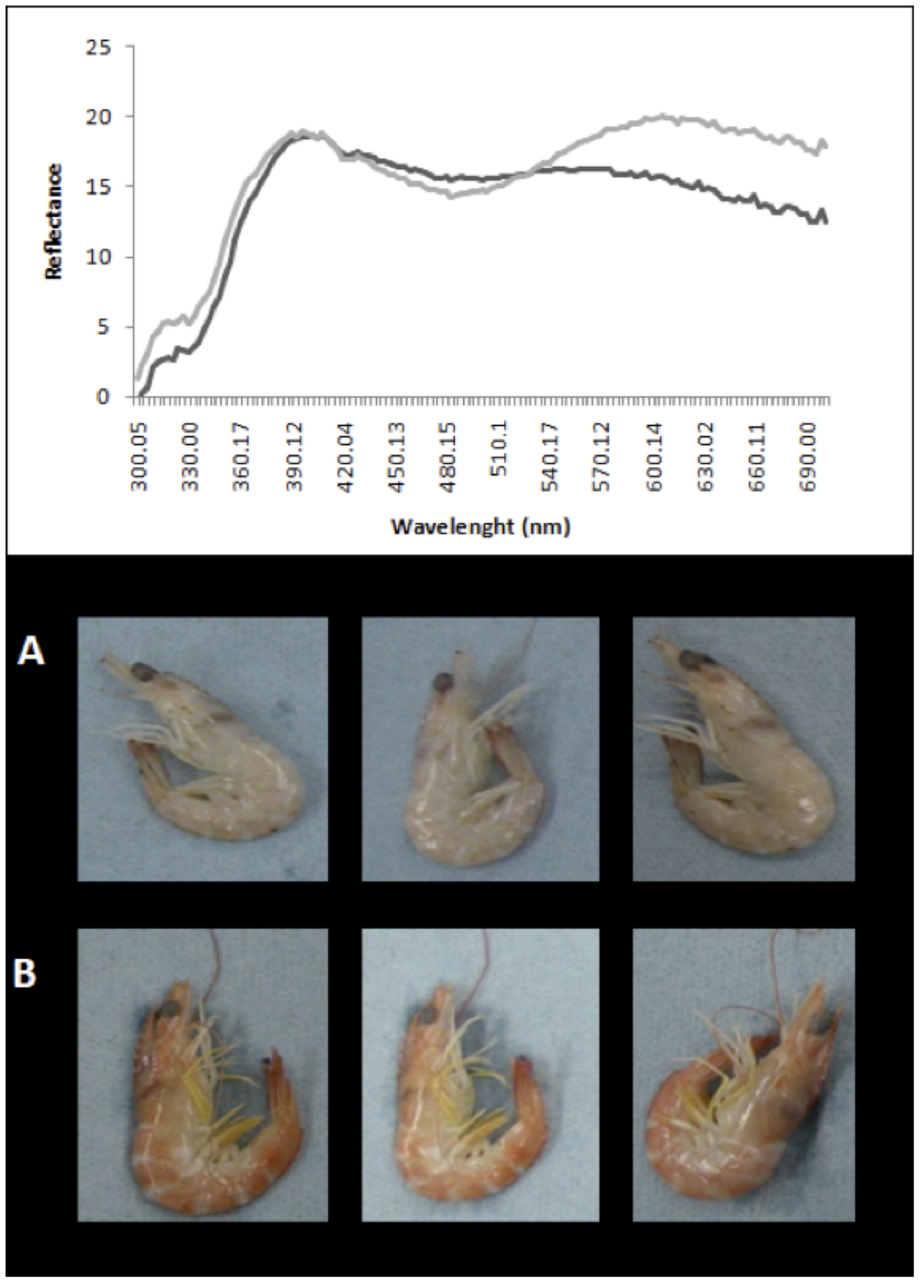
Color of shrimps. Reflectance spectra for head coloration in control (black line) and experimental (grey line) shrimps. Visual aspects of selected shrimps not exposed (A) and exposed (B) to 1 mg/L of Cu during 9 days are shown in the image, in order to highlight the effect.

Results related to copper concentration indicated that it was higher among experimental shrimps than among controls (ANOVA F_1,89_ = 16.735, P = 0.00009; control 172.32±13.41 mg/Kg and experimental 250.31±13.55 mg/Kg). Figure 1 presents the reflectance spectra of head coloration, in control (black line) and experimental (grey line) shrimps. Reflectance with wavelengths that fall between 625 and 700 nm (red chroma) was greater in the case of experimental shrimps (grey line). This means that individuals exposed to copper (B) were redder than individuals without added copper (A).

**Figure 2:**
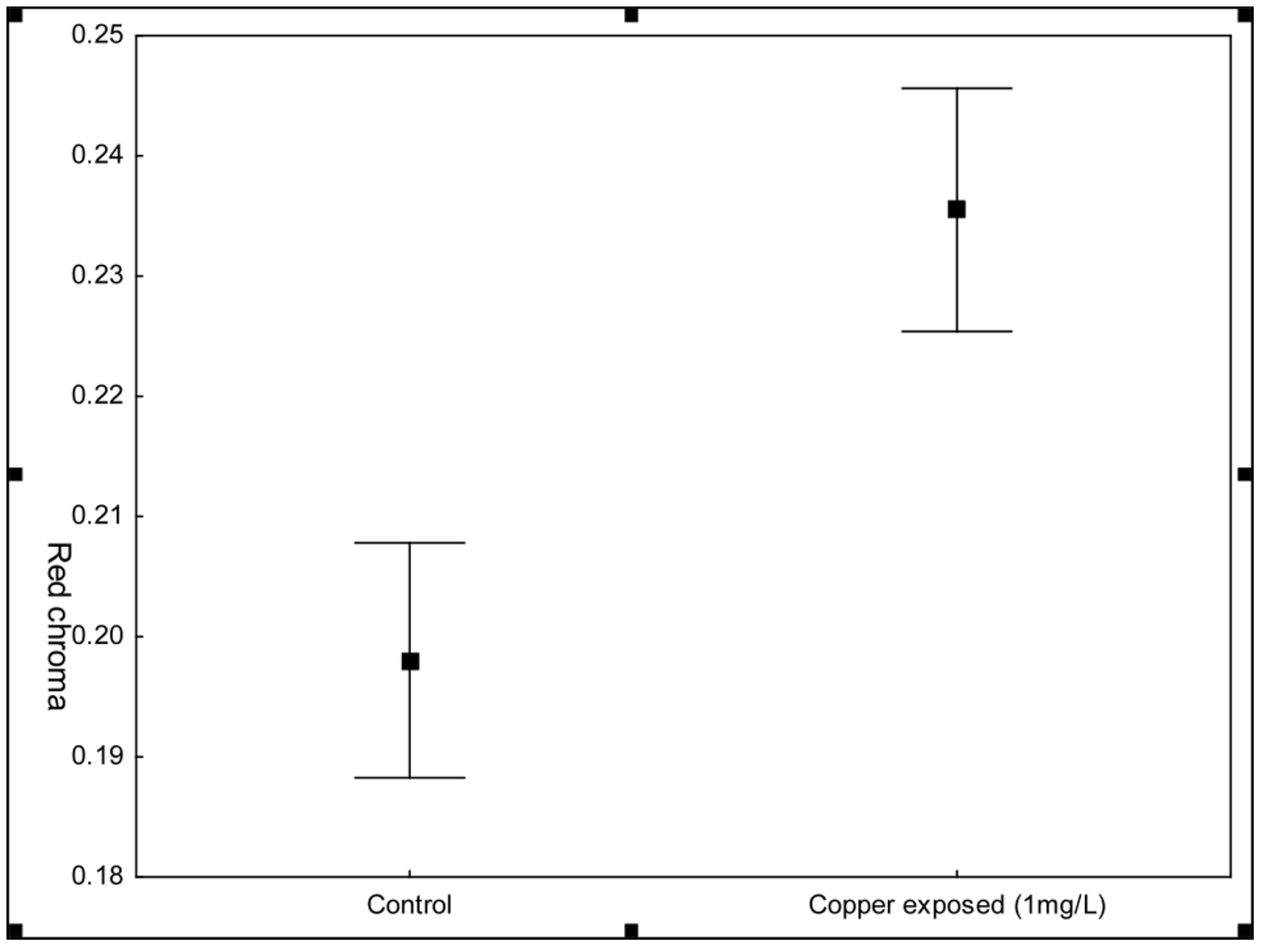
Red chroma. Differences in red chroma in control and copper exposed shrimps.

**Figure 3:**
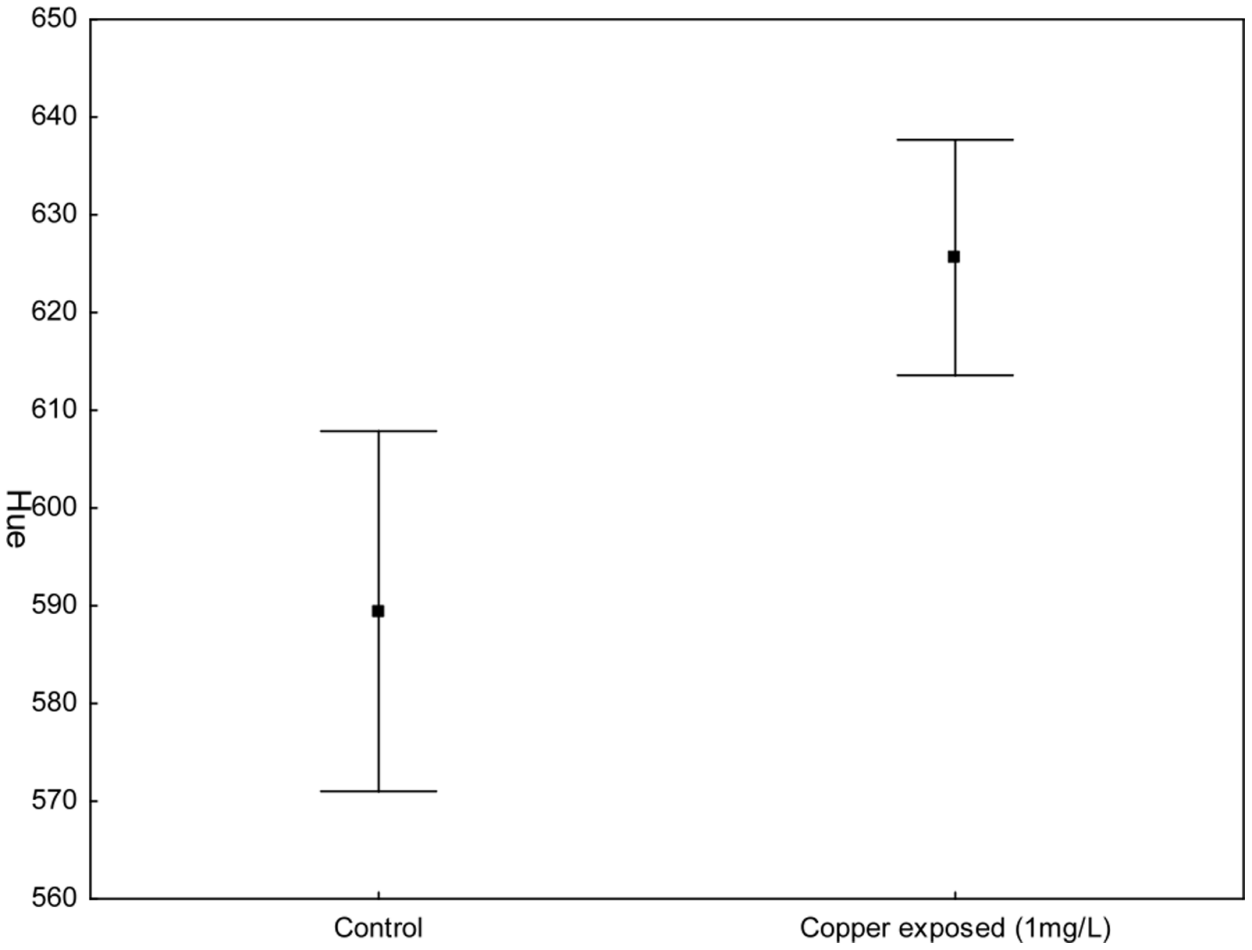
Hue. Differences in hue (between 500 and 700 nm) in control and copper exposed shrimps.

Our results indicate that shrimps are significantly redder in hue and chroma, when copper is present (see Figures 2 and 3). Among all variables tested, treatment emerged as the only significant factor, indicating that the presence of copper affected the coloration of shrimps (hue _(500–700 nm)_: control = 589.44±7.7230; experimental = 625.63±7.8891; GLMM treatment F = 10.75, DF = 1,82, P = 0.0015. Red chroma _(625–700 nm)_: control = 0.198±0.005; experimental = 0.2352±0.005; GLMM treatment F = 28.27, DF = 1,82, P<0.0001. Amount of light did not differ between control and experimental shrimps (P = 0.8335). Neither did time period (assessed on day 4 or 9), tank number or body size affect shrimp coloration (all p>0.66)). These results indicate a clear change in color of shrimps, due to the presence of copper.

At the biochemical level, the precise mechanism that causes more intense red color (hue and red chroma) among shrimps, in the presence of metals is still unknown. One explanation is that shrimps in the presence of copper consume more astaxanthin to avoid the oxidative stress caused by the presence of a heavy metal. However, it is not possible that exposure to a contaminated environment increases intake rate of astaxanthin, because shrimps under experimental conditions are fitted with pellets that do not contain astaxanthin. In fact, experiments indicate that it takes two weeks to observe color changes, directly resulting from dietary manipulation [10], [18]–[19]. These results ruled out this possibility in our experiment, as we detected color changes four days after treatment. One possible explanation is that copper alters the form of astaxanthin. Results from computational chemistry indicate that copper bonded to astaxanthin produces a complex that is redder in color. However, complexities emerge when intra-cell chemical mechanisms that involve organic pigments, are used to explain changes in color due to the presence of metal. Carotenoids are hydrophobic in nature and the concentration of transition metals in the cellular environment is very low [37]. It was reported that transition metal ions such as copper, iron and zinc mainly react with proteins. Considering this information, it is difficult to infer that these metal ions bind to the carotenoids, as these are not available in the cellular environment because they manifest limited solubility in water. All these assumptions need to be clarified. The mechanism causing more intense red color (hue and red chroma) in shrimps in the presence of metals is still undefined. However, it is clear that under experimental conditions, shrimps exposed to copper are significantly redder in color, than shrimps in the absence of copper. Other effects of copper on our shrimps were not analyzed; however shrimps presented normal aspect and behavior.

In summary, the presence of copper makes the body color of shrimps (*L. vannamei*) redder, but more research is needed in order to ascertain; the extent to which our results are replicated in other species and whether the effect is similar in the presence of other heavy metals, whilst also determining the intake mechanism of heavy metals at a biochemical level.

## Supporting Information

Table S1 **Hue, Red Chroma, Lightness, Size, Body Weight and Cu concentration of each studied shrimp.**
(PDF)

Table S2 **Absorption spectra for each studied shrimp.**
(PDF)
