# Supplementary material for: The Effect of Copper on the Color of Shrimps: Redder Is Not Always Healthier
Source: PLoS One. 2014 Sep 17;9(9):e107673. doi: 10.1371/journal.pone.0107673 (PMC4167854; doi:10.1371/journal.pone.0107673)
Supplement: Table S1 — Hue, Red Chroma, Lightness, Size, Body Weight and Cu concentration of each studied shrimp. (PDF) [file pone.0107673.s001.pdf]

Table S1. Hue, Red Chroma, Lightness, Size, Body Weight and Cu concentration of each studied shrimp.

Individual Label	HUE	REDchroma	LIGTHness	treatment	tank	time (days)	TL (cm)	BW (g)	[Cu] microg/g
media-4-I-A-cab	528	0.1650	3506.1517	0	1	4	8.34	4.8	175.11
media-4-I-B-cab	615	0.2118	3139.2883	0	1	4	7.54	3.3	116.42
media-4-I-C-cab	678	0.2440	2094.6473	0	1	4	7.76	3.8	139.08
media-4-I-D-cab	609	0.1945	4011.3297	0	1	4	9.28	6.2	31.62
media-4-II-A-cab	675	0.2325	3840.9813	0	2	4	8.14	4.9	71.22
media-4-II-B-cab	675	0.1975	1525.4643	0	2	4	9.18	5.9	61.8
media-4-II-C-cab	627	0.2214	2368.0913	0	2	4	8.75	5.1	124.6
media-4-II-D-cab	567	0.1886	2940.7107	0	2	4	9.24	5.7	168.97
media-4-III-A-cab	672	0.1872	1817.6153	0	3	4	8.94	5.3	22.8
media-4-III-B-cab	675	0.2013	1631.6763	0	3	4	9.21	5.4	156.9
media-4-III-C-cab	531	0.1832	2165.7607	0	3	4	8.06	4.1	118.2
media-4-III-D-cab	531	0.1828	1220.1580	0	3	4	8.7	5.2	70.44
media-4-IV-A-cab	558	0.1964	4493.3693	0	4	4	9.67	8.5	90.35
media-4-IV-B-cab	528	0.1502	2578.5853	0	4	4	9.02	5.2	125.7
media-4-IV-C-cab	528	0.1586	1222.0420	0	4	4	8.77	4.9	188.47
media-4-IV-D-cab	579	0.1948	3350.1793	0	4	4	9.14	5.3	168.48
media-4-V-A-cab	528	0.1893	3629.3120	0	5	4	8.2	3.8	ND
media-4-V-B-cab	543	0.2001	1246.9390	0	5	4	9	5.1	ND
media-4-V-C-cab	696	0.2002	1939.4890	0	5	4	8.65	4.7	39.28
media-4-V-D-cab	606	0.2068	3444.9040	0	5	4	9.94	5.6	191.91
media-4-VI-A-cab	618	0.2069	2852.5670	0	6	4	8.55	4.8	174.87
media-4-VI-B-cab	669	0.2043	2058.9770	0	6	4	7.1	4.2	104.75
media-4-VI-C-cab	546	0.1837	3164.6047	0	6	4	8.53	4.6	324.09
media-4-VI-D-cab	669	0.2269	2883.3200	0	6	4	8.47	6.3	48.19
media-4-E1-I-A-cab	603	0.2324	4555.4047	1	11	4	8.98	5.1	210.37
media-4-E1-I-B-cab	603	0.2019	3266.8113	1	11	4	9.13	6.3	387.42
media-4-E1-I-C-cab	597	0.2163	3294.4317	1	11	4	8.33	4.2	140.49
media-4-E1-I-D-cab	594	0.1975	1685.9677	1	11	4	9.52	6.7	170.43
media-4-E1-II-A-cab	615	0.2792	2528.6097	1	12	4	8.65	5.2	246.71
media-4-E1-II-B-cab	543	0.1781	3617.5470	1	12	4	9.07	5.8	191.07
media-4-E1-II-C-cab	618	0.2330	2188.1593	1	12	4	8.58	4.7	250.44
media-4-E1-III-A-cab	645	0.2444	1812.0430	1	12	4	9.11	5.4	149.4
media-4-E1-III-B-cab	603	0.2501	1280.9703	1	13	4	9.06	5.5	255.51
media-4-E1-III-C-cab	600	0.2250	2270.8457	1	13	4	8.2	4.1	182.73
media-4-E1-III-D-cab	600	0.2437	2408.5413	1	13	4	9.48	6.8	236.77
media-4-E1-IV-A-cab	669	0.2368	1928.2020	1	13	4	7.96	3.8	166.47
media-4-E1-IV-B-cab	618	0.2843	785.8923	1	14	4	9.64	6.7	591.1
media-4-E1-IV-C-cab	696	0.2299	997.1593	1	14	4	9.58	8.1	222.32
media-4-E1-IV-D-cab	534	0.1744	5423.4883	1	14	4	8.28	3.9	268.15
media-4-E1-V-A-cab	615	0.2108	3904.2110	1	15	4	9.86	7	157.75
media-4-E1-V-B-cab	636	0.2148	1998.0863	1	15	4	8.53	4.6	218.81
media-4-E1-V-C-cab	615	0.2114	1406.5220	1	15	4	8.54	4.9	221.73
media-4-E1-V-D-cab	690	0.3024	1392.0387	1	15	4	9.53	6.2	161.32
media-4-E1-VI-A-cab	597	0.2798	1851.5337	1	16	4	8.51	4.4	177.01
media-4-E1-VI-B-cab	603	0.2061	1599.9547	1	16	4	9.91	7.8	266.94
media-4-E1-VI-C-cab	699	0.2405	2708.5937	1	16	4	8.23	3.8	288.44

media-4-E1-VI-D-cab	690	0.2619	1495.7397	1	16	4	8.62	5	207.02
media-9-I-A-CAB	537	0.1709	3315.6400	0	1	9	9.35	5.7	350.83
media-9-I-B-CAB	501	0.1929	4492.7797	0	1	9	8	3.7	276.96
media-9-I-C-CAB	546	0.1967	1839.7563	0	1	9	9.08	5.4	353.49
media-9-I-D-CAB	534	0.1935	5116.5880	0	1	9	7.72	3.3	283.39
media-9-II-A-CAB	555	0.2019	1473.9700	0	2	9	10.04	7.4	214.53
media-9-II-B-CAB	678	0.2719	1199.4897	0	2	9	8.74	4.7	307.22
media-9-II-C-CAB	528	0.1330	1930.6970	0	2	9	8.75	5.1	216.64
media-9-II-D-CAB	666	0.2593	1958.8010	0	2	9	6.95	2.5	210.31
media-9-III-A-CAB	684	0.2264	1837.6477	0	3	9	8.96	5.6	350.69
media-9-III-B-CAB	600	0.2392	1711.8650	0	3	9	9.82	6.6	182.77
media-9-III-C-CAB	573	0.1734	2553.3040	0	3	9	10.18	7.6	138.68
media-9-III-D-CAB	528	0.1576	3492.4173	0	3	9	8.11	4.4	163.93
media-9-IV-A-CAB	516	0.1661	1200.9130	0	4	9	8.58	4.9	117.38
media-9-IV-B-CAB	540	0.2072	2335.5753	0	4	9	8.95	7.1	201.37
media-9-IV-C-CAB	669	0.2993	499.6460	0	4	9	8.33	4.4	229.07
media-9-IV-D-CAB	612	0.2139	1494.0320	0	4	9	8.64	5	193.32
media-9-V-A-CAB	693	0.2343	2454.3077	0	5	9	8.84	5.1	230.45
media-9-V-B-CAB	666	0.2448	2289.4973	0	5	9	8.56	4.6	210.09
media-9-V-C-CAB	528	0.1380	1117.9803	0	5	9	9.07	5.7	212.73
media-9-V-D-CAB	558	0.1758	1483.9577	0	5	9	9.18	6.2	234.82
media-9-VI-A-CAB	528	0.1562	1234.3017	0	6	9	8.41	4.7	182.36
media-9-VI-B-CAB	555	0.1976	3379.6797	0	6	9	9.1	5.6	194.68
media-9-VI-C-CAB	540	0.1630	3167.0907	0	6	9	8.38	4.2	141.82
media-9-VI-D-CAB	507	0.1647	1373.6907	0	6	9	9.18	5.5	16.06
media-9-E1-I-A-CAB	636	0.2671	3275.4923	1	11	9	8.49	6.1	214.45
media-9-E1-I-B-CAB	675	0.3039	2498.8867	1	11	9	8.78	4.3	289.96
media-9-E1-I-C-CAB	588	0.1783	4962.9397	1	11	9	8.21	4.1	318.88
media-9-E1-I-D-CAB	630	0.1983	4674.9280	1	11	9	7.54	3.5	195.83
media-9-E1-II-A-CAB	573	0.1783	3600.5963	1	12	9	9.15	5.6	238.18
media-9-E1-II-B-CAB	597	0.2048	4582.2220	1	12	9	7.86	3.1	250.74
media-9-E1-II-C-CAB	675	0.2621	2132.7647	1	12	9	8.05	3.4	41.65
media-9-E1-II-D-CAB	636	0.3017	2618.6273	1	12	9	9.14	5.4	237.68
media-9-E1-III-A-CAB	606	0.2273	2118.0777	1	13	9	9.19	6.2	262.24
media-9-E1-III-B-CAB	543	0.2131	2170.6237	1	13	9	8.56	4.6	351.82
media-9-E1-III-C-CAB	648	0.2228	1668.9633	1	13	9	8.1	3.5	385.43
media-9-E1-III-D-CAB	606	0.2364	2567.2100	1	13	9	10.32	8	244.25
media-9-E1-IV-A-CAB	687	0.2507	2375.2097	1	14	9	9.72	6.5	140.15
media-9-E1-IV-B-CAB	669	0.3037	1320.1367	1	14	9	8.34	4.4	237.21
media-9-E1-IV-C-CAB	660	0.2518	1971.6767	1	14	9	8.38	5.1	144.54
media-9-E1-IV-D-CAB	687	0.2399	1497.4340	1	15	9	8.5	4.5	361.96
media-9-E1-V-A-CAB	636	0.2477	3475.4767	1	15	9	8.31	3.9	373.46
media-9-E1-V-B-CAB	597	0.2091	2307.1823	1	15	9	8.55	4.2	273.15
media-9-E1-V-C-CAB	603	0.2000	1507.0843	1	15	9	8.77	5.6	209.29
media-9-E1-V-D-CAB	654	0.2439	2110.9570	1	16	9	8.31	4.2	375.99
media-9-E1-VI-A-CAB	645	0.2456	2117.8100	1	16	9	8.3	4.2	393.66
media-9-E1-VI-B-CAB	621	0.2377	2116.2307	1	16	9	9.46	5.8	355.32
media-9-E1-VI-C-CAB	624	0.2398	2714.5570	1	16	9	7.15	4.4	ND
