# Supplementary material for: The Effect of Copper on the Color of Shrimps: Redder Is Not Always Healthier
Source: PLoS One. 2014 Sep 17;9(9):e107673. doi: 10.1371/journal.pone.0107673 (PMC4167854; doi:10.1371/journal.pone.0107673)
Supplement: Table S2 — Absorption spectra for each studied shrimp. (PDF) [file pone.0107673.s002.pdf]

Table S2. Absorption spectra for each studied shrimp.

Individual Label	300.05nm	303.03nm	306.01nm	309.2nm	312.18nm	315.16nm	318.13nm	321.1nm
media-4-I-A	3.4853	4.2800	5.3403	5.7233	8.1637	8.6620	7.9987	9.0187
media-4-I-B	0.2247	0.5963	2.7940	4.9927	3.1527	6.5860	6.3950	6.0003
media-4-I-C	-2.0210	-0.0507	0.5740	1.1293	2.5080	1.6707	-0.6107	2.1277
media-4-I-D	1.0417	1.3260	3.3040	5.8693	7.0857	4.9720	7.6617	6.9773
media-4-II-A	2.6857	4.2350	5.5783	5.4327	7.3047	7.7550	7.7560	6.4357
media-4-II-B	1.6960	1.4727	2.8903	3.4137	4.4687	5.6933	5.9057	6.5173
media-4-II-C	-4.7190	-6.0700	-1.5277	0.0100	0.4120	0.4877	-0.1540	0.5493
media-4-II-D	1.3847	3.2620	3.7763	8.4753	4.5963	4.9057	8.0353	7.1137
media-4-III-A	1.3047	2.7740	5.6717	5.6773	5.6840	4.9140	4.5457	7.1503
media-4-III-B	2.6307	3.0647	2.2767	3.4383	4.5130	2.7147	5.7390	5.1447
media-4-III-C	-1.9247	-0.3827	-2.3713	1.6640	2.3457	0.6107	2.4810	-2.0797
media-4-III-D	-5.9213	-1.4837	-4.3187	-1.4893	-3.1227	0.0067	-2.9710	-3.0040
media-4-IV-A	5.2567	9.1453	10.0207	7.3553	10.1577	8.6263	12.1030	10.2923
media-4-IV-B	-2.5787	1.2280	2.4820	5.0880	3.7663	2.8613	2.5817	2.8790
media-4-IV-C	-4.6083	-2.2837	-6.6220	-0.8987	0.1550	1.9910	-1.3840	-1.3323
media-4-IV-D	2.3727	3.4127	4.9797	8.8037	8.0873	5.4053	8.4363	8.0013
media-4-V-A	-6.8863	-5.1600	-6.8557	-0.9937	1.4570	-0.9690	-1.3667	-0.7897
media-4-V-B	-6.6107	-4.5460	-3.9943	-1.1140	-0.1253	-2.0770	-4.4547	-3.2377
media-4-V-C	2.5207	4.5620	4.7397	3.5857	4.6700	5.9217	5.4297	6.4407
media-4-V-D	1.3003	1.1570	0.1340	4.5857	4.4463	5.6973	6.3357	2.5700
media-4-VI-A	6.1710	5.3663	7.2820	8.2940	10.6507	10.4960	8.9343	11.0200
media-4-VI-B	2.0333	4.0203	6.0043	3.7887	5.0337	5.6247	6.5877	5.8877
media-4-VI-C	1.4263	0.8203	3.1680	3.5913	2.2543	3.3803	4.1797	1.7283
media-4-VI-D	0.4173	2.8263	1.1940	5.6863	5.7683	5.3580	6.5710	4.7680
media-4-E1-I-A	1.2883	1.3733	1.2740	2.6270	1.7400	3.8780	0.6043	1.8177
media-4-E1-I-B	2.9233	1.1073	1.8747	5.5757	3.5790	6.3133	3.7220	6.1970
media-4-E1-I-C	0.7937	0.1797	1.2680	2.1717	2.3727	2.2937	2.8767	4.6340
media-4-E1-I-D	-4.4840	-2.7697	-4.3453	0.5570	-0.3437	-2.4313	0.3703	0.3623
media-4-E1-II-A	2.1057	0.3310	3.4973	4.3543	3.3093	1.0867	3.5983	3.3957
media-4-E1-II-B	1.1710	0.8903	3.4273	1.6823	2.5503	2.9057	3.0857	3.4707

media-4-E1-II-C	-1.1057	2.2673	0.7977	3.8000	3.4123	1.7293	3.3703	1.2913
media-4-E1-III-A	1.2760	1.3900	2.5183	2.1720	1.4963	2.6680	5.2697	2.4960
media-4-E1-III-B	-2.9960	-1.4260	-4.3720	-1.6143	-1.9463	-2.1640	-0.7367	-1.7087
media-4-E1-III-C	-4.4550	-2.0777	-4.9130	0.0003	1.9517	2.1440	2.2197	0.1607
media-4-E1-III-D	0.2557	2.0523	2.2803	2.2753	2.7797	1.9173	4.3370	3.5403
media-4-E1-IV-A	4.3897	5.6770	3.8293	6.4470	7.4343	5.9587	7.5447	8.3413
media-4-E1-IV-B	-2.6733	-3.4570	-3.8420	-2.3293	-1.5590	-3.0810	-2.9527	-3.0377
media-4-E1-IV-C	-1.0787	-2.7287	-2.5510	-1.5133	-2.5747	-4.1953	-5.1130	0.3710
media-4-E1-IV-D	18.2483	18.6250	20.4007	20.3180	19.4993	18.8930	19.4463	20.7040
media-4-E1-V-A	6.8600	7.3883	9.8997	11.8883	12.7333	13.5113	7.4813	12.0443
media-4-E1-V-B	3.6730	4.3283	5.4807	4.3613	6.3983	3.8323	5.3170	3.6757
media-4-E1-V-C	1.4823	-1.5293	-0.8230	0.5843	0.7753	2.1547	-1.2840	1.7380
media-4-E1-V-D	2.6363	2.2827	1.0663	0.9690	1.8953	2.3317	1.6963	2.8520
media-4-E1-VI-A	-3.6413	-4.7727	-5.7403	-2.2877	-0.4253	-5.0617	-1.1713	-3.9677
media-4-E1-VI-B	0.2830	-2.1900	-1.6293	0.4710	2.1447	0.1953	0.6897	1.9413
media-4-E1-VI-C	6.6283	6.3813	8.0460	7.5940	7.6543	6.3703	9.2367	8.6367
media-4-E1-VI-D	-0.3890	-0.7967	-0.3273	1.4480	1.3390	1.2683	1.3863	0.2537
media-9-I-A-CAB	-0.7943	0.7950	2.9623	4.5783	4.7387	4.8647	7.2440	6.8877
media-9-I-B-CAB	7.1663	7.5777	9.1270	9.6273	10.9560	13.1210	14.8447	13.9380
media-9-I-C-CAB	-0.7337	-0.2373	-0.1183	0.6520	1.6760	2.0607	2.9390	1.8597
media-9-I-D-CAB	0.1267	2.6257	3.7533	5.9737	8.0110	8.1443	9.3637	9.8603
media-9-II-A-CAB	0.6950	1.6240	2.9170	3.5643	3.0180	3.5557	3.3327	3.2883
media-9-II-B-CAB	-0.5087	0.0740	-0.2853	0.2363	0.1397	0.6707	0.4530	-0.3883
media-9-II-C-CAB	-4.6857	-4.8683	-4.9923	-1.8810	-2.1613	-1.6373	-3.5370	-2.8760
media-9-II-D-CAB	1.9117	1.7043	2.1767	1.9083	3.2100	3.7770	3.0113	3.4153
media-9-III-A-CAB	2.5240	1.9520	2.5293	3.5850	4.3007	4.9363	4.4353	5.9830
media-9-III-B-CAB	-0.5510	-0.6923	-1.9427	0.6380	0.2367	-1.1113	0.7517	-0.9557
media-9-III-C-CAB	-0.5933	-2.0097	-1.7740	0.3507	2.7900	1.0190	-0.5563	2.1853
media-9-III-D-CAB	0.7733	2.3710	4.4730	3.8803	5.7310	5.9753	7.3237	7.9233
media-9-IV-A-CAB	-1.3507	-0.8773	-1.1237	0.2707	0.4707	0.3747	1.4147	-0.0500
media-9-IV-B-CAB	-3.5057	-2.5747	-2.4167	-0.5537	-0.4313	1.2810	1.2097	0.5890
media-9-IV-C-CAB	-2.7017	-1.8930	-1.3900	-0.9350	-0.1507	-1.6073	-1.0760	-1.2440
media-9-IV-D-CAB	0.0443	0.6180	0.3987	0.9527	1.0217	1.8723	2.1377	1.3070

media-9-V-A-CAB	1.9297	3.4543	3.1330	3.6173	3.9827	5.7383	5.2133	6.6020
media-9-V-B-CAB	-3.5300	-2.3827	-1.9997	-0.2750	-0.3753	-0.8670	-1.4987	-1.9537
media-9-V-C-CAB	-5.1813	-4.5940	-4.3367	-2.3083	-1.9237	-4.7913	-4.9623	-3.6910
media-9-V-D-CAB	-2.2863	-2.9590	-1.7593	0.3460	0.3657	0.2530	-0.1397	-1.1750
media-9-VI-A-CAB	-3.0320	-4.0420	-2.9333	-0.3870	-1.1927	-3.3343	-1.1147	-4.7837
media-9-VI-B-CAB	3.4897	5.8113	5.4130	7.2130	9.4327	8.2140	9.0060	8.5420
media-9-VI-C-CAB	-0.1050	0.9647	2.5913	5.0910	4.6237	6.0497	4.0250	4.3550
media-9-VI-D-CAE	0.7760	1.9380	1.5617	3.3150	2.8583	3.1603	4.2173	3.5603
media-9-E1-I-A-C/	2.3700	5.7087	6.4213	5.9947	8.8273	7.8970	8.0973	7.3040
media-9-E1-I-B-C/	2.4090	3.6063	5.2933	4.9557	5.4477	6.3237	4.5157	5.2490
media-9-E1-I-C-C/	4.1347	6.0590	6.7017	10.5283	10.6340	12.7210	14.4957	12.9533
media-9-E1-I-D-C/	4.1387	5.9577	6.6430	9.1087	12.2833	13.8347	14.5403	15.0453
media-9-E1-II-A-C	1.6570	5.0163	3.8590	7.0323	6.3377	6.8620	8.9003	5.0240
media-9-E1-II-B-C	3.6717	4.6950	6.6363	9.9017	9.1513	10.4920	10.6477	9.9710
media-9-E1-II-C-C	1.3867	2.9980	2.9637	3.7427	4.5643	5.1433	4.6487	5.4857
media-9-E1-II-D-C	1.1977	1.3183	1.4723	3.4570	3.7277	4.3077	3.6577	3.1253
media-9-E1-III-A-(	-3.3977	-0.5300	-2.1500	0.5577	0.0170	1.6287	0.1390	-1.2637
media-9-E1-III-B-(	-0.5607	1.2267	0.8277	2.7353	3.9920	3.1417	2.0987	3.6883
media-9-E1-III-C-(	2.2383	2.3327	3.9633	3.7673	5.4653	5.9220	5.7143	5.5987
media-9-E1-III-D-(	0.2977	-0.9790	-0.0850	2.2593	1.4287	2.1323	3.2560	1.2307
media-9-E1-IV-A-(	3.1523	4.4640	5.6357	5.8513	7.3960	6.5913	6.8250	6.9137
media-9-E1-IV-B-(	-0.3247	-0.2280	0.5603	0.4757	0.4627	0.9897	0.8540	1.0283
media-9-E1-IV-C-(	-2.8603	-1.5797	-0.6767	1.0717	0.5427	-0.2440	2.3007	1.1110
media-9-E1-IV-D-(	0.7110	0.9537	1.4613	0.7487	2.6153	2.9787	1.0867	1.4957
media-9-E1-V-A-C	4.7803	4.2997	6.6223	9.1317	8.0387	10.0410	9.6507	10.4190
media-9-E1-V-B-C	-0.4160	-0.5507	0.2997	1.7047	3.2760	2.7953	2.3140	3.0027
media-9-E1-V-C-C	-1.0917	-0.8253	0.0383	0.5937	-0.4167	-0.3820	0.0713	1.3067
media-9-E1-V-D-C	1.4840	2.0560	4.2650	3.7747	3.2593	4.0133	4.6767	4.6953
media-9-E1-VI-A-(	3.5680	3.3430	4.0477	4.5043	4.5343	5.6483	4.9793	6.5830
media-9-E1-VI-B-(	0.6133	1.3753	1.9780	2.6607	3.7890	2.4230	4.6483	4.9250
media-9-E1-VI-C-(	1.0660	1.2323	3.4670	4.6153	3.2850	4.9413	5.4247	3.5613

324.07nm	327.04nm	330nm	333.18nm	336.14nm	339.1nm	342.06nm	345.01nm	348.18nm
7.0413	10.0793	9.4573	9.0420	9.4530	10.0713	11.6590	15.2040	15.4983
8.0080	7.3550	8.2013	8.9287	8.5233	8.7303	8.5167	10.8227	13.7183
3.0470	3.5373	2.7373	1.8010	1.3280	3.2110	3.1977	4.7123	4.4083
7.4307	8.2610	5.2873	7.0790	9.1700	8.3320	12.3927	12.1200	16.1177
6.6853	7.5323	7.4693	7.6173	8.5593	9.9800	9.2583	12.3763	15.7333
5.8690	6.9617	7.5247	6.3280	7.1700	6.6097	7.1277	7.4007	7.9727
0.7933	-0.2247	0.2757	1.3993	0.2637	-0.6890	4.6320	3.1153	5.1910
9.6790	8.5877	5.9207	8.6530	8.3217	11.1627	10.1017	11.7693	12.0903
7.6697	7.2390	6.4123	6.0160	6.6373	5.4763	7.0370	8.5253	9.1010
3.9087	3.6783	3.2017	3.5020	5.6140	4.2473	5.5440	5.3660	5.6620
1.4780	0.1577	1.9110	2.1727	1.7230	3.9257	1.7810	2.0907	6.5640
-3.2897	-2.0457	0.2307	-2.6453	-0.8390	-1.3197	-1.3877	0.6270	-0.1767
8.2337	11.4013	10.1503	9.4980	11.5140	12.4220	12.1737	15.2740	17.4490
2.2940	4.7117	6.1673	2.4907	4.6490	2.3573	6.1593	7.6527	8.9830
1.3820	-0.7953	-1.2937	-0.8040	1.3193	2.6157	2.0493	2.8343	4.1533
7.7887	8.9677	8.6770	9.1910	7.3267	8.1310	9.3627	12.0343	14.1090
0.6843	-1.5240	-0.4093	2.5350	1.0437	3.3920	7.4463	8.7343	10.4433
-0.7900	-3.0160	-2.5980	-0.6670	-0.7230	-0.4040	0.8490	0.9297	3.2050
4.9153	5.8487	8.0583	6.5380	6.2167	6.2387	7.7930	7.8097	8.3540
5.6873	4.7087	6.7673	4.4953	4.4263	7.7060	6.9233	7.5983	9.4180
7.7630	7.3733	9.0880	9.3410	9.5043	11.5983	11.2073	11.4273	15.4773
4.4910	5.1963	6.1793	5.0013	8.3943	6.2237	8.6117	7.9323	9.7380
-0.1663	3.6683	2.1600	6.7273	4.8953	6.0587	7.0187	8.6730	8.2440
5.5327	5.1990	5.9343	6.1913	8.2243	9.9230	9.3827	9.7240	12.5737
2.8633	1.6927	4.7233	4.3917	3.2000	8.1587	7.8603	7.7337	8.6590
7.6010	5.5310	4.3273	6.7607	3.5290	7.7290	8.4873	9.7140	9.6467
4.1190	0.7883	1.9720	1.7403	5.3660	4.6320	3.5573	6.8683	9.2313
2.3700	1.0283	2.1843	1.6670	3.4413	3.5943	3.6813	5.8943	6.2920
2.0977	5.2000	3.4263	3.9850	5.3193	7.5277	9.8490	7.8633	7.6567
6.2060	1.6707	5.1593	4.4570	3.5600	5.5607	7.4753	9.1043	13.0760

1.3983	2.2517	1.9293	3.2607	2.6463	6.3203	6.2030	6.0207	7.4983
3.0337	4.5447	3.2730	3.2413	3.8597	5.1950	5.0337	5.1547	6.6693
-0.7557	-2.1130	-0.8260	-2.0753	-0.8537	-0.4117	-0.9550	1.0930	1.3807
1.1363	0.5163	-0.2283	0.5833	4.0740	4.4650	6.5623	6.6593	8.6880
3.9293	4.1910	3.8637	3.4773	4.4617	5.3993	5.0407	6.4373	7.9040
5.2220	7.0143	6.6780	6.8847	7.5507	7.1543	9.5947	7.7547	7.5330
-1.3280	-0.6840	-1.1927	-2.3820	-1.3580	0.1687	-0.8230	1.6380	1.1780
-1.4667	-0.5883	-3.5003	-2.2990	-2.3240	-0.5357	-0.2717	0.0577	2.4750
20.0350	19.4197	19.5533	19.0657	20.2713	19.1543	20.0057	22.2583	21.5107
13.2957	13.6223	11.4197	11.9030	13.9703	13.7707	15.9310	15.5743	16.4893
7.5927	6.1357	5.0773	6.5127	6.9137	5.2020	7.6840	7.2180	8.2237
1.2080	2.2140	1.1160	-0.4813	1.1257	0.4453	2.0163	2.4403	4.9020
-0.8167	2.8640	2.5947	1.6100	1.1117	2.3770	0.9497	2.1900	2.9617
2.8943	-0.4837	-1.5610	-0.8450	-0.8900	-1.0557	1.4803	2.0463	2.7700
1.6253	0.3577	0.3547	1.8333	0.6500	4.6317	5.3723	4.9817	4.6243
7.5093	6.0680	7.7107	8.2223	8.5040	9.9220	9.0610	8.5217	12.3380
2.9093	2.0557	1.7000	3.0973	1.9403	2.1170	5.1500	5.5670	4.1157
7.3510	8.4953	9.5893	8.2287	10.0060	10.8457	12.2870	13.1170	13.1517
13.0967	14.8813	14.7967	15.2187	13.5107	15.4950	15.2270	16.4840	17.6597
3.0120	2.3230	3.0203	2.4450	2.4627	2.0773	4.1637	4.3477	4.9807
11.1070	9.8570	9.9440	10.0140	10.7307	11.4520	14.7473	15.7457	17.1127
3.5763	2.5927	2.8990	2.3017	3.3203	3.7100	4.3273	4.7930	4.9003
0.0023	1.1963	1.3467	-0.4523	1.4310	1.1263	1.9973	1.8223	1.6130
-0.5047	-2.2750	-3.0147	-1.0937	0.0220	-0.2047	3.2890	4.4960	2.5917
4.1447	4.4880	4.8383	4.7330	5.2640	5.5413	6.0657	5.7080	5.8650
4.8917	5.2517	4.9293	5.6530	5.2913	6.0673	6.1150	6.3983	7.4253
-0.2710	0.6943	0.1860	0.5540	0.3603	0.6360	2.1923	2.4593	3.2977
3.1513	2.2327	-0.4570	0.3067	2.8900	5.4907	2.2627	7.5267	6.5030
10.9610	8.4263	7.8823	8.5457	8.8557	9.5010	11.2470	12.3387	14.1380
0.9260	0.8633	0.7407	1.8470	1.9777	2.5360	2.2617	3.0900	3.3013
1.8220	0.3280	0.2383	2.6563	3.0523	3.6777	5.2210	5.0757	7.2123
-0.7937	-1.5243	-0.8777	-1.6253	-1.7517	-0.5010	-0.0713	0.4573	-0.8243
0.6027	1.3913	1.8223	1.6373	2.0877	2.7967	2.1900	3.8427	4.4530

6.4133	6.1350	6.2513	6.8910	6.7137	7.6677	8.5350	9.0870	11.0340
-1.4937	-0.9387	-2.3013	-0.2550	-1.0877	0.7747	1.0207	3.0357	4.2763
-1.1000	-1.4577	-3.1953	-0.7207	-0.7400	-0.3233	1.9923	1.9343	2.3017
0.7627	-0.5610	0.2613	0.0510	0.4073	1.7103	3.6093	3.7747	4.6070
-1.5743	-2.8047	-1.8690	-0.4733	0.1330	-0.0657	0.5943	-0.0163	2.9897
7.6537	10.3230	9.2780	9.0547	7.7243	11.5157	11.4350	11.0943	13.0940
6.1973	6.0240	7.0150	6.9077	4.8500	7.7913	8.9680	10.0760	10.6007
3.5203	3.1163	3.7773	4.3753	4.0887	5.0510	4.7027	5.9597	7.1787
5.7777	7.3840	6.9360	8.9853	8.7597	8.4510	9.4270	10.3133	11.8030
5.2777	5.0947	3.6983	3.4753	6.3367	5.5810	5.6393	6.9637	8.5010
11.2920	14.8363	13.9440	14.3020	16.0280	17.2400	17.5907	20.3477	23.3283
15.4947	15.4623	11.9207	14.9860	16.4880	17.0587	18.9710	21.8860	23.1790
6.3700	9.8303	7.1207	8.7483	8.0237	11.6690	12.1177	13.4313	14.7363
10.6307	10.3143	10.9253	10.6253	13.1343	11.9550	13.7453	13.9197	15.1047
5.0280	6.2253	6.5817	5.9987	5.4743	5.2133	6.7107	7.4337	7.6363
3.6653	3.2180	4.1877	3.7160	4.2967	4.3273	5.5793	6.0213	7.4207
0.5853	-0.1767	0.5150	0.3867	2.4303	2.6380	2.6450	4.2553	8.4253
3.5210	5.1993	2.6597	3.3100	4.3383	4.9570	4.7137	7.6583	7.6770
5.6187	6.2223	5.1013	5.5303	6.4913	7.2457	7.5247	5.9820	8.2053
1.8530	1.9487	3.5267	4.2217	4.6770	5.4073	4.9493	6.5723	7.8507
6.9107	6.7177	7.2993	7.3060	7.8100	8.2483	9.5843	9.5913	11.0890
1.1657	1.3453	0.2910	0.3867	1.4227	2.3380	1.0290	1.7310	1.5440
1.5720	2.7763	0.3970	2.3597	2.2140	2.2007	3.2913	1.7710	2.0483
2.9113	1.8857	2.0290	2.7337	2.8193	3.0627	3.3223	2.9510	4.2680
11.8240	10.3150	10.7527	10.4040	10.6997	11.4980	11.7207	12.3560	12.3037
3.3333	3.4947	2.3633	3.6060	4.8990	5.2863	5.7187	6.7497	8.1437
1.6597	0.9827	0.2747	1.5823	0.7800	2.4660	2.8943	3.4280	4.1410
3.1767	4.2320	4.5987	4.6800	5.2647	5.4550	5.6907	7.1253	6.9287
5.3637	5.0317	5.6167	6.8523	7.2930	5.8570	7.0153	7.5250	8.4520
4.7523	4.8123	5.4100	5.0340	4.1987	4.8770	6.5260	6.7257	7.8167
3.8217	4.8113	4.0733	4.1187	4.7890	5.8290	6.3900	7.6477	8.3677

351.13nm	354.07nm	357.02nm	360.17nm	363.12nm	366.06nm	369.2nm	372.14nm	375.07nm
17.9200	18.8170	22.6107	25.1263	27.1150	26.2033	27.2377	28.0200	27.6513
13.3900	16.1423	17.9380	20.1563	18.6797	20.3703	20.9083	22.7243	25.0193
5.2547	6.9430	8.0910	8.5290	8.8570	9.9387	9.1150	10.6643	11.4753
18.3850	20.9930	24.0917	26.7260	27.9623	29.0020	30.7413	32.3177	32.5870
18.9463	19.0197	20.6463	21.7793	24.3493	24.9940	26.7837	26.8540	27.9673
7.8597	9.4247	10.1393	10.2263	10.8067	12.6493	13.2740	12.2017	12.8067
9.8523	10.4333	13.3910	14.1547	13.3873	15.6323	16.5030	15.6723	16.2037
13.7647	17.7213	17.6343	19.0337	20.7967	21.1213	22.7090	22.3147	22.0800
9.0513	9.1560	9.1023	11.5303	12.2640	12.3980	12.8847	13.4203	13.4203
7.3503	7.9950	8.6190	9.1597	9.5230	9.7493	10.1650	11.6137	11.7520
7.5403	7.6177	9.1210	11.0147	10.8210	12.9547	12.8207	13.3177	15.1777
2.3407	3.4513	5.3870	4.7893	5.3863	6.3427	4.3790	6.6007	6.7053
22.7347	24.9093	27.0647	30.5877	31.3433	34.0237	34.8520	35.6600	35.8863
7.7870	11.5353	13.4883	15.3160	15.6057	17.4663	21.3393	18.4260	22.1880
4.6543	6.3473	8.5120	9.6913	9.6143	9.6033	11.4673	10.1860	11.2213
17.2233	20.6217	21.4390	23.8123	24.1310	24.7003	26.2800	27.5263	26.3590
10.6097	15.1390	19.0820	21.7030	23.4517	20.8057	21.9240	23.2900	24.9447
2.6263	4.4940	5.8587	6.4680	8.3040	8.3700	8.2157	10.4157	10.7010
9.1750	11.2763	10.8447	12.0783	12.4357	14.7400	14.2183	14.6860	15.6953
11.3723	15.7200	14.9060	19.5473	19.6023	21.2603	21.4993	23.5007	24.8870
15.2723	15.5613	17.2130	18.7873	21.1737	21.6883	21.4667	23.2747	23.8403
12.1107	12.0697	11.8157	13.0147	14.5523	15.3120	16.2487	15.2320	16.8787
15.4753	17.9090	19.6690	22.1430	22.9433	23.6657	21.5650	25.8567	25.4510
13.6403	16.1177	16.6263	17.9130	18.8930	18.5470	21.2510	20.3347	22.3373
11.9943	12.7907	17.1230	19.2023	19.9727	21.1127	20.9187	23.8193	23.7277
12.1973	16.3307	17.5273	17.9830	21.8597	21.6673	23.4257	23.2443	24.5280
10.7350	14.2283	14.9517	18.0113	18.9527	20.2007	21.2000	21.2750	24.0803
7.0813	10.0013	10.7363	10.8567	12.5220	12.0213	13.1390	12.9537	14.1543
7.7000	10.4883	11.3903	10.3457	14.2330	13.4447	15.0240	15.8050	14.7490
14.8583	19.0880	23.4010	24.7003	27.3687	28.4330	28.2217	30.7160	28.9280

10.0773	9.5383	11.4437	12.8070	13.3373	15.1717	15.8330	15.1990	14.9950
6.1133	6.6037	7.5100	8.3037	9.4080	9.9267	9.9897	10.3807	10.7960
2.1377	3.0930	3.4980	4.9947	5.7500	6.1357	7.1943	6.4020	7.3733
9.4643	9.4867	12.6770	13.3673	13.6083	14.8420	15.3790	16.0980	17.5147
8.3500	8.5423	9.6810	11.2537	12.1210	12.2137	14.4243	13.0663	14.5967
10.2400	10.5043	12.0727	10.9493	12.1467	10.8893	12.9990	11.6300	13.4023
0.7390	1.8643	4.5413	4.9040	3.9093	4.6433	4.7610	5.1047	5.3207
4.4810	4.0093	4.2337	6.0100	6.2883	6.1550	6.9157	7.7897	7.7200
26.9583	27.5027	29.1937	28.7070	31.2943	31.3740	31.7917	31.5703	33.1863
19.4923	21.8610	20.3980	22.6197	23.9007	25.6637	26.1433	28.4610	28.6637
8.4873	9.7243	9.2020	11.3337	12.9003	12.6297	13.8707	13.9903	12.9370
5.6673	5.9417	8.0393	8.8743	9.9683	10.0067	11.2660	12.0777	13.8397
3.3823	4.4570	4.3197	4.3333	5.0017	4.8807	4.5010	6.5097	5.4190
2.3660	5.2860	5.7000	7.7213	8.0987	7.7080	5.5520	9.6300	8.3650
5.7270	7.0173	8.6150	10.1383	11.2740	10.4830	11.5677	14.2500	14.2150
12.7463	13.1763	14.8297	15.6383	16.3450	16.3407	16.1193	18.5490	15.8003
5.6663	7.7760	7.6993	8.0847	7.7433	10.0563	10.1443	10.3290	10.5080
15.1803	18.1283	20.0923	22.3023	22.4663	22.0190	23.5030	25.3963	25.1330
19.0843	20.9543	22.1153	23.9150	24.7410	25.7920	27.4343	28.5923	30.8250
6.2400	6.6433	8.5687	9.0127	9.9447	10.4577	11.4553	12.2827	13.5203
18.7027	20.9713	23.7487	26.8140	28.5093	28.1327	29.3197	32.0890	31.7450
6.5570	6.6920	7.5710	7.8737	8.6480	9.3127	9.6693	10.4133	11.0313
2.9450	3.3667	3.1880	3.4660	3.8753	4.9100	4.4350	4.7510	5.6017
7.9017	8.6757	11.6547	13.9487	15.1590	15.4760	15.3467	16.9293	16.7527
7.7543	8.4050	8.1440	8.7750	8.8613	10.1233	9.9467	10.2237	10.3753
8.3870	8.8980	8.7433	9.5913	10.5833	11.2673	11.8057	11.1450	11.7860
5.9250	3.8810	6.0953	6.1580	8.3273	7.2470	6.4867	8.4030	9.0910
10.0560	12.2190	14.0243	15.0970	16.6373	18.5690	19.4843	19.3523	20.7847
15.6750	17.8083	21.0070	22.0313	23.6110	24.0923	25.9430	27.4513	28.3090
4.6923	6.0647	7.5223	8.5190	9.7800	10.1290	9.6430	10.7590	11.5823
8.6267	10.2970	11.8417	13.7977	15.1353	16.2733	17.2117	18.0430	18.0760
0.5360	0.1103	0.6163	0.7717	1.5900	0.7807	-0.3120	1.5283	0.9143
4.7337	5.8837	6.5473	7.5393	8.2123	9.6517	10.2467	10.1587	10.7747

10.8080	11.6367	12.2407	12.7923	13.5680	14.9610	16.1347	15.9783	16.6827
5.6983	6.6940	8.6277	10.8283	11.9387	12.7790	13.5567	15.3673	15.3140
3.7530	3.3233	6.2157	7.5673	8.1857	8.8573	9.3467	9.7003	10.7230
6.6720	9.6280	10.6767	11.9410	12.2547	14.1740	15.4730	15.5400	15.6063
2.8793	4.0440	4.3350	6.1310	6.4900	6.8813	6.1383	6.2450	8.0007
15.6593	15.6743	18.4827	20.5703	22.0490	22.7473	23.1850	23.4103	25.2427
11.8543	15.1087	16.7940	19.4313	20.0383	22.9707	21.9433	23.1070	25.7483
6.5340	6.3890	7.4347	7.3950	8.5110	9.1340	9.7270	9.7550	10.6850
14.5823	16.1693	16.0700	17.1950	18.0383	17.6423	16.4303	17.6553	16.9887
9.3733	10.5257	12.0423	11.8577	12.7017	14.3597	13.6117	14.8137	14.9867
26.1670	26.8357	30.3280	32.7550	34.3847	34.5127	35.9567	35.1420	38.1397
25.8190	29.2893	29.9227	32.1700	35.2933	35.9383	37.0673	39.4783	39.0290
18.3493	19.1063	22.3533	24.3830	25.5097	28.3977	26.6497	27.4877	29.4880
17.1413	20.7147	22.0597	24.4133	25.4357	25.1663	25.2723	27.3620	27.7577
8.7807	8.9633	8.8863	9.8367	10.1777	9.6557	11.1163	11.1223	11.7050
10.3250	12.0777	11.9960	12.8810	14.8150	14.5197	15.4207	16.3830	16.0500
8.2517	10.6670	11.4093	13.5243	13.0000	14.1563	14.6253	16.2117	15.9773
8.1373	10.6183	11.2320	12.0420	11.8490	13.7507	13.2260	13.8820	14.1917
8.8900	8.6927	9.3820	9.9643	10.0630	11.6147	10.9330	11.6800	11.8803
9.8833	10.4450	11.8157	13.0440	13.0640	13.8470	14.7070	15.9003	16.1220
11.8270	12.4000	12.9283	12.9493	13.4873	14.5747	13.8730	13.9850	14.6523
2.5347	3.5407	3.3837	3.9037	4.1127	3.9197	3.9583	4.0003	4.2637
4.0763	6.4270	6.2857	7.8557	8.8037	8.0297	9.2907	8.5387	9.7483
4.0303	4.6467	4.6317	5.6817	5.2260	5.7067	6.9543	6.7100	7.8770
15.4717	15.6170	15.6487	16.2517	17.6390	18.1397	18.6693	17.7837	20.9167
9.3680	10.3333	12.8460	13.6580	15.3107	16.4800	15.7107	17.1923	17.3697
5.3933	7.2120	7.8517	8.6167	9.3477	9.6010	9.2427	10.8223	11.2767
7.9697	8.8103	9.5630	10.2673	11.0680	11.1050	11.3937	12.4670	12.1517
10.1413	10.3890	10.2647	10.7250	12.0323	12.3423	12.4457	13.2187	13.4010
9.1850	9.1740	10.9870	11.2457	13.6827	12.1900	13.5207	13.6170	15.0737
10.3950	10.6670	12.2893	13.3293	13.8967	14.0880	15.2780	16.0110	15.6677

378nm	381.14nm	384.07nm	387.2nm	390.12nm	393.04nm	396.17nm	399.08nm	402.2nm
29.1373	29.0817	29.9823	30.5037	30.9723	31.5997	30.7427	29.8340	31.2047
22.5227	24.1520	24.4727	25.0477	25.9183	24.4953	25.0693	24.4867	24.6587
12.2097	12.0480	12.3500	13.0870	13.0353	12.4440	13.0047	13.3353	12.8453
33.7183	33.6277	34.8580	34.8773	35.2827	34.8930	34.7003	35.9597	34.8807
27.7753	28.4430	27.5320	28.0353	27.7593	27.2147	27.4937	27.1390	27.3290
12.2997	12.3050	13.0233	13.7007	12.9603	12.7283	13.0007	12.4487	12.3937
17.0097	17.7070	20.6283	18.6603	19.7613	20.3023	19.0957	18.0890	20.0917
22.8607	23.1930	23.5573	24.9850	23.4510	23.8170	23.6237	23.8047	22.6693
13.6047	13.6790	13.5913	14.4023	15.2810	14.4053	15.8973	14.4867	14.6667
11.3730	12.8670	11.9757	13.0200	12.2880	12.5573	12.3107	12.0387	12.6323
15.6050	15.4067	16.4407	16.8720	17.1690	18.1550	17.9750	17.8870	19.0623
10.0857	10.4673	10.0860	9.7037	11.3643	12.3287	12.1507	11.9197	12.0947
36.1103	37.7057	36.3817	39.3803	39.1703	39.5140	36.5940	37.0243	38.2927
21.9690	22.5233	22.1630	23.5473	24.3100	24.9187	23.8760	23.8827	24.5153
12.1280	12.4053	12.7170	13.0713	13.2377	14.2317	13.3810	13.0160	13.9687
27.3590	27.7093	28.3057	28.7120	28.6790	28.3150	29.3497	27.7720	27.9290
26.9953	24.3987	28.6783	28.2797	28.8163	30.2737	29.1573	30.0957	29.1940
11.4060	11.1700	12.3663	12.5307	12.0550	12.8783	13.5113	12.7787	13.5867
14.6750	17.4353	17.9613	16.7893	16.8243	15.8750	16.3903	16.1480	15.6607
24.4647	25.2917	28.4540	28.2110	28.4500	28.6397	29.3540	29.5837	27.6527
23.7367	22.5460	23.8497	23.3673	22.8090	23.0050	22.5133	23.1927	23.0817
15.9313	17.5447	17.8173	17.4540	17.0930	16.7020	17.2477	17.0787	16.7630
26.2273	25.2050	29.8853	28.1567	30.1647	28.5647	29.5530	29.0887	28.9560
20.9827	22.5950	21.6963	23.1813	22.3163	21.3600	21.7393	21.9283	21.4217
23.8953	26.2517	26.7537	30.1233	28.9777	28.8103	30.1950	29.8403	29.7230
26.2983	26.8903	27.6390	27.4977	27.4420	27.5227	27.6423	28.4167	27.7257
25.3030	24.7160	28.3407	27.3590	27.6413	27.6450	26.0140	27.2770	26.0563
13.8770	15.0727	15.1093	15.9287	15.5750	15.2123	15.7083	15.8723	15.1283
17.0873	15.3723	16.6003	16.5523	17.7157	15.9043	17.4483	16.2673	15.9943
30.7190	33.2707	32.3483	34.1103	34.3797	34.1040	33.6540	33.3447	33.7423

15.9680	15.8947	16.6420	16.2763	15.6203	15.1790	15.1267	15.6843	15.2503
10.4360	10.9747	12.4233	12.3037	11.7067	11.8760	12.2080	11.3503	11.5957
7.6827	7.1140	8.1743	8.9347	8.5290	9.2577	8.8030	9.4120	9.0320
16.9900	17.4950	17.8440	17.4713	17.4283	18.4710	17.6303	17.7683	18.6890
14.2177	14.7043	15.6353	15.1517	15.0120	15.7337	15.2177	15.8147	15.2700
12.8583	14.3590	12.3937	11.3503	12.1637	10.9750	11.4643	12.9617	11.5187
5.4830	6.5967	5.6793	6.0923	6.6407	6.2897	5.7723	5.5240	6.7257
7.6990	8.7440	7.9577	7.3660	8.5503	8.0180	8.5047	8.9390	8.1303
35.3927	35.6297	36.6540	37.9603	37.5710	39.1353	39.0597	39.8843	38.6550
29.4620	29.6677	30.5767	29.7220	28.6777	30.2257	30.9967	31.4910	30.6870
13.9370	14.2603	15.0000	14.5020	14.6033	15.0750	15.4563	15.1720	14.8017
12.0093	12.7027	12.1903	12.5757	13.3530	12.1223	13.5910	13.1140	13.7937
5.6500	6.7730	5.5833	7.3303	6.7280	6.0937	5.1397	6.9360	6.7433
10.2983	10.9687	11.9727	11.1293	11.1840	11.6157	11.2077	10.7597	12.0913
13.9463	14.4350	13.8693	14.2700	14.5417	15.6070	14.4923	14.4553	14.6087
17.6357	15.9263	16.7857	17.2690	17.3630	16.7173	17.6007	15.6310	18.5177
10.4193	11.5147	10.6613	10.9433	10.8880	12.0217	12.1270	10.5730	11.2737
27.6807	26.6693	28.5563	29.3390	29.3447	29.6143	30.3520	29.8167	30.8353
31.1490	32.7657	32.5277	33.6353	34.0057	35.3650	34.5177	35.6103	36.0603
13.7533	14.2997	14.0327	14.8140	14.7790	15.6510	15.7773	15.1900	15.3663
33.5557	34.6000	35.5370	36.0310	37.3973	37.7840	37.4240	38.1290	37.6907
11.6093	12.0073	11.6853	12.0133	11.6340	11.6303	12.0560	12.7370	12.3500
5.3653	5.8107	6.2153	6.3023	6.2627	6.4683	6.4240	6.9140	6.4457
19.7800	20.0613	21.2133	21.4710	23.1073	23.3837	23.1763	23.2020	24.6223
10.3567	11.2023	10.4170	11.2647	10.3153	10.3887	10.8240	10.5757	10.3087
12.2133	12.5353	12.5150	12.3413	12.0380	11.7523	12.2590	12.2540	12.5457
9.1467	10.2280	9.3117	9.9883	11.0713	10.5823	9.8387	9.5640	10.4070
22.2663	20.7260	21.9733	23.4340	24.0880	25.0193	24.7693	25.3737	24.8063
27.7790	30.7357	31.1660	33.2537	32.9843	33.9163	33.9510	33.2270	34.7437
11.5433	12.4780	12.6507	12.4153	12.6597	12.5890	13.0333	12.8593	12.8400
18.6123	19.1803	19.2353	19.5420	19.6457	18.9190	19.2277	18.8717	18.0407
1.7763	1.9760	1.9733	2.9050	2.4943	2.4253	3.2857	2.2427	2.5483
10.4777	11.2030	11.8133	11.6033	12.0503	12.0620	12.0113	12.5303	11.7263

16.4590	17.0210	17.1413	17.3830	16.8260	16.5437	17.3443	16.7947	16.6537
16.2780	17.2910	17.6547	17.9263	18.2547	18.4667	18.9053	18.1110	18.3317
11.0110	10.6620	12.4043	12.4443	13.1540	13.6260	12.9250	13.4190	12.9797
16.0930	16.9560	16.8960	16.3500	16.7963	16.9270	17.6047	16.8447	16.5363
8.2843	8.3427	8.8380	9.5983	10.1997	10.6593	10.4653	11.4093	11.3187
25.3817	26.0467	26.0857	27.5980	27.3407	27.3100	26.8380	27.7047	28.3013
27.0790	28.3190	30.3403	29.7593	30.2803	30.8350	30.8147	30.5880	30.7260
10.4740	11.0450	11.1720	11.0960	12.0683	11.5183	11.8293	12.2043	12.6300
18.5583	18.2840	18.5320	19.0480	18.9760	18.0530	18.7027	19.4797	18.2797
14.6357	14.7623	15.0510	14.5287	15.2157	14.7767	14.5680	14.6683	13.6500
37.8517	39.4713	40.8687	40.8063	41.0893	42.1040	41.9807	40.9637	41.6657
39.3557	40.4143	40.2863	40.4853	40.7600	40.4227	40.7153	41.1047	37.8360
30.4180	30.0940	31.5487	31.7993	34.1487	33.1803	33.2270	32.6027	32.7877
29.0483	28.8907	31.2747	30.8547	32.2523	33.0617	34.2533	33.6257	34.2733
10.5947	11.0913	12.0913	11.9060	12.4367	11.5363	12.2337	12.2860	12.1780
17.0540	17.2957	16.6613	16.1127	17.1160	16.3447	16.9453	16.0457	16.5367
16.9793	17.9390	18.5247	18.6707	18.3730	18.0153	19.1310	18.4510	18.1363
15.1217	14.9660	15.0077	16.2073	16.5197	15.7277	15.9717	16.0273	16.4813
12.3673	12.6097	12.7217	12.2407	12.6890	11.6227	12.0873	12.5160	11.7893
17.1053	18.8183	18.3217	19.5173	20.3380	20.4920	21.0133	19.3933	19.8427
14.7377	15.2113	14.8083	14.7363	14.6397	14.5723	15.2647	15.2903	15.0027
4.4037	4.4107	5.0747	4.6730	4.9763	4.8837	4.9360	5.5797	5.0790
11.0393	10.4237	10.6800	12.0123	13.0500	12.9570	12.0390	13.1653	12.7303
7.8873	8.6737	7.7033	8.1853	8.5257	8.4750	8.6333	8.6673	8.6780
19.5063	19.5530	20.2223	21.0863	20.1943	20.1057	20.5663	20.2313	20.9530
18.4957	18.0953	18.3337	18.8173	18.6630	18.3957	18.9237	18.3813	18.7417
11.7643	12.1450	12.1663	12.4007	12.5533	13.1223	13.0937	13.6533	13.4553
12.3533	12.8370	12.9453	13.3893	12.8253	12.6530	13.0510	13.3970	12.9427
13.3767	13.4717	13.8987	13.5243	13.5713	13.1257	13.7673	13.7857	13.5917
14.3787	14.6750	15.3667	15.9460	16.3737	14.3853	15.6980	15.4313	16.4397
16.1960	17.4267	17.3880	17.3430	17.5460	17.9617	18.0570	18.5537	18.3010

405.11nm	408.02nm	411.13nm	414.03nm	417.14nm	420.04nm	423.14nm	426.04nm	429.13nm
31.2773	30.1793	29.6517	29.6473	31.1190	30.5870	30.2497	29.6540	28.0633
24.4433	24.1077	23.1640	23.3800	22.9260	22.5027	22.0753	22.5913	22.3650
12.5680	13.3220	12.8490	13.5663	12.9353	13.0567	12.9663	12.9740	12.5037
34.7523	33.7567	33.8490	33.4907	33.3227	31.6077	30.6503	30.7487	30.9010
25.8343	27.0360	25.5213	25.9623	23.6157	22.2990	22.1490	22.7250	23.5127
11.8017	11.3757	11.3083	11.1333	10.7857	9.5810	9.4723	9.9730	10.2517
19.9493	19.3667	18.8293	18.1780	19.4333	20.6200	20.4757	19.3417	19.1010
22.9953	23.0793	22.8960	22.3420	22.3743	22.3670	21.9560	21.7200	21.7863
13.4063	14.2553	13.6490	15.1230	13.5820	13.7640	13.5410	13.6743	12.6023
11.8820	12.2673	11.6390	11.8213	11.4747	11.4513	11.5100	11.2420	11.5277
18.5413	17.4917	17.2233	17.8803	17.6067	17.9967	18.2603	18.4903	17.3170
11.8233	12.5403	11.1863	11.1923	11.7517	11.9207	12.1910	11.8367	11.5547
36.7470	36.8350	35.4287	35.2987	34.0633	30.8087	31.0150	31.8440	32.5323
24.5673	25.9097	24.3727	24.7943	24.9027	25.2173	25.4400	24.1000	23.9120
13.6327	13.3370	13.2080	13.5570	13.3130	13.4180	13.2947	13.0627	12.7557
28.2840	27.3797	26.3907	27.8073	25.7627	24.9813	25.3177	25.1817	24.7227
31.3497	29.7820	30.0123	28.8813	32.4563	33.9850	33.6733	31.8227	30.7330
14.3680	13.4270	12.7073	13.1373	12.4860	12.3487	12.0233	12.0623	11.4303
15.6970	16.0480	14.6743	14.8507	13.8137	12.7443	12.4587	12.7507	13.3580
29.6573	29.0263	27.8690	26.5620	27.6840	28.0253	27.8883	27.0087	25.8280
21.1543	21.3180	21.1710	21.2543	19.2420	18.3533	18.5727	18.4557	18.5957
15.4273	16.4037	15.8903	16.8587	14.3960	13.7653	12.9913	13.8210	14.4103
29.7953	27.8017	26.9543	28.4933	27.4340	27.7567	26.1153	26.2453	26.7727
20.2023	21.5677	20.8580	19.8963	19.5020	18.6780	18.8893	18.8677	18.8260
29.3447	28.4523	27.2997	28.0687	32.1230	32.4893	33.1830	30.4100	29.0423
27.6787	26.1240	26.0697	26.3730	25.3450	25.8053	25.5460	24.5567	25.3203
27.6573	26.5677	25.0463	25.8957	25.2123	26.0537	25.2907	24.5770	23.8517
15.0793	16.1127	14.3593	14.6393	14.0913	13.8197	13.5317	14.2080	13.6023
14.2300	15.5627	16.1747	16.1993	14.0057	13.9970	15.0970	14.9543	13.8563
33.7207	33.1443	32.5683	32.4043	32.6323	34.0020	32.7473	31.4883	30.4570

15.2113	14.9727	14.6693	14.6517	14.3847	14.1583	14.2917	14.3870	13.5307
11.3717	11.7033	11.4333	10.9007	10.4560	10.3107	10.0370	10.4303	11.0253
8.9457	8.4933	8.8737	9.0873	9.0890	9.3347	9.0723	9.2323	8.3977
17.0783	17.4607	18.0987	16.9840	16.5253	16.0890	15.7470	15.7757	15.6680
15.3807	14.4113	14.5030	14.5467	14.1023	13.9593	13.6760	13.7207	14.1683
11.9503	11.7527	11.4707	11.7983	11.1567	9.6390	9.1467	9.7063	10.7317
6.7677	6.2023	6.5220	5.2977	5.5153	5.5113	5.8250	5.8603	5.3147
8.4337	7.8847	8.0257	8.5480	7.9470	7.5483	7.5723	7.5180	7.6463
39.7987	38.3593	39.3240	38.8087	40.4453	42.2583	42.9520	41.8077	40.5397
29.4557	29.9710	31.0857	29.4897	27.1610	26.3050	25.4850	26.7283	27.0177
14.7180	15.6330	14.7540	14.6060	13.7820	13.5110	13.3060	13.3253	13.8467
12.7550	12.6140	11.7460	11.7857	10.8923	10.5370	10.3303	10.3127	10.7353
6.5817	6.3713	7.1693	5.2427	6.3137	6.0440	6.6943	5.0650	5.9327
10.9583	12.3737	11.8353	12.8077	12.1400	12.2650	12.4577	12.4223	12.2933
14.9830	14.6523	13.9067	12.8243	12.4223	12.5477	12.4530	12.4290	12.5030
17.3947	16.1237	18.6603	16.8677	16.5263	15.9433	15.8213	15.6023	15.9413
10.6950	10.5820	10.5090	9.9200	9.0967	9.0110	8.8253	9.0743	9.0657
30.3030	31.7303	30.3013	29.4733	30.1247	29.2250	29.0030	28.8770	28.5377
35.2807	35.9390	34.7920	36.2627	33.6737	31.4690	31.6997	33.0943	35.4297
15.7367	15.3147	15.1660	15.3473	14.2250	14.5413	14.3473	14.0517	14.1020
38.5500	38.1220	37.0810	37.1343	37.7507	37.9527	37.8640	37.5713	36.7943
12.1173	12.6137	12.2653	11.8120	11.4053	10.4427	10.8240	11.4897	11.3283
6.2903	6.4717	6.8090	7.4103	6.2527	6.1857	6.1960	6.7623	7.0517
23.6893	22.9557	22.8687	21.4947	23.2723	23.8623	23.7143	22.0690	21.7470
10.6107	10.2863	9.9987	10.4583	9.3770	9.7877	9.7467	9.6457	10.0960
11.4430	12.2830	12.7837	11.8403	11.0393	10.5290	10.3927	10.5510	11.1513
10.4040	10.4430	11.2923	11.1997	10.9337	9.9027	10.0913	10.8280	10.9323
25.6067	26.1527	25.3127	25.1990	24.0267	23.8613	23.3297	23.4280	24.0247
33.5763	32.5520	32.6787	31.8330	32.4607	32.0583	31.9537	30.5553	31.3723
12.8970	12.6370	12.5503	12.1360	11.7027	11.3370	10.9320	11.3510	11.4370
18.1050	18.3790	18.2363	17.9607	16.6583	16.6813	16.6077	16.6650	16.4193
2.8653	2.6297	2.5427	2.4330	2.6847	2.7210	2.6930	2.8580	2.6153
11.5070	11.9807	11.7707	11.9113	10.7413	9.9680	10.2427	10.5483	11.1433

16.1693	16.9410	15.7827	16.3913	14.3047	13.0477	12.8497	13.7520	14.5317
17.5617	17.6950	17.6037	16.6837	15.8623	15.4973	14.8257	15.3410	15.5350
13.1863	13.3023	13.2503	13.3757	13.5387	14.0257	14.2967	13.9267	12.9997
15.8133	16.9283	15.7087	15.1570	14.5187	13.9727	13.9943	13.7537	13.6943
10.5247	10.3263	11.2830	10.1357	11.2953	12.3380	12.5430	12.9903	12.3223
26.3370	28.0647	27.8737	25.7293	25.4287	24.7410	24.6153	24.5280	25.6837
32.2590	30.6813	30.3960	29.9447	29.9660	29.4180	29.1733	29.4263	28.3303
11.9843	13.1443	12.6497	11.4473	12.0497	12.1720	11.9757	11.7383	12.1067
18.2920	18.0623	19.9173	18.5630	18.1163	18.1130	17.9013	17.4260	17.6910
13.2230	13.6060	12.8933	12.7757	11.6647	10.3127	9.6920	10.0870	10.4383
41.4107	42.5193	41.5377	39.8363	41.0380	39.7183	39.8710	39.8410	39.6867
37.3127	39.2110	37.9197	37.3200	35.4627	32.3273	31.4337	31.8907	34.6250
32.5757	32.9283	31.6760	31.4193	31.5717	31.4920	32.0370	31.1247	29.5593
33.4263	33.4373	34.1847	32.8363	32.3270	32.7220	33.1740	32.9463	32.8250
11.8750	12.1340	12.2763	11.7353	10.8603	10.0337	10.3413	10.8113	11.0220
15.4663	15.9940	14.8740	15.5750	14.1647	13.6603	13.5610	13.6593	13.6887
18.4017	18.1120	18.9647	17.2910	17.3283	16.9350	16.5437	16.4287	16.2383
15.9953	16.1387	15.5003	15.5480	15.7327	15.9807	15.8547	15.5640	15.1653
11.9403	12.3843	11.6347	11.2857	10.9210	10.1077	10.0073	10.3577	11.0183
19.7280	20.3703	19.5600	18.8380	18.3487	18.6180	18.3597	17.7630	18.6143
14.5143	15.0163	14.4603	14.2657	13.6553	12.9860	12.9280	13.1853	13.8977
5.6013	5.2680	5.4583	5.3400	5.0370	5.1360	4.9487	5.5323	5.1927
12.1090	13.7847	13.8420	12.8733	12.2493	12.5707	13.1293	12.6017	12.7847
8.5537	9.0440	8.6233	9.1473	8.7337	8.5977	8.8740	8.9847	8.7760
20.1047	21.5910	20.0743	20.6913	19.3423	18.6920	18.7913	19.8633	20.4687
18.7147	19.1603	18.4510	18.5497	17.7000	17.5677	17.0927	16.9927	16.9447
13.3137	13.1197	13.0770	12.7753	12.5707	12.6913	12.6370	12.8097	12.1653
12.8257	13.3350	12.9407	11.9820	11.9713	12.0563	12.0130	12.0443	11.9307
12.9703	13.0427	12.9813	12.8517	11.7513	10.9177	10.7423	11.6697	11.6030
16.7370	15.0387	15.0387	14.1960	13.1093	12.3580	12.3593	12.3777	14.0773
17.9787	18.5773	17.7200	17.4990	17.3513	17.0667	17.1150	16.4397	16.6357

432.02nm	435.11nm	438nm	441.09nm	444.17nm	447.05nm	450.13nm	453nm	456.07nm
29.4267	28.5660	28.4967	28.7283	27.6863	28.1073	26.7283	26.6583	25.6467
22.3573	21.5973	21.5227	21.6200	21.1520	20.6730	20.6633	20.6177	20.8513
13.1387	12.9283	13.0947	13.1243	12.8033	12.8753	12.8367	12.4107	12.6673
30.2150	29.9933	29.6640	28.8940	28.7220	28.3250	28.7347	27.3547	27.6290
22.7833	22.8683	22.3683	21.8243	21.8070	21.0567	21.7380	21.1520	21.8750
9.9137	9.7837	9.5780	9.3797	9.1177	8.6020	9.4030	10.0470	9.5327
19.0583	18.6343	19.1903	17.8763	17.5640	18.0617	17.1017	17.9677	15.3607
22.6317	21.1140	21.4170	21.6053	21.3513	20.2803	20.3527	19.4090	20.2573
13.5383	12.8753	12.5177	12.5743	12.7873	12.0380	12.5533	12.7030	12.7907
11.7403	11.5213	11.3527	11.7033	11.2230	11.0613	11.1890	11.2820	11.2187
17.2277	16.7610	17.8787	17.4250	18.2163	18.0340	17.8610	17.2367	17.3677
11.9147	11.5750	11.9500	12.2237	12.1323	12.4527	11.5413	11.6253	10.9673
32.2327	32.6823	31.6017	30.4417	30.9090	29.5203	30.9253	31.1463	30.3533
23.4990	23.4927	23.4457	24.3670	23.6383	24.8450	22.7403	22.5937	21.4617
12.8017	12.9350	12.1097	12.7273	12.4743	12.6277	12.2313	11.5880	11.7230
23.8520	25.5810	23.8370	23.0913	23.0610	22.9040	22.1877	22.4173	21.2267
32.2717	30.5960	31.3607	31.2180	31.1363	32.9743	29.5953	29.9607	27.4927
12.1323	11.5287	11.5490	11.0010	11.4013	11.3117	11.0317	10.7677	10.5157
12.5457	13.3923	12.8067	12.3710	12.2787	12.2670	12.6217	12.3980	12.8343
24.8200	24.8590	24.9397	25.4340	25.2063	24.4843	23.5803	22.5500	22.3890
18.4623	18.2917	17.2307	17.4337	16.4023	16.4300	16.4620	16.2700	15.4963
14.5057	14.0727	13.4407	13.2877	12.8420	12.7470	13.3037	13.0893	13.1497
26.8843	26.2083	25.7597	26.3077	25.6693	24.5927	24.9787	24.6660	24.8407
18.6893	18.9753	18.0703	17.7667	17.1477	17.2330	16.7360	17.1743	17.0537
29.8547	30.2097	30.8420	31.1847	30.7367	31.1980	29.0733	29.4410	27.7940
24.0723	23.9673	23.9763	24.9447	22.4277	22.8950	23.3767	22.3890	21.6470
23.8403	23.1313	23.2280	22.8423	21.5580	22.3507	20.0053	20.9113	20.9463
13.6583	14.0693	13.4110	13.4140	13.4527	13.2183	13.1850	12.5637	12.7293
14.1203	13.4763	13.9390	12.2890	12.7473	12.3450	12.3923	12.4830	12.3587
30.1453	30.5593	30.0023	30.2037	28.5417	29.5600	27.5027	26.6597	24.9843

14.0130	14.1407	13.4450	13.6577	13.6817	13.7890	14.1743	13.5200	12.8233
10.3387	10.1693	10.0810	10.4223	9.8440	10.2913	10.1813	10.2620	10.2427
8.8180	8.8217	9.0177	8.9440	8.3037	8.7453	8.6307	8.0860	8.0877
15.2893	15.2483	15.4170	15.1287	15.2807	14.9380	14.6157	14.2927	14.8243
14.5613	14.0447	14.2027	13.7053	13.7797	13.5093	13.4453	13.0833	13.1687
9.9953	10.6573	9.8390	9.4080	9.6840	9.0263	9.6303	8.9457	11.0007
5.2693	5.1623	5.1717	5.4163	4.5980	4.9207	4.3910	4.4350	4.9227
7.6023	7.9747	7.4953	7.8367	7.5017	7.3073	7.7090	7.7627	6.8070
41.6520	41.2003	41.4683	42.0380	41.1403	42.3540	40.3410	40.1550	39.3927
26.3007	26.9087	25.6827	24.6990	23.7607	23.3923	23.2963	24.4940	24.1407
14.0473	13.5567	13.6433	13.0340	13.2837	13.4553	13.0983	12.7827	13.5653
10.5143	10.7200	10.5653	9.8513	9.3570	9.6233	10.1123	9.3590	9.3927
5.8853	5.7907	5.5720	5.6787	5.8550	6.1780	6.6640	6.2857	6.9450
12.0633	11.0750	11.0950	10.2533	10.6430	10.9780	10.2370	9.7600	10.4997
12.0037	11.3517	12.0263	11.7360	11.8257	11.4597	11.3967	11.4617	11.4103
15.8100	15.4493	14.9803	14.4123	15.3907	14.8543	15.2473	14.6037	15.0580
8.5127	8.6200	8.1363	7.7283	7.8553	7.9070	7.7833	7.8650	6.9007
27.6467	28.0937	27.7593	27.3010	27.4080	27.1843	25.8083	26.4387	26.4123
33.7450	34.8710	33.4077	33.0477	33.1403	31.2883	34.3477	33.4393	34.3123
13.8513	13.8103	13.9177	13.6740	13.2933	13.1207	12.6870	12.8747	12.8957
37.2130	37.0820	37.2320	37.1827	36.8893	37.7930	36.5550	36.9883	35.9170
11.4400	11.2093	10.5487	10.5617	10.7763	10.2720	11.0830	10.5803	10.9467
6.5493	7.0463	6.8287	6.6447	6.5707	6.5963	6.7210	6.7957	6.6850
21.8980	22.1343	22.2333	22.4083	22.3393	22.9697	20.5450	20.5070	18.8820
10.1423	9.7850	9.7193	9.4133	9.8560	9.3563	9.8153	9.1713	9.5883
10.6900	11.1787	10.8550	10.2847	10.4063	10.3327	10.5853	10.6503	10.7200
11.0367	11.2337	11.2587	11.0923	11.2973	10.8867	11.2340	10.9653	11.0063
23.8500	23.3940	23.4327	23.0183	22.1187	22.1847	21.6950	21.2463	20.7120
30.5687	30.9690	30.2723	30.7057	30.1300	30.7657	29.9990	28.7263	29.6960
11.0730	11.1257	10.8100	10.4370	10.7013	10.4047	10.2787	10.2790	10.4087
16.7700	16.4803	16.1693	15.9613	15.7513	15.8750	15.9840	15.3973	15.2357
2.8660	2.4560	2.8243	2.8773	2.8787	3.1030	2.6077	2.6707	2.9007
10.8757	10.7270	10.4813	9.8900	10.5540	9.8957	10.4220	10.3793	10.4013

14.0490	14.2970	13.4983	12.9747	12.9410	12.4980	14.0267	13.3543	14.3670
14.9737	15.2487	14.7823	14.1230	13.9627	13.8567	13.8597	13.6603	13.7203
13.7333	13.3603	13.6297	13.9717	13.5750	14.1330	13.0373	13.2533	12.9670
13.5123	13.5100	12.8343	12.8310	12.6557	12.5700	12.5457	12.2673	11.6583
12.1857	12.4787	12.4937	13.1180	12.9867	13.4127	13.0917	12.7653	12.9020
24.0537	23.8297	24.3003	23.7663	23.6330	23.0093	22.4657	22.8440	23.0657
27.5633	28.1487	28.2637	27.5627	27.2897	27.3323	25.9800	26.0647	25.4087
11.7717	11.8873	12.1283	11.9887	11.6883	11.8637	11.6383	11.6847	11.3563
17.2737	17.2093	17.1097	16.5057	16.7793	16.2640	16.3050	16.5563	16.3957
10.1673	10.2280	9.8803	8.9797	9.2690	8.3850	8.9693	8.9550	8.9010
38.7033	38.8593	38.3867	38.3557	37.7287	37.5897	36.3637	36.2920	36.7470
33.4517	31.8677	30.9910	30.5183	30.5877	28.9017	31.3057	29.7833	30.7677
29.7790	29.3497	28.6073	28.2960	27.6607	28.0263	26.4033	26.4847	25.3423
33.2480	33.4800	31.5480	32.1933	31.9997	31.8963	30.6623	30.5200	28.9957
11.0237	10.9347	10.6477	10.2380	10.6387	9.9317	10.7210	10.8620	10.9983
13.1403	13.2173	12.7563	12.3780	12.0560	11.6777	12.0083	11.1627	11.2877
15.8903	15.0293	14.8587	14.1163	14.4887	14.6327	13.6030	13.4913	13.1640
15.5637	15.6697	15.5123	15.2240	14.7673	15.0183	14.3813	14.1140	14.0630
10.2393	10.6410	9.7493	9.9723	10.0247	9.4917	9.8837	9.8797	9.9193
17.6833	17.2467	16.7383	17.5000	17.0993	16.4053	16.5560	15.8667	15.4140
13.7347	13.7137	12.9067	13.1000	12.7557	12.8293	12.7727	12.7360	12.7650
5.4377	5.4203	5.6430	5.4640	5.2827	5.6263	5.9163	5.9957	5.3027
12.3977	13.1497	13.3723	12.7190	12.8070	12.3163	11.7597	12.0317	12.1477
9.2540	9.3910	8.7030	9.0350	9.2747	8.9200	8.7473	8.7957	9.1087
19.8337	19.9517	19.2597	18.8280	18.9133	18.0550	19.6380	18.3507	19.0737
16.9557	16.4380	16.3307	16.1217	16.0127	15.7013	15.5763	15.3033	14.7670
12.4990	12.2010	12.3790	12.1613	12.1913	12.1233	11.9750	11.7857	11.4813
11.9017	11.5993	11.8077	11.9567	11.3590	11.5790	11.2230	11.1727	11.2557
11.3813	11.6053	10.9040	10.7227	10.6197	10.6507	11.2627	11.1293	11.2047
12.7367	14.3220	13.5003	12.4210	12.4043	11.6860	13.3090	12.8980	12.7363
16.8770	16.4510	16.6370	16.3213	15.8950	15.8697	15.6633	15.7553	15.4177

459.14nm	462nm	465.07nm	468.13nm	471.19nm	474.05nm	477.1nm	480.15nm	483nm
27.2070	26.4597	26.0427	25.3270	24.5237	25.2670	24.9697	24.3757	26.7677
20.7210	20.3600	19.7477	19.5773	20.1227	20.0273	19.1867	19.2743	18.6387
13.4507	12.9723	12.9510	12.4363	12.7367	12.3210	12.3060	12.7067	12.9263
27.0583	27.5917	26.3807	26.2593	26.3897	27.0147	25.1640	25.0140	24.0137
20.4743	21.4550	21.3330	20.9627	20.8373	21.1690	20.1377	20.0193	18.4423
8.5507	9.3480	9.3710	8.7773	8.7863	8.8170	8.8290	8.6393	7.1827
17.0640	15.6287	16.0407	14.8323	15.7047	15.1287	15.3857	14.6513	17.5263
19.9800	19.5763	20.4710	19.8627	19.7800	17.9453	19.7423	19.0890	19.5563
12.5850	11.6273	11.4960	11.6013	11.6063	11.6533	11.8847	11.5130	11.6013
10.9830	10.7260	10.7367	11.0600	10.8340	10.1563	10.5823	10.7840	10.7443
18.1737	18.0020	17.5027	18.0257	17.7110	16.3513	16.5660	17.5050	18.6977
12.4300	11.5313	10.5563	12.0337	10.9190	9.9693	11.1597	11.4347	12.4983
27.6487	29.8417	29.9667	29.5973	29.3433	29.2570	29.8123	28.1620	23.5243
23.1330	22.9250	20.8607	21.5720	21.3683	20.6613	20.8560	21.9707	24.1397
12.1520	11.6330	11.4223	11.3617	11.3480	10.4660	10.9513	10.7587	12.1887
21.4020	20.8430	21.4007	20.4223	21.2140	20.1200	19.9260	19.7267	18.9410
31.4017	28.9053	27.3387	26.1140	26.4910	26.3533	27.4100	28.3497	32.9883
10.7323	10.2770	10.3660	9.6983	9.5303	9.8240	9.8557	10.2077	10.8847
11.5577	11.8043	12.4190	11.4873	11.8577	12.2657	11.6700	11.3807	10.1567
24.1970	22.4783	21.4037	20.8170	20.6700	20.7867	20.8520	21.0423	23.9390
15.7503	15.4600	15.2973	15.5743	15.2420	14.1973	14.3903	13.9053	13.9823
12.4227	13.1930	13.1433	13.3517	12.9597	13.3343	12.5607	12.1557	10.2737
23.9437	24.0447	24.6550	23.9777	23.0630	23.3573	22.6733	22.4140	21.4353
16.2170	15.8653	16.3457	15.8727	15.6183	15.7163	16.1257	15.8027	15.3147
30.1933	28.2990	26.2620	26.9433	26.5420	27.4177	27.7400	27.7313	32.1080
21.8773	21.4720	21.1977	20.9477	19.9750	20.5097	19.8337	20.5930	20.6993
21.1287	20.4047	18.6130	19.3093	18.2717	18.2177	18.5697	18.4897	20.6620
12.3570	11.7420	12.1593	12.0970	12.2077	12.1763	12.3720	12.0220	11.9070
11.1753	11.5040	10.3570	11.6493	10.9633	10.7960	11.7343	10.9840	9.9043
27.3130	25.7180	24.7320	24.1123	24.8123	23.1657	23.6653	24.2137	27.7443

13.1737	12.7650	12.6780	12.2847	12.8117	12.5967	12.5150	12.6823	13.6153
10.1717	10.3157	10.2227	10.4133	9.7850	9.9820	9.6860	9.8687	9.3327
8.4453	8.1280	7.6563	7.9603	7.8620	7.4963	7.5310	7.5303	8.4870
13.9927	14.2953	14.4360	13.8917	13.8247	14.0173	13.9120	13.6603	13.4280
13.0523	13.2073	13.0483	12.7740	13.2523	12.7523	12.6423	12.6150	12.6707
9.0537	9.1710	10.4143	9.9863	8.5013	10.4460	9.9927	9.2303	7.5720
4.5390	3.9430	4.3980	4.3357	4.0410	4.2340	3.6247	4.0743	4.7587
7.4880	7.7810	7.5160	6.8877	6.9790	7.0987	7.0270	7.0680	6.9890
41.1080	39.7213	38.6427	38.6233	37.9233	38.5130	38.3773	39.1277	42.4063
22.2297	23.8407	23.7783	23.1877	23.8167	23.3723	22.2403	22.1440	18.5107
12.7107	12.7927	13.3060	13.2087	12.6393	12.2707	12.7410	12.6477	12.0603
8.9047	8.7863	9.3807	8.6627	8.8117	8.6430	8.9040	8.1667	7.9343
6.4923	6.0573	5.8697	7.4140	4.8317	6.4173	6.7067	6.7707	6.0553
10.7697	10.2147	9.8423	9.5537	8.9950	9.5237	8.7423	9.2447	9.7197
11.4027	11.1120	10.4883	10.5610	10.0667	9.9750	10.2347	10.6273	10.5210
14.0890	14.6433	15.6743	14.7070	14.5540	14.8583	13.7613	14.0530	12.3240
7.1937	7.3837	6.9283	7.2660	6.5580	6.8190	6.8847	6.8507	6.5093
25.6657	24.7037	25.3010	24.4243	24.5767	24.6503	24.3500	24.7130	24.3707
31.2107	33.9047	33.6127	33.7640	33.3350	33.1040	32.5110	31.7273	27.9973
12.7730	13.0363	12.8533	12.2667	12.3013	12.4773	12.1880	12.3227	12.5173
37.1490	36.8737	36.1987	35.8330	36.6223	35.2153	35.9153	36.2380	37.5543
9.7423	10.5673	10.3333	9.7357	9.8530	10.1007	9.9167	9.8540	8.8953
6.4350	6.4973	6.7487	6.6420	6.6070	6.7807	6.6823	6.5010	6.2893
21.0840	19.3850	18.6913	19.4763	18.9353	16.9687	18.3140	18.8923	21.9533
9.4707	9.0880	9.9893	9.9233	9.5330	9.4370	9.3047	9.5753	9.2607
9.8357	10.4063	10.1157	10.3823	10.1393	10.3150	9.9123	10.1020	8.5467
10.8750	11.4367	11.8563	11.4810	11.6780	12.2583	12.0977	12.5087	10.8017
21.6090	20.9280	21.2430	19.9383	20.4323	20.7673	19.6053	20.3173	19.1770
29.2803	28.7060	28.2333	28.6957	27.2550	26.5037	26.9347	27.7887	28.4837
9.8277	10.2233	9.8053	10.2357	9.7687	9.4907	9.4160	9.7207	9.1483
15.1867	15.4240	15.2380	15.0600	15.3800	15.2683	14.9253	14.8780	15.4383
2.9963	2.8023	2.9753	3.2387	3.2163	3.0340	2.9363	3.2033	3.3093
9.5883	10.1223	10.1357	10.1970	10.1530	10.4067	10.0483	9.6240	8.0020

12.5117	13.9380	13.5847	13.6947	13.8517	13.7910	13.2833	13.0687	10.2000
13.3620	13.8087	13.5487	13.3610	13.1280	12.8233	12.8817	12.9387	12.0757
14.0193	13.0347	12.2927	12.5893	12.8200	12.4513	12.9173	12.7133	14.9797
11.6397	11.5467	12.1783	11.3970	11.3930	11.2067	11.2313	10.7920	10.2093
14.2533	12.9920	13.3577	12.8987	12.5593	12.1877	12.9957	13.8263	16.0433
21.6167	22.0003	22.5800	21.9703	21.1890	21.2233	21.6167	20.7017	18.9923
26.4403	26.1360	25.2690	24.1883	24.3683	24.2067	24.4753	24.1737	25.0180
11.5200	11.5030	11.4820	11.4023	10.8533	10.5187	11.1753	10.4443	11.0707
15.5377	15.2307	15.3807	15.3300	14.4257	14.8530	14.6627	14.7633	14.6017
8.1520	8.4887	8.6563	8.9060	8.2537	8.9783	8.1003	7.8700	6.2600
36.5797	35.9910	35.2130	34.3100	34.0850	33.8013	33.8390	33.9167	34.8600
28.3077	29.3233	30.5750	29.7177	30.2590	29.9133	28.9893	27.3643	22.9607
26.1257	24.1707	23.4643	23.7060	23.8100	23.5833	22.5400	23.7083	25.5480
30.4523	30.4937	29.8173	29.7870	28.1080	28.2887	28.9320	28.4813	29.6753
9.7933	10.1380	10.6563	10.5340	10.5323	10.3413	10.0467	10.2200	8.6727
11.0003	10.7503	11.4583	10.4503	10.4773	11.4327	10.2923	10.6180	10.2177
12.8527	12.9533	11.4863	11.5587	11.5870	12.0777	11.9977	12.0453	12.7897
14.6350	13.4867	13.8223	13.7100	13.2203	13.3370	13.7087	13.6393	14.8790
9.2570	9.8080	9.9487	9.2400	9.4670	9.7887	9.2707	9.3190	8.1313
16.0400	15.3687	14.5797	14.7837	14.6763	14.3153	14.4877	14.5877	15.5927
12.4827	12.8577	12.7067	12.3897	12.1903	12.2957	12.2880	11.8310	11.3860
5.4193	5.3337	5.5083	5.6843	5.6073	5.7963	5.7290	5.9157	6.1130
12.0333	12.1690	12.1273	11.8967	11.7510	11.3417	11.0407	12.0077	11.7683
9.1087	9.0710	8.9043	8.7353	8.9157	9.1150	9.0643	8.9057	8.6177
18.1673	18.9193	19.1453	18.2173	18.6307	18.2067	18.7480	17.8603	15.3237
14.7523	14.5470	14.5807	14.4267	14.2027	13.7247	14.2070	13.9097	14.0420
11.5027	11.5040	11.1927	10.8960	11.5637	11.1623	11.0840	10.9360	11.3810
11.2070	11.1637	11.2983	11.1347	10.3177	10.7280	10.7507	11.1513	11.1847
10.2170	11.0257	10.6113	11.1707	11.0563	10.5883	10.4263	10.1133	8.9537
11.2193	11.7307	12.1577	11.9670	12.0817	11.7777	11.4240	11.7070	9.1980
15.2383	15.2553	14.9010	14.9467	14.4580	14.6960	14.7707	14.5950	14.7940

486.04nm	489.09nm	492.13nm	495.16nm	498nm	501.03nm	504.06nm	507.08nm	510.1nm
26.6590	26.1490	26.4843	25.4100	25.0750	23.6757	24.0973	24.9077	24.7100
18.9733	18.8767	19.2147	19.2800	19.3683	19.3407	19.4097	19.8523	19.2533
12.9913	13.0950	13.2447	12.7573	13.4520	13.0757	12.9097	13.4670	13.3697
24.1440	25.3957	24.7550	25.2347	24.3247	25.2577	25.8623	26.1610	25.6963
18.6077	19.0220	18.6817	19.9333	19.4223	19.9890	19.7140	20.0273	19.8880
7.5360	7.7103	7.5787	8.0530	8.3950	8.1453	8.3683	7.9103	8.0640
17.1540	17.1403	17.2520	16.1650	15.5243	15.6157	15.3080	15.1673	15.8633
19.8353	19.5013	19.7230	19.7130	20.1783	18.8393	19.3627	18.4940	18.9590
11.8023	11.1133	11.5830	11.3263	11.2667	11.2467	11.0323	10.9753	10.6590
10.8877	10.8547	10.6427	10.5727	10.4323	10.2270	10.1913	10.2557	10.4710
18.5960	18.7247	18.2407	17.0397	17.8200	16.9523	16.7787	17.4440	16.7223
12.5537	12.6840	12.7920	12.3003	11.7133	11.3893	12.4527	11.5520	11.9503
24.3770	25.2643	25.4390	27.1897	26.6627	27.4860	27.5043	27.1780	27.3443
23.6223	23.0083	23.7293	21.4490	22.2363	21.0480	20.7793	20.9603	21.8337
11.7080	11.5947	11.6193	11.3317	11.3173	10.7303	10.8273	10.7373	10.7657
19.1860	19.7380	20.0017	19.9967	20.5570	19.0077	20.2960	20.3750	20.5587
32.0770	31.1630	32.2213	28.6107	29.7213	28.1440	28.3910	28.8100	28.8780
10.4113	10.4477	10.4937	10.1837	9.8847	9.8417	9.9300	9.7590	9.9047
10.4363	10.3563	10.0963	10.7800	10.8757	10.7493	11.1990	10.8307	10.6513
23.4097	22.9340	23.2290	20.6317	21.7760	21.7673	21.5913	21.0460	22.1450
14.4620	14.4757	14.4357	14.3863	14.6123	13.7967	14.8687	14.6823	14.9463
10.6380	11.1213	10.8587	12.1337	11.6997	12.5740	12.6723	11.9203	12.0977
21.8370	22.8163	22.0067	22.5720	22.4923	23.7923	23.1477	22.4800	23.1647
15.4177	15.1450	15.4410	15.3473	15.6583	15.8647	16.0467	16.0463	15.6890
31.6267	31.0110	31.4140	27.7777	29.6353	29.2983	28.6570	29.0087	29.5033
21.0640	20.6980	20.3263	19.9323	20.9400	20.2483	20.4657	20.1007	21.4377
20.7123	20.2227	19.9703	19.1867	19.3177	19.4343	19.5747	19.0817	19.9573
11.9347	11.8997	11.7167	12.1627	11.5367	11.6920	11.9073	11.8900	11.7940
10.0753	10.2260	10.0600	11.8363	10.3847	10.5923	11.9800	10.5727	11.4187
26.8980	26.6787	26.1917	25.3957	25.2383	23.8723	24.4517	23.3703	24.8273

13.4413	13.4453	13.4683	13.0367	12.9973	12.8053	12.5793	13.2353	12.9623
9.6137	9.6423	9.8467	10.3933	10.3370	10.4177	10.8633	10.4430	10.0743
8.5570	8.2317	8.6133	8.2393	8.6047	8.7330	8.3403	8.1460	8.4230
13.6210	14.0043	13.8477	13.5910	13.4390	14.0550	14.0380	13.8257	14.0747
12.5483	12.6590	12.8653	12.9033	12.9633	13.1357	13.3360	13.4770	13.9077
8.2513	8.2153	8.4017	9.2737	8.8953	9.3860	9.2847	9.0513	9.5640
4.6050	4.2390	4.8853	4.4780	4.3233	4.2860	4.2397	4.5303	4.4523
7.1557	7.2297	7.1457	6.7143	7.3347	7.0473	7.4007	7.4613	7.4033
42.4470	42.6443	41.8907	39.6683	40.5673	38.5603	39.3303	38.7653	39.0547
19.3630	19.6833	20.0910	21.1090	21.2213	22.2910	22.0920	21.6767	21.1783
12.4530	12.3810	12.2430	12.6060	12.4830	12.3717	12.6617	12.6377	12.3493
8.0350	7.8393	7.9153	8.0873	8.1483	8.6693	8.0440	7.8743	7.9133
5.8333	5.8783	6.2743	6.4137	6.5383	6.8823	6.8233	6.5133	7.6037
9.4837	10.0030	9.8050	8.8103	9.9177	9.6863	10.0577	10.5150	11.2327
10.5843	10.4550	10.7630	10.4580	10.3993	9.5780	10.3623	10.4527	10.6877
13.0513	13.2817	12.8620	13.9483	14.5463	14.0777	14.8647	13.6733	14.2313
6.7690	6.7963	6.5937	6.5233	6.2553	6.5827	6.3690	6.9590	6.6463
24.5863	24.8917	24.2790	23.4743	23.7660	23.5840	23.7007	23.7543	23.3203
28.6007	29.5017	28.7133	30.5863	30.1277	31.6250	31.3480	30.9927	30.7333
12.5590	12.4983	12.4697	12.4247	12.4160	12.3777	12.4977	12.5127	12.8253
37.6347	37.8060	38.2123	37.3680	37.4820	37.5093	38.2303	37.8100	37.9247
9.1380	9.1767	9.3310	9.4643	9.2607	9.2447	9.2117	9.3573	9.5443
6.0860	6.5247	6.6613	6.8927	6.8587	7.0753	6.8770	6.7513	6.9047
21.8250	21.1230	21.2110	19.0393	19.5007	18.0087	18.4937	19.0763	18.9990
9.2193	9.4293	9.4797	9.8497	9.7663	9.7723	9.6900	10.0533	10.2493
8.9503	9.1730	9.0900	10.1680	9.6893	10.0960	10.1363	9.9900	9.8857
10.8907	11.6750	11.2757	12.5363	12.4110	12.9807	12.9310	12.7390	12.6470
19.7197	19.7887	19.4370	19.4430	19.9630	19.0103	19.0040	18.9767	18.7263
28.0947	27.9947	27.9230	26.7433	27.2120	26.6780	26.2013	26.5830	25.9630
9.3467	9.6023	9.4893	9.2353	9.3207	8.9983	8.9913	9.1323	9.1423
15.1760	15.2450	15.2093	15.0450	15.1090	15.5990	15.6723	15.8037	16.2677
3.2327	3.2667	3.5587	3.3387	3.6070	3.4970	3.6830	3.4257	3.6703
8.5157	8.6493	8.5243	9.3867	9.4470	9.4463	9.7427	9.3823	9.2280

11.0070	11.3900	11.2160	12.7417	12.3377	13.2100	12.9043	13.4067	12.6793
12.4557	12.6007	12.7613	12.9657	12.9920	13.5253	13.7297	13.8793	13.4303
14.8903	14.3850	14.4093	12.7280	13.3193	12.5457	12.7767	12.6957	13.3770
10.5663	10.2480	10.4383	10.6870	10.8050	10.3610	10.1307	10.3880	10.7073
15.7843	15.5403	15.3590	13.8960	14.7053	13.4533	14.0673	13.8690	14.2133
19.8057	19.9803	19.3627	21.1587	20.3107	20.5967	20.9723	20.8967	20.3307
25.4480	25.5220	24.7213	24.0980	24.6313	23.4467	23.7787	23.4020	23.6037
10.7327	10.6113	10.6953	10.6050	10.4690	9.8217	10.1290	10.1407	9.9877
14.8923	14.5250	14.9050	14.6657	15.4953	15.3320	14.8847	16.0877	15.8070
6.7557	7.1687	7.0450	7.9303	7.9063	8.5673	8.9327	8.9000	9.2577
34.1917	33.9617	34.4453	33.6483	33.9903	32.5990	32.9293	33.4363	33.4870
23.8137	25.3013	24.5683	27.6413	26.6570	27.7543	27.1517	27.1367	26.7563
25.1260	25.0550	24.6007	23.2423	23.0470	23.0917	23.4080	22.9670	24.1940
29.7590	29.7427	29.9570	29.0670	28.6980	29.2610	29.2010	29.6857	30.1773
8.7397	9.3257	9.0590	9.8223	9.5187	9.9707	10.4573	10.0403	10.2967
10.4397	10.3053	10.7123	10.9603	10.7970	11.0040	10.6960	11.2183	11.4503
12.6273	12.8340	12.9210	12.5003	12.4047	12.2853	11.3317	12.5703	12.6620
14.9240	14.7117	14.9890	14.1220	13.9547	13.8070	13.9140	14.5013	14.5257
8.2713	8.6090	8.5743	9.0710	9.2077	9.3533	9.1597	8.8870	8.8153
15.3277	15.1887	15.1273	14.8320	14.9283	15.4330	14.9857	14.4877	15.0370
11.6953	11.7927	11.7587	11.9980	12.1613	12.6010	12.1443	12.0630	12.1170
6.3160	6.0817	6.3447	6.2973	6.7307	6.3030	6.6010	6.7570	6.9223
12.1867	12.6987	12.9017	12.5843	12.8040	12.6400	12.8007	13.1330	13.0977
8.9790	9.0850	8.8563	8.8130	9.1753	8.7243	8.8827	8.9053	9.0250
16.3243	16.7277	16.8643	17.8247	18.5177	18.3423	17.9900	18.5773	18.9930
14.1363	14.0890	14.1017	13.6987	13.6253	13.8267	13.7143	13.9767	13.9993
11.4887	11.3343	11.1733	11.0010	10.9577	10.7100	11.0210	10.6900	10.9890
11.3070	11.3563	11.2977	11.3217	11.1333	10.9697	11.0827	11.7620	11.4587
9.3777	9.4803	9.3190	9.9593	10.1023	10.7090	10.6017	10.0940	10.1300
9.8200	10.3407	10.2007	11.3910	11.6963	12.3163	11.4097	11.5613	11.4970
14.7743	14.9723	14.9940	15.1033	14.7770	14.9680	15.0483	15.0370	15.6577

513.12nm	516.14nm	519.16nm	522.17nm	525.17nm	528.18nm	531.18nm	534.18nm	537.18nm
24.3510	24.4833	25.2623	24.1977	25.2283	26.7700	25.8603	26.4043	25.4013
19.0860	19.4537	19.7477	19.1860	19.5133	18.7603	20.1773	19.4843	19.9017
13.5287	13.5887	13.7140	13.6927	14.1107	14.3097	14.6210	14.5350	14.6440
25.8357	25.4423	25.7010	26.0950	26.3137	25.6647	26.7150	26.8460	27.4810
20.2743	19.9373	19.6373	20.3903	19.9613	19.5750	20.1917	20.2843	20.9897
8.2690	7.9813	7.6397	7.7767	7.5243	6.8440	7.5577	7.6753	7.6940
16.5763	16.5557	17.3490	17.3583	18.3893	20.0090	19.5010	19.6427	19.1197
19.1690	19.1370	19.9763	19.2160	20.1663	21.1223	20.3450	21.1633	21.0943
11.4003	10.4650	11.3097	11.2747	11.3270	11.0643	11.1887	11.2417	11.8030
10.6193	10.0600	10.5120	10.3860	10.4397	10.8173	10.5437	10.6250	10.4263
17.7650	17.1800	17.7803	17.0607	17.8047	18.1337	18.2423	17.5150	18.0127
11.4627	12.1017	11.5900	11.3420	12.5817	12.5100	12.5970	12.0817	12.0793
28.0213	27.7047	26.3180	27.3837	25.8393	23.7550	25.3673	25.2353	26.6700
20.6597	21.3843	21.6843	20.6530	21.9327	23.0270	21.9687	21.9327	20.9607
10.8847	10.8697	10.9047	11.1263	11.3580	11.8463	11.6353	11.5283	11.0510
20.8580	20.1657	20.9347	21.2837	21.0323	20.6610	21.5873	22.0130	21.9537
29.0793	28.2763	30.9403	29.4193	31.5517	34.7913	33.3923	33.2990	32.8567
9.9610	9.7837	10.5533	10.3447	10.7970	11.1960	10.7403	10.9010	10.6697
11.0637	10.6113	10.9267	10.9380	10.1747	10.0287	10.4230	10.5860	10.6627
21.7483	21.3003	22.7913	22.1167	24.0497	25.8683	24.4370	25.3513	23.8467
14.5280	14.5157	15.2220	14.9310	15.9147	15.6407	15.9477	16.2497	16.4127
11.8663	11.7697	10.9780	12.0270	11.2410	10.0413	11.1583	11.2607	11.2277
23.5817	23.2733	22.9697	23.1887	22.2900	21.6323	22.9960	22.6293	23.8423
15.8063	15.8867	16.1700	16.6200	16.8237	16.8673	16.9680	17.3043	17.4313
30.1813	30.5977	31.4620	31.2687	33.7453	36.8113	35.2240	35.9440	35.3580
20.5947	20.4640	20.8587	20.7333	22.0313	22.8173	23.1480	22.9997	22.3463
19.5260	20.4827	21.7230	20.6060	22.0550	24.4713	23.5033	24.2807	23.9940
12.0770	11.8010	12.0073	12.5173	12.2860	12.4000	12.6873	12.5403	12.5010
11.7253	11.8787	11.5833	12.7633	12.3227	11.7130	12.6953	13.0553	13.3930
24.8083	25.1513	25.8403	24.7633	27.2020	29.6720	27.9657	28.4773	27.7200

13.4183	13.1383	13.6807	13.1483	14.3757	14.8050	14.7370	14.7360	14.9817
10.2090	11.0217	10.6420	10.8253	10.5827	10.2030	11.0827	10.9640	11.3570
8.6363	8.7657	9.4587	9.2633	9.5060	10.0000	10.1630	10.2837	10.3347
13.6037	13.5750	13.9040	13.7687	14.0360	14.5563	14.3947	14.5640	14.5030
13.7647	13.6060	13.9607	13.9633	14.2410	14.9273	15.3133	15.0877	15.4397
9.9703	9.8943	9.5150	9.7733	9.7217	7.8173	9.1603	9.0277	9.9863
4.3660	4.6497	4.5907	4.9463	4.9817	6.0077	5.8880	5.9717	6.0293
7.0840	7.2963	7.4393	7.2083	7.4540	7.4910	7.5463	7.5647	7.6013
40.0113	39.6593	41.3400	39.9913	41.3510	42.6103	42.0427	42.6847	41.5747
22.6373	21.2907	21.0153	22.2900	21.0193	18.8693	20.8037	20.3610	22.1547
12.8713	12.6097	12.9317	12.9547	12.7880	13.0317	12.8310	13.3083	13.3653
8.4327	8.1007	8.5407	8.4400	8.2693	8.0827	8.7917	8.2553	8.4430
7.0317	6.8067	6.5340	6.6013	7.1977	6.7720	7.2340	7.7000	7.3617
10.8170	10.8883	11.2847	11.0827	12.1623	12.0373	12.4787	12.6663	13.4210
10.4733	10.0123	10.9327	10.7133	11.0623	11.3983	11.3450	11.2080	11.3513
14.3787	15.3137	14.1193	14.5627	15.1790	13.9457	14.3420	14.3177	14.5820
7.1330	6.9847	7.0670	7.0830	7.3483	7.4163	7.6090	7.9580	8.0157
23.5340	23.6783	23.5093	23.5777	23.9330	24.2343	23.8763	24.2080	24.6843
29.8263	30.1577	28.6453	29.7320	27.5893	25.9523	27.6670	27.4433	27.7420
13.1920	13.4050	13.7363	13.7563	14.2093	14.3840	14.4087	14.4980	14.7350
37.5347	37.9750	37.9787	38.0080	39.3023	39.2970	39.3247	39.4310	38.8767
9.4773	9.4587	9.6063	9.6427	9.5530	9.1107	9.5250	9.4397	9.6413
7.1210	7.0470	7.0977	7.4427	7.0120	7.2223	7.2840	7.4197	7.6237
18.3583	18.4903	19.9730	18.5673	20.0397	22.5360	21.4673	21.0430	20.2600
10.4137	10.3000	10.7293	10.3863	10.4200	10.6923	10.7967	11.1440	11.1263
10.2110	10.3130	10.0393	10.0323	9.7957	9.1393	9.8610	10.0333	10.2140
12.7177	13.1093	13.1400	13.7110	12.8453	12.3437	13.3643	13.2223	13.6017
19.5877	19.1353	19.0723	19.8273	19.2620	19.5600	20.0317	19.9630	20.2247
26.9157	26.3643	26.2190	27.2200	26.9167	27.6210	27.5543	27.0543	26.4650
9.0850	9.5837	9.3723	8.9680	9.0207	8.8560	9.0433	9.1097	9.0353
16.8877	16.8577	17.1293	17.5827	17.6853	18.4620	18.4367	18.6210	18.7450
3.6250	3.9923	3.9727	3.9423	3.9263	3.9993	4.0663	4.2340	4.1860
9.6487	9.1270	8.8050	9.4463	8.8470	7.7310	8.7173	8.4520	8.8963

13.0770	13.1913	12.3677	12.9273	12.1540	10.9057	11.9530	12.1383	12.2257
14.0010	13.9320	13.9437	14.2440	14.1923	14.1920	14.6970	14.9973	15.0603
12.4527	12.6130	12.9837	12.3680	13.1800	14.3847	13.4417	13.5800	12.8670
10.6697	10.5930	10.5893	10.6577	10.7753	10.5603	10.8433	11.0313	11.1370
14.1600	14.2507	14.9197	14.3050	14.8173	16.5347	15.4583	15.4390	14.7610
20.5847	21.1140	20.2803	21.1293	20.6447	19.5820	20.4190	20.8817	21.3003
23.2317	23.1493	23.8253	22.7353	23.8337	24.5533	24.4330	24.3930	23.9183
9.7573	9.7060	9.9137	10.0280	9.9177	10.0093	9.7873	9.7523	9.4013
15.6590	16.6340	17.4430	17.5120	17.2273	17.7077	18.2647	18.4107	18.5193
9.4047	10.3060	9.6777	10.4710	9.7863	9.1507	10.4870	10.6090	11.3860
34.6850	33.3317	33.8063	33.3587	34.3630	35.2523	34.9647	34.3417	34.3330
27.2650	27.7387	26.0163	27.7107	25.3307	22.8827	25.3127	24.7980	25.9700
22.9440	23.8443	25.1187	24.3447	25.7300	27.4097	26.8520	27.3627	27.0040
30.3903	30.8087	30.8980	31.8547	32.1803	33.0477	33.0677	33.5773	33.9060
10.1983	10.1433	10.0037	10.3007	10.2383	9.5517	10.4587	10.5013	10.5657
11.2617	11.2630	11.4927	12.2853	11.8373	11.9487	12.6200	13.0843	13.4027
12.8480	12.8610	14.0320	13.7617	15.0247	15.3877	15.6940	16.4770	16.2877
14.2010	14.8727	15.3707	14.8613	15.6130	16.6833	16.2237	16.4247	16.2453
8.9310	9.2683	9.0070	9.1623	8.9100	8.3350	8.9483	8.8907	9.1357
15.2057	15.8787	16.5457	16.4133	16.6770	17.5590	17.4200	18.1910	17.9277
12.3747	12.2307	12.2970	12.7517	12.2550	12.2480	12.5107	12.6880	13.0587
7.1513	7.4353	7.7420	8.1197	8.2557	8.6087	8.7320	8.9250	9.0453
12.5067	12.9663	13.6770	13.4793	13.3107	13.7317	13.9110	14.5560	14.6863
8.9403	8.8833	9.1860	8.9513	9.2713	9.0063	9.4707	9.4977	9.4890
18.9930	18.9750	18.9173	19.1460	18.6793	17.8957	19.3740	19.3733	20.2947
14.8080	14.4447	14.5867	14.7087	15.2513	15.3827	15.7230	15.6523	15.9877
11.0563	10.9143	11.3257	11.0267	11.3920	11.7163	11.7217	11.6553	11.6393
11.7477	11.9060	12.0437	11.8960	12.3290	12.9390	12.6563	12.9243	12.8963
10.2903	10.5010	10.2877	10.6410	10.1343	9.8883	10.5627	10.7357	10.8257
11.2210	11.8777	11.4867	11.8623	11.2530	10.1483	11.8733	11.6533	11.9967
15.5690	15.8783	16.2593	16.4253	16.5870	17.1757	17.2400	17.5680	17.5833

540.17nm	543.16nm	546.14nm	549.13nm	552.11nm	555.09nm	558.06nm	561.03nm	564nm
26.4390	26.4087	25.6173	24.7770	24.5073	24.2047	24.3730	24.0793	24.5297
19.5313	20.2017	19.9977	20.3243	20.5767	20.6930	21.0077	21.7377	21.0300
14.9610	15.0883	15.0843	15.1550	15.1217	15.2183	15.0980	16.0123	15.6660
27.2980	27.9123	28.6850	28.2423	28.5310	28.1887	29.3713	28.3627	29.7057
20.8887	20.9970	21.7417	22.6600	22.5037	23.7827	23.6460	23.2143	23.7877
7.3690	7.7417	7.9457	8.2477	8.4197	8.3570	8.7043	8.5113	8.3920
20.2903	20.2380	19.9783	18.6650	19.8457	19.2633	19.2520	19.9320	19.3007
21.5250	21.2460	21.6760	21.7243	21.9603	20.9857	20.2517	21.5647	21.0713
10.6957	11.0527	11.5467	10.1283	11.0830	11.6033	11.7343	11.8753	12.5820
10.5760	10.4937	10.5357	10.8533	10.2207	10.3517	10.2290	10.1790	10.2380
17.8483	17.9047	17.3187	17.5097	17.4283	16.3633	16.8113	17.8703	16.9173
12.4077	12.3597	12.1450	12.2573	11.7317	10.7800	11.2507	12.0133	10.1293
25.5990	25.8447	27.5510	29.0563	29.1357	29.2810	30.2593	29.6737	29.3040
21.7893	21.4000	20.6907	19.7360	19.9443	19.4753	18.3357	19.4640	19.0623
11.5870	11.5750	11.1157	10.7477	11.2527	10.7720	10.5480	10.0107	10.6833
22.1187	22.3737	23.1603	23.4563	23.4957	22.7720	23.6527	23.4707	24.4217
34.3440	34.0323	32.9547	30.5247	31.7373	30.0147	29.5927	30.6407	30.6763
11.0643	11.2840	10.6670	10.6680	10.9237	10.2503	10.3607	10.7627	10.6430
10.3010	10.5923	10.8457	11.3800	11.0327	11.6210	12.0873	11.7790	11.4877
25.8153	25.7737	25.6383	23.7863	24.0680	24.4197	23.3237	25.7257	24.5107
16.5363	17.1767	16.9470	17.5583	17.7387	18.1243	17.6320	17.9243	18.2300
10.8987	11.3330	11.8210	12.7557	12.5507	12.6770	12.8277	12.9273	12.8857
22.9513	23.3690	24.9197	23.5437	23.9627	23.4677	23.7830	24.2187	23.7190
17.6623	17.7570	18.3810	18.6510	18.6407	18.5557	18.9027	19.1513	19.3310
37.0000	37.3363	36.4733	34.4093	36.4260	35.7703	34.7640	36.6783	36.4237
23.0063	24.0550	23.7183	23.8947	23.5613	24.0237	23.4063	23.8383	24.1570
25.2773	25.9413	25.5290	24.8147	25.0693	24.2787	24.6393	25.6250	25.9210
12.6290	12.8180	12.8810	13.2593	12.9637	12.8950	12.5160	13.1977	12.8337
13.3470	14.2637	14.2817	14.7340	16.3113	15.1140	16.3127	17.1887	17.1097
29.4990	30.2910	29.1383	27.1557	27.1803	26.7063	27.5543	27.7807	28.3237

15.2383	15.1833	15.1110	14.7297	15.2627	15.0300	15.3050	16.0187	15.7297
11.2297	11.2500	11.7170	11.8677	11.9617	12.6037	12.5033	12.2247	12.1840
10.7547	10.8550	11.0160	10.1880	11.0877	11.3913	10.8600	11.2153	11.4387
14.7007	14.9657	15.0507	15.2017	15.2823	15.7830	16.0277	16.1387	15.8223
15.8253	16.2453	16.2537	16.5043	16.6960	16.5500	16.7967	17.7270	17.4897
9.1773	8.9657	9.5067	10.6227	11.8680	11.1193	11.4523	11.3353	12.5873
6.4720	6.6810	6.2323	6.5747	6.5090	6.2547	6.8753	7.1797	7.2167
7.5793	7.5747	7.5700	7.7023	7.8637	8.3347	7.5500	7.8083	7.9357
41.5047	42.6020	41.3027	39.1037	38.0200	37.8293	37.7033	38.0090	37.4307
20.9133	21.0477	22.3460	23.9407	24.1660	24.7880	24.5843	25.8210	25.6967
13.3120	13.7243	13.6560	13.7177	14.4480	14.1080	14.2940	13.8170	14.5577
8.5273	9.0697	8.7857	8.8197	9.7080	9.1707	9.1987	9.3877	9.9937
7.3273	7.7290	8.7017	8.4590	8.8557	8.1847	8.0577	8.4040	8.9900
13.6623	14.4367	14.4567	14.4353	15.1903	15.9350	15.3587	15.3380	15.3680
11.8203	11.6507	11.3850	11.5040	11.3757	12.0520	12.1517	11.5310	11.8707
14.7457	15.1227	15.8480	16.0370	17.1610	16.5270	16.6983	16.9953	17.3440
8.0697	8.2117	8.4957	8.4633	8.7470	9.0523	8.8697	8.8433	10.1700
24.4753	24.4017	24.2900	23.9173	24.0487	23.4323	23.9397	24.3553	23.7103
26.4733	27.1790	27.7913	29.7947	28.6933	29.9393	30.0293	29.1413	29.3880
14.4793	14.7027	14.7487	14.5520	14.6130	14.3787	14.3143	14.5320	14.2213
38.9373	39.3920	38.8317	38.4933	37.9480	37.9427	38.2010	37.7497	38.2327
9.4247	9.7810	9.7123	10.0787	10.2843	10.8327	10.4687	10.1913	10.4567
7.4080	7.8730	8.1100	8.2137	8.1487	8.4570	8.3233	8.3647	8.5133
21.2637	21.2477	19.8817	18.1357	19.2280	18.1990	18.5517	18.2087	18.3043
11.5043	11.8333	12.1237	12.0933	12.4713	12.5777	13.0830	13.0140	13.7703
9.8987	10.2813	10.5500	11.0643	11.1197	11.2643	11.7090	11.4633	11.5357
13.2883	13.5873	14.3560	13.5510	14.7420	14.7123	14.8740	14.7890	15.0993
19.9137	19.7710	19.9207	20.1890	19.3163	19.5347	19.2807	19.5993	19.6130
26.7333	26.5877	26.3593	26.2487	25.9143	24.3603	25.1600	25.5053	25.4163
8.7547	9.0070	9.0197	9.1430	8.7820	9.0267	8.7470	8.9150	8.6187
18.9630	18.9270	18.7923	18.7070	18.6843	18.4193	18.0747	18.5897	18.2830
4.3147	4.3710	4.3803	4.5303	4.2987	3.9600	4.3323	4.3703	3.9590
8.3840	8.7707	8.9693	9.9587	9.7420	9.7487	9.4530	10.2103	9.9793

12.0777	12.4557	13.0173	14.4727	14.1907	14.6877	14.8290	14.3167	14.4337
15.1507	15.4303	16.1123	16.1967	16.2390	16.8407	17.2517	16.6700	17.0153
13.1790	13.3020	12.3323	11.4990	11.4240	10.8413	10.8467	11.0473	10.6863
10.8507	11.0393	11.0800	11.4977	11.2383	11.2627	11.6543	11.0770	10.7140
15.4693	15.1520	14.6990	12.6763	13.1183	13.1423	13.1797	12.9740	12.6857
20.7930	21.2813	21.6780	22.6477	21.9487	23.5663	22.7213	22.9043	22.3037
24.7957	24.3987	23.6160	22.5013	23.2167	22.7713	22.5340	23.7020	23.0217
9.5177	9.7380	9.4823	9.4060	9.2133	8.8170	8.8083	9.2023	8.8753
19.7063	20.0983	20.4830	20.5273	20.8930	21.8097	22.6840	22.0483	23.1160
10.8417	11.5320	12.1347	13.7643	13.7800	14.7240	15.2917	15.5627	15.7470
34.8013	35.4117	35.3263	34.9873	34.7770	33.8593	34.2810	34.7137	34.3630
25.3233	25.8080	27.4190	28.7997	29.5370	29.4123	30.6497	30.7980	30.8047
28.0180	28.3633	27.6623	26.2287	27.4430	27.5157	26.1020	27.0827	27.8483
34.3383	34.8263	34.6773	34.4843	34.7053	33.8497	33.6637	34.3750	35.1673
10.5440	11.1477	11.5407	11.9213	12.2063	12.3440	12.7723	13.1807	12.9507
13.1397	13.9877	14.4670	14.8673	15.4133	15.2017	16.0730	16.5397	17.5233
16.7637	17.2117	17.6467	16.7347	17.6127	17.0893	18.2860	17.9473	17.9297
16.8630	16.9903	16.5233	15.6493	16.4123	15.6400	16.3127	16.3950	16.2117
8.8603	9.4090	9.4633	9.8803	9.9340	9.8550	10.5610	10.3807	10.5147
18.6073	18.7633	18.6210	18.5680	18.0260	18.8267	19.6333	19.6760	19.4210
12.8450	12.9360	13.2973	13.8257	13.6117	13.9680	13.9860	13.9663	14.1993
9.2063	9.2477	9.4420	9.2843	9.2417	9.7620	9.9850	10.1920	9.9050
14.6960	15.0577	15.3557	14.6077	15.0843	15.3903	15.8067	16.0880	16.0313
9.5313	9.8173	9.7337	9.9470	9.8523	10.0740	9.8837	9.8707	10.6283
19.8127	20.8960	21.6007	23.1580	22.9213	23.0773	23.8527	24.2030	24.1657
15.9543	16.1523	16.2780	16.1187	15.8870	16.5653	16.0977	16.4737	16.5407
11.6263	11.8173	11.7307	11.4457	11.6253	11.5130	11.5080	11.2093	11.2973
12.9550	13.2860	13.3803	13.2537	13.3210	13.5720	13.3773	14.2013	14.0663
10.6313	10.8387	11.4853	12.1200	11.8790	13.0780	12.7363	12.5583	12.6997
11.9163	12.1493	12.8240	14.5187	14.0543	14.8747	15.4483	15.1530	14.8133
17.9627	18.2363	18.0960	18.3690	18.6597	18.4030	18.5857	19.4147	19.1853

567.16nm	570.12nm	573.08nm	576.04nm	579.18nm	582.13nm	585.08nm	588.02nm	591.15nm
24.0900	25.1890	24.4333	24.5790	23.5690	23.1097	23.2637	23.9460	23.3213
21.3530	21.4887	21.7550	21.4503	21.5417	21.3957	21.7687	21.5717	21.4273
15.7467	15.5103	16.6640	16.3697	15.9560	16.0360	16.8470	16.0330	16.0537
29.2903	29.4657	29.7700	29.7553	29.9953	29.5840	28.9040	29.9043	29.9837
23.6737	23.9370	23.6443	23.8817	24.1143	24.5133	25.6273	24.4903	25.4820
8.6450	8.4583	8.2983	8.2907	8.4543	8.9443	8.8897	8.9870	8.8757
20.8093	21.6253	21.1950	21.0790	19.9603	19.5693	20.9523	21.4083	20.3283
22.8357	21.3973	21.7703	22.2320	20.5370	21.5253	20.3450	21.3020	21.9650
11.4770	10.9253	10.9127	10.9627	11.3860	11.8227	12.2483	10.9550	10.7707
10.0383	10.2943	10.5983	10.7270	10.1383	9.7173	10.3043	10.5477	10.7103
17.2910	17.3893	17.0913	16.5533	17.3640	15.7807	15.5490	15.2043	15.7360
11.6797	11.2533	11.5353	11.0990	10.6440	10.5330	9.8453	10.0903	10.6680
28.6650	28.4980	29.3863	29.1540	29.3287	29.4710	29.3443	29.8713	29.9933
19.4850	19.3453	18.9730	19.2957	16.5117	17.1300	16.2723	16.6843	18.0017
10.7010	10.3113	10.4917	9.8897	10.2590	9.4800	10.0237	9.8067	9.7743
23.5743	24.2083	24.3170	23.9387	24.8043	23.6487	23.8817	23.9733	24.6017
31.8520	32.1443	32.8617	32.2873	31.0723	30.2907	26.5377	29.7487	30.1053
10.8933	10.4980	11.0300	10.8983	11.0627	10.3963	10.1803	10.4883	10.6243
11.5923	11.8773	11.7003	11.5247	11.9043	11.9393	11.2253	11.8110	11.8777
25.8073	25.7033	26.9530	26.2717	25.7223	25.7557	24.4743	26.2310	26.0570
18.4873	18.1267	18.1503	18.1900	18.8670	17.9303	18.4147	18.0437	18.9487
12.6667	11.9990	12.8007	12.7140	13.2770	13.4543	13.0197	13.2973	13.7137
24.2040	24.8050	24.0150	23.3387	23.0510	24.6047	22.4753	23.1157	23.1867
19.3463	19.9033	19.6740	19.7753	19.3800	20.2583	20.2613	20.8000	20.2963
37.7167	36.9403	39.7463	38.5457	37.0567	36.6123	37.2947	38.0567	37.8353
24.9157	25.1827	25.1493	25.9503	24.0617	24.4167	24.2070	24.0740	25.5610
25.8470	26.4840	26.7917	27.2540	25.8667	26.5153	27.1973	26.0933	26.5720
13.1550	12.7910	13.1223	13.0527	12.8610	13.0560	12.5487	12.7650	12.6823
17.6450	17.1037	18.4627	18.2103	18.9020	19.2800	18.9640	19.1050	20.6463
28.1650	27.6550	29.5747	28.4207	26.3307	26.4403	26.6643	27.2677	26.3787

15.5983	15.5537	16.3467	15.9727	16.1003	15.5500	16.2263	16.1180	16.3770
12.5213	12.1663	12.6060	12.8167	12.3857	12.6430	13.3430	12.3907	13.5110
11.3733	11.6550	11.7580	11.6793	11.6800	11.2147	11.6370	11.5767	12.2027
16.5650	16.1240	15.6450	15.7710	16.0330	16.4117	15.6450	16.5297	16.6920
17.5820	17.9623	17.9653	17.6317	17.7873	17.8880	17.6203	17.9823	18.2090
11.1927	11.4377	10.8667	11.1917	11.2760	11.5830	11.8510	10.9900	11.7293
7.4063	7.5343	7.6137	7.8793	7.3393	7.3380	7.5450	7.6827	7.9200
7.8213	7.9047	8.4457	8.1747	7.4117	8.2360	8.1880	8.2583	8.1687
37.9937	37.8890	38.8333	37.8280	36.6460	35.5440	35.4810	35.8293	35.4100
24.9813	25.0430	24.9543	24.7390	25.3907	25.4457	25.3107	26.5867	25.7413
15.1307	14.5733	14.6203	14.2070	14.7587	14.6743	14.0993	14.5587	15.2960
9.5080	9.1930	9.6577	9.6130	9.1090	10.1110	9.5823	9.4643	10.3630
9.8123	9.3387	9.5637	9.4007	8.0943	10.2823	9.3793	9.3120	9.9210
17.1580	16.8307	17.0973	17.0887	17.2453	17.0557	17.2740	16.6880	17.7587
11.6920	12.0257	11.9180	12.0973	11.2943	11.6670	11.6090	11.7313	12.2120
16.6837	17.2460	17.2803	18.4920	17.7707	18.0337	18.5153	19.7673	19.5750
9.5393	10.0473	10.2183	10.4980	10.8983	10.5200	10.7967	10.6423	11.1300
23.8403	24.1177	24.4677	23.3250	22.9070	23.0607	22.8717	23.7550	22.9717
29.0713	28.9133	28.2673	28.6037	29.3587	30.1940	29.5793	29.5577	29.1280
14.4403	14.0950	14.4150	14.3323	13.9877	13.9720	14.1177	14.2250	14.1340
38.6727	37.7040	39.0640	38.0917	36.5860	35.8497	37.0007	36.8477	35.9373
9.8997	10.2967	10.4477	10.1320	10.2610	10.3320	10.3693	10.2047	10.4000
8.1843	8.3133	8.2783	8.3513	8.4350	8.1827	8.6203	8.5807	8.9933
18.3267	18.5150	19.0703	18.0690	17.1640	16.3887	15.6030	16.7277	16.0777
13.3583	13.2010	13.9243	14.2477	14.3927	14.5450	14.1073	14.5670	14.4517
11.3487	11.7453	11.0293	11.5460	11.6450	11.6427	11.9820	11.9030	11.8223
14.8113	14.9687	14.8447	15.3273	14.4857	14.4940	15.3410	15.0877	13.9627
19.9333	19.5127	20.2710	19.9697	19.1870	18.8520	19.7950	18.8680	18.5110
24.8340	25.3270	25.1183	24.8580	23.9690	23.3860	23.4830	23.8197	23.3640
8.6003	8.6290	8.8963	8.6920	8.5117	8.4620	8.7207	7.8687	8.5087
18.8597	18.8150	18.5513	18.3067	18.1097	18.0180	18.1290	18.1657	17.5850
4.2010	4.4070	4.5007	4.6273	4.1157	4.3293	4.2673	4.0687	4.4753
9.6297	9.5600	9.5743	9.6343	9.9323	9.9840	10.3483	10.1223	9.4963

14.2593	14.2153	13.4133	14.5367	15.0687	15.3883	16.1463	15.5443	15.4183
16.9227	16.8800	17.5357	17.5593	17.3513	17.5820	18.0387	17.6763	17.9810
10.4723	11.0040	10.7957	10.7560	9.7097	10.1460	9.3510	9.5820	10.0180
11.3780	11.3080	11.3227	11.5663	11.0737	11.6200	10.9937	11.3120	11.5630
13.3587	13.0040	13.2607	13.4720	11.6337	11.5050	11.4000	11.1053	10.5797
22.6630	23.5503	22.5020	23.5060	23.3307	22.8727	22.6677	23.4267	23.1080
22.9940	22.8310	22.8533	23.3093	22.2940	21.9320	20.7993	21.6280	22.0770
8.7610	9.2083	8.9577	8.8740	8.2897	8.3337	8.3857	8.1243	8.5017
24.0163	23.1143	24.4953	24.6263	24.4793	25.4240	25.4477	25.3673	26.2233
15.4213	16.0480	16.0193	16.9000	17.4560	18.5277	18.1083	17.7867	17.9447
34.7990	34.9960	35.0240	35.1227	34.4507	34.0273	34.5083	35.5790	34.9577
29.4323	29.8073	29.6557	30.7337	30.3090	30.7120	30.2147	31.2257	30.4833
27.8397	27.7387	29.5197	28.3637	27.4760	28.3377	27.2750	27.4383	27.4760
34.8320	34.7057	35.4443	35.2080	34.1627	34.7073	33.8323	34.5950	34.0670
13.1680	12.9547	13.2213	13.5947	13.9277	14.5220	14.0567	14.2720	14.2010
17.2883	17.7877	17.7330	18.5043	19.2413	19.1043	20.9320	21.5067	21.1610
19.3727	20.2973	20.0717	18.8997	19.5957	19.4507	18.1183	19.3993	21.2457
16.8127	16.8497	16.7470	16.6030	16.0663	16.3330	15.6030	16.9463	16.5730
9.9740	10.4030	10.5097	10.7560	10.9943	11.0703	10.7003	10.4027	10.6103
20.5343	20.6070	20.6760	20.6700	19.9047	19.9123	20.1683	21.3317	20.7900
14.3807	14.7460	14.8813	14.8947	14.7610	15.2907	14.9823	15.7657	16.1107
10.2967	10.4127	10.4283	10.5233	10.2020	10.7263	10.7200	10.5223	10.6517
15.9117	16.3303	16.5460	16.2503	16.5210	16.0003	16.3857	15.8990	15.6097
10.2970	10.3250	10.6610	10.3040	11.1837	10.8860	10.1370	10.7000	10.3037
24.4527	24.6007	24.5207	24.9690	25.4200	25.0987	26.0943	26.1167	26.5563
16.3690	16.5693	17.1247	17.0280	17.1307	16.9333	17.1403	17.0333	16.7267
11.5333	11.4800	11.9310	11.6047	11.4097	11.3587	11.6150	11.7723	11.6287
14.0427	14.2340	14.7310	14.3160	14.3680	14.9443	14.6143	14.8170	14.4947
12.4760	12.5123	12.6193	12.7933	13.4460	13.1690	13.8470	13.7253	13.6013
15.3530	15.3040	15.0630	15.4770	15.6807	15.8987	16.6457	15.0093	15.5687
19.6743	19.7007	20.5247	19.7140	19.8167	19.7107	20.7790	20.6660	20.2097

594.09nm	597.02nm	600.14nm	603.07nm	606.19nm	609.1nm	612.02nm	615.13nm	618.03nm
23.3190	24.0290	22.9403	23.6933	22.4103	22.8637	22.9790	22.3393	22.8313
22.3100	21.8907	20.8387	22.1727	21.1677	21.6483	21.5250	22.4177	21.0600
16.0933	16.1473	15.9387	16.3147	16.4477	16.3180	15.7813	15.7840	16.0663
29.2203	29.8960	29.5773	29.1700	29.7413	30.1540	28.6727	27.3540	28.6247
24.4827	23.9027	24.7033	24.2760	24.5967	24.5047	24.9227	26.0213	25.2233
9.1927	8.3393	9.1277	8.8500	9.0803	8.9520	9.2700	9.5933	9.3453
21.9863	22.0687	20.9080	22.4247	21.0217	22.3187	21.1120	21.0893	22.4727
21.5880	20.9823	20.9930	20.8037	22.2397	21.9130	20.3673	20.9800	20.0853
11.6920	11.0270	11.3917	11.3473	11.3770	11.8383	11.3557	11.9263	10.6573
10.3997	10.2840	9.8180	10.0877	10.1053	10.2940	10.5907	10.9577	9.9790
15.8930	16.4310	15.3237	15.4950	14.5977	15.5813	15.1110	14.4817	17.0303
9.6960	12.0967	9.2957	11.5473	10.2040	10.4790	9.5517	9.6170	9.7507
29.0157	28.5140	29.4233	29.0813	29.0817	28.9027	28.4560	30.1257	29.3483
17.9003	18.2353	15.7957	16.7117	16.7867	17.4260	14.6303	16.5913	14.8020
9.9310	10.0957	9.3220	10.0900	9.8503	9.7563	9.3207	9.0287	8.7487
23.4333	24.0790	23.3330	24.1757	24.4460	24.2543	23.7087	23.5960	24.1700
30.2380	32.1737	29.5907	31.2577	31.4397	31.2703	29.5567	28.7500	29.1803
10.6750	10.8483	10.3793	10.4147	10.6557	10.9550	10.0297	10.4620	10.5960
11.7460	11.4597	11.8287	11.5873	11.6613	11.4290	11.6097	12.2077	11.9850
26.8497	27.4253	26.0960	27.5920	28.4643	27.5530	26.5340	25.9203	27.5263
18.7477	18.2253	18.7363	18.2923	18.9683	18.4617	19.0300	18.3987	19.4523
12.8643	12.1880	13.0467	12.5067	12.8537	13.1443	13.6717	13.7063	13.0530
22.5660	24.0787	22.8810	22.9283	22.7720	23.4063	21.3957	22.5287	22.8920
20.6467	20.3317	20.3497	21.0187	20.6357	20.0330	20.4590	20.8583	20.5480
38.4417	41.3810	39.8187	41.9673	40.9287	41.8940	39.6810	39.0783	40.3593
24.1900	25.1050	24.1040	26.0753	25.2797	25.7303	25.1577	23.5423	24.0443
25.5827	28.3100	26.3093	27.2150	27.1770	26.3420	26.6693	25.6117	25.8333
13.6567	12.7593	12.8627	13.0123	12.4663	13.0810	12.3053	12.7650	13.3780
20.8750	21.4473	21.9407	22.2407	22.4367	21.5723	21.8733	23.1457	21.8597
27.7510	28.3377	25.5450	27.8183	27.4013	27.3813	26.2080	25.4410	25.0763

15.5543	15.7470	16.3003	16.1403	16.3367	16.6333	16.5187	15.3023	16.9240
13.3833	12.3290	13.0247	12.4100	12.6053	12.9453	12.8570	13.7497	13.5547
11.7080	11.6277	11.9537	12.6713	12.6037	12.1183	12.0887	11.9427	11.8840
16.3073	16.0730	17.0740	15.6970	16.2550	15.7157	16.4950	16.1820	16.4110
17.5200	17.7533	18.7133	18.0990	18.3170	18.4457	17.7660	17.4423	18.3530
11.4793	11.4540	11.0590	11.6193	11.4587	10.9323	12.5523	11.7040	10.8353
8.6630	8.0830	7.4893	8.7333	8.3190	8.1380	8.5983	8.2887	9.2547
7.9893	8.3863	7.9093	7.9880	7.3657	7.6280	7.1767	7.2750	8.4010
35.9803	37.1397	34.1930	36.0297	35.1917	35.3523	35.4260	33.3667	32.4643
26.3903	25.7250	26.2413	26.2873	25.2257	25.5180	26.0190	26.8370	26.2897
14.8927	14.1217	15.5843	14.8733	14.6007	14.1547	14.0857	15.3600	15.4307
9.7300	9.8147	9.2047	10.0893	9.4723	9.5283	9.0537	10.5703	9.0687
9.6623	10.0733	10.8043	11.4947	9.9047	9.5090	11.0770	10.5360	10.9907
17.8913	20.2190	17.5830	18.9313	19.1580	19.3760	18.7687	19.3807	19.3843
11.1070	12.0190	11.9033	12.4663	11.9150	11.8410	12.2163	12.0040	12.3527
18.9937	19.5683	18.8310	18.6960	18.3730	19.5497	19.9213	18.7050	19.8160
11.3990	11.1453	11.8720	11.1520	11.2790	11.7420	11.5540	12.1530	11.9123
22.9973	23.2497	21.5113	22.8480	22.3387	22.5330	21.6887	21.9547	21.1557
28.9417	27.9743	28.1970	28.5270	29.5370	28.7553	29.0373	28.7807	28.8903
13.8087	13.9130	14.0997	14.0293	13.5710	13.7220	13.9763	13.7510	13.1127
36.4260	37.8053	36.1847	37.0937	36.3990	36.4583	36.1967	35.5563	35.3383
10.3547	9.8347	10.3320	10.2577	10.0093	10.2897	10.1637	9.9950	9.9653
8.9243	8.8850	8.9517	8.7757	8.9560	8.6787	9.0573	8.3610	9.6723
16.2940	16.6167	14.7763	16.1483	15.8843	16.4200	14.0153	13.8977	14.0640
14.5550	14.4873	14.1660	14.5840	15.0517	14.8107	14.6667	15.4917	14.9797
11.7943	11.6033	12.0913	11.9477	12.0530	11.5140	12.3583	12.2837	11.6927
14.2200	14.9860	15.5073	14.3863	14.6780	14.4500	14.5793	14.4343	14.8177
18.8037	19.4910	17.8667	18.9920	19.2897	18.6340	17.9030	17.3343	17.1673
23.8197	23.9850	23.1403	23.2097	23.0183	23.1043	21.5440	23.0260	22.2053
8.3317	8.2260	8.3940	8.1437	7.8097	7.9867	7.8303	7.3233	7.4093
17.8543	18.0463	17.8750	17.4037	17.9477	17.7173	17.5443	17.5703	17.8813
4.7977	4.8020	4.5353	4.3417	4.4060	4.7273	4.3860	4.4210	4.4187
10.1500	9.5370	10.1077	9.6047	9.9173	9.3577	10.6510	10.4740	10.0663

15.2230	14.3283	15.7763	14.4507	14.8763	14.1930	15.2987	15.8430	15.3740
17.8510	17.3153	17.6737	16.6490	17.2853	17.3787	17.4567	17.8333	17.0733
9.3437	10.6113	9.0687	10.0553	9.6970	9.2367	8.7180	9.0480	8.4210
10.7027	11.3800	10.7707	11.1213	11.1080	11.1580	10.5057	11.1203	10.8170
11.8167	12.6503	10.6310	11.2913	11.5097	11.7250	10.2113	10.3073	9.4117
23.2603	22.6013	22.2163	22.2637	22.0423	22.7373	21.9163	21.9057	22.5107
21.7967	22.0287	20.9973	21.9550	20.6957	20.8767	21.0143	21.3427	20.5337
8.3917	8.9683	8.4070	8.4997	7.9207	8.5457	8.2497	7.6203	7.8903
26.7810	27.3370	26.4930	27.9880	27.6333	27.0070	27.8190	27.4823	27.2237
18.3400	17.9350	19.3990	17.8100	18.6337	18.6553	19.8637	19.9657	20.2760
34.1933	33.6950	32.5820	34.2147	32.5840	32.8897	31.1623	32.2010	33.5643
30.4593	30.0960	29.5043	29.8557	30.0300	30.2253	29.8243	30.8580	30.0083
26.3383	28.5757	26.6647	27.8640	28.4523	28.2033	26.7413	25.5953	26.7827
34.7040	36.6183	35.3090	35.1993	34.3233	34.5587	34.1317	31.9920	33.4440
14.0617	13.6427	15.0673	14.1123	14.6210	14.2417	14.5920	14.1793	14.9183
21.5670	21.8797	22.6963	22.7180	23.2840	23.9803	24.6360	24.7643	25.0343
20.1117	21.0773	20.2830	20.8317	21.5640	20.9887	19.4593	18.8227	19.7967
16.5170	16.6513	16.3177	16.3623	16.6140	16.5567	16.2120	16.2083	15.7173
10.7860	10.9950	11.5287	11.6273	11.4197	11.0337	11.7030	12.3557	11.8417
22.1220	23.0027	21.1530	22.5130	23.1033	21.5780	22.0113	22.2667	21.5907
16.0077	16.1050	16.8563	16.7287	16.7487	17.4250	17.4593	16.9713	18.2263
10.8607	11.4090	11.1970	11.3483	11.5803	10.8763	11.4223	10.6423	11.5510
17.1513	16.7097	16.1737	16.6943	16.9323	16.4417	16.0810	15.3870	16.3120
10.3277	10.7147	10.8097	10.7583	10.9423	10.6093	10.8380	10.6720	10.7630
26.3403	26.3093	27.6403	26.2023	27.5140	27.4350	26.6240	26.5070	27.4303
17.1047	17.7313	17.5717	17.4060	17.4610	17.2967	16.9683	17.0313	16.9177
11.7803	11.7403	11.4247	12.0533	11.3637	11.5917	11.5367	10.6910	10.9757
15.2647	15.1777	15.8867	15.0337	15.2430	15.0803	15.8503	14.8550	15.2450
13.6603	12.9740	13.8983	13.3607	13.7197	13.1907	13.9407	13.7317	14.3757
15.8980	16.3003	15.9707	16.9147	16.5577	16.3770	17.0710	15.9513	16.0720
20.4710	20.7597	20.5753	20.5540	20.4630	20.8687	20.6460	20.3063	20.2500

621.13nm	624.03nm	627.12nm	630.02nm	633.1nm	636.18nm	639.07nm	642.14nm	645.01nm
22.0773	20.6913	22.2817	22.6130	22.0323	21.1403	20.2827	18.9127	19.5710
20.8460	22.0670	21.9773	21.4910	20.9823	21.9693	19.3220	21.7433	20.7843
16.7647	16.0213	16.0887	16.4590	16.3937	16.2207	15.4740	16.0000	16.1723
28.9287	28.7077	29.0697	28.1933	27.6523	26.7747	26.3617	26.2443	26.2720
25.3180	24.6413	24.2070	25.1317	24.7693	25.8857	26.1403	25.8957	25.5723
9.0167	9.2113	8.1463	8.9523	9.2213	9.2703	8.8837	9.3107	9.2063
20.1873	21.0017	22.8703	21.2377	20.4583	20.4667	18.4307	18.1680	16.8577
20.8310	20.2153	20.9267	20.3207	19.1280	20.9373	19.9930	19.6263	18.4807
11.7807	10.8833	12.1800	10.4930	10.4737	10.8470	11.5567	11.9550	11.6423
9.5280	10.4733	10.1040	10.2193	10.3617	9.7237	10.5857	10.6553	10.8200
15.7933	14.4953	15.0720	15.3647	15.6840	14.4000	13.1403	12.7083	13.9917
8.3593	10.2687	9.8880	9.9790	8.0113	9.1203	8.0310	7.9870	9.3320
28.7893	29.8003	28.9123	28.2897	28.4423	28.1910	28.8067	27.7600	26.6377
15.0903	14.4827	16.3753	15.3057	15.1383	11.4387	13.9123	13.9280	13.9913
9.2897	8.5243	9.5777	8.8330	8.2347	8.3447	8.4757	8.8403	7.5217
23.3490	23.9983	24.1407	23.0710	23.1163	22.5780	21.7320	21.4793	22.4210
28.5043	27.1373	31.3173	28.1473	27.6593	27.3700	24.9110	25.1953	25.2387
11.2610	9.6597	10.6410	10.1457	9.8103	10.1050	9.6573	10.1637	9.8430
11.7843	11.8060	11.0600	12.2110	11.3343	11.6170	10.8983	11.5643	11.6070
26.8403	26.0900	27.2723	25.4653	26.0637	26.7570	24.6957	24.4583	24.1867
19.2343	17.5830	18.4593	18.2720	18.6863	18.4180	18.2057	18.6073	19.0640
13.5523	13.0383	13.1423	13.4717	13.5230	12.6853	12.9317	13.5127	13.1700
20.0123	21.3200	21.9023	21.8880	21.0013	21.2793	20.7027	20.1243	19.3283
20.7293	20.3837	20.1030	21.0660	20.6273	20.7360	19.5250	20.0960	20.1927
39.2943	39.8200	40.5223	38.0487	38.8277	37.8377	36.3730	35.6563	34.3800
23.6153	25.9123	24.6157	25.1250	22.8517	24.3277	22.7820	22.6810	21.3620
26.5980	25.8780	27.6543	25.7563	25.5000	25.3093	24.4457	22.5780	22.3143
13.0050	11.8867	12.5847	12.7220	11.6863	12.4943	12.4290	12.1520	11.6610
22.6193	22.0920	22.4857	22.6747	22.5000	22.4840	21.9120	20.9827	20.6897
24.2220	26.0247	27.5173	24.2200	23.7360	24.5637	24.9327	22.1057	22.7533

15.7430	16.6417	16.8663	16.1950	16.4177	16.7713	16.2350	16.7197	16.6663
12.1597	13.2723	12.8993	13.5367	13.0670	13.0333	12.6677	12.4527	14.2443
11.3210	11.2427	11.9760	11.6490	11.2033	11.2383	10.6343	11.2127	9.8733
16.5400	16.4627	15.8740	16.3217	16.4003	15.5850	16.7930	14.9850	15.9373
18.1337	17.7240	18.2193	17.4087	17.7960	17.6557	18.0083	18.2193	17.5410
13.1083	12.1837	11.1013	12.1920	12.2553	11.8750	11.7980	13.0487	12.5467
7.9203	9.0763	7.9103	7.4570	8.6147	8.1500	7.4283	7.9727	7.7040
7.9460	7.2107	7.3727	7.4637	7.9817	8.1617	7.3593	7.6117	6.6367
33.9280	33.6773	35.2690	33.1190	34.2160	32.5880	30.8533	31.7100	30.0867
25.7770	25.9267	26.6123	25.6390	25.8143	25.0913	26.2187	25.5203	24.3237
14.5737	14.8657	14.1120	15.3783	14.6270	15.5970	14.1583	13.9287	14.8593
9.5753	9.3960	8.6473	9.1863	9.0580	9.5697	9.6753	9.2567	9.6467
10.9153	11.1590	11.1190	9.7030	10.2120	10.4443	10.8557	9.8000	9.3743
17.3957	19.2290	19.4213	19.1477	18.6083	17.8903	15.6937	15.6127	18.4117
11.1140	11.6477	11.9453	10.5910	11.4710	11.1183	11.6917	10.8230	10.5313
18.2637	19.0897	20.6093	18.8333	20.8033	20.1393	19.3637	19.6240	20.1160
12.5937	11.0177	11.6040	11.3547	12.6153	11.3953	11.9037	11.4723	12.6407
21.3807	20.5290	21.5150	20.8980	21.6130	21.3200	21.2987	19.8127	19.7310
28.9820	28.5483	27.7927	27.9990	27.5517	28.6443	27.8900	27.9297	28.5713
12.8157	12.5700	13.4363	13.0187	12.3663	12.7550	12.8160	12.4857	12.3797
34.0037	35.5637	36.2577	34.6253	33.6803	33.9763	33.4437	32.0943	32.7153
10.2480	10.1507	10.1593	10.5100	9.9660	10.7440	10.0183	10.0743	9.3683
8.6633	9.2883	9.1037	9.3520	9.5723	9.5750	8.8153	9.1133	9.7913
13.5407	14.3570	15.0503	14.3577	13.2157	11.3873	11.3617	10.5430	10.1457
14.3623	14.1903	14.2860	14.3050	14.8510	14.3663	15.2977	14.7983	14.7613
11.6560	11.3710	11.4960	11.3070	12.2297	12.0550	12.0833	11.3020	12.6470
13.5763	13.9573	14.7717	13.9523	14.6970	13.7503	13.4977	13.2097	13.1150
17.8687	17.5570	17.6277	17.8290	17.1207	16.4400	16.0207	16.2913	15.6997
21.4820	21.1550	22.4037	21.0223	21.1727	20.2740	19.9380	18.8957	19.6677
8.0543	7.0237	7.8130	7.3073	6.7190	8.2197	7.7987	7.7770	6.9083
17.3947	16.7387	16.7430	16.7607	17.2443	16.4080	16.3370	15.5703	16.2450
4.2093	4.6750	4.5897	3.8247	4.6127	4.3747	4.7353	4.3677	5.0453
9.6640	10.2663	9.7910	9.7270	9.7643	9.5607	9.5290	10.0687	9.8233

16.0900	15.7350	14.5720	16.2090	15.3100	16.0770	16.4773	15.5720	16.0770
17.6740	17.1143	17.2467	17.5333	16.6363	17.3190	17.0003	17.2587	17.5857
8.4280	7.9693	8.6113	7.5450	8.1797	8.4603	8.0713	6.9680	5.9273
10.2233	10.4420	11.4120	10.7180	10.2587	9.3943	9.9763	10.0660	10.0810
9.1790	9.7393	10.0817	8.7130	10.3023	9.7377	8.6237	7.3213	7.8107
22.3073	22.1910	22.7983	20.1887	22.9267	22.4477	20.9437	20.5653	20.0753
20.6067	19.9593	20.7310	20.0463	20.2877	19.7427	19.2190	19.2600	17.4040
8.1380	8.0810	8.5857	7.3577	7.0847	7.9227	8.0960	7.9023	7.8407
27.7837	27.8163	28.5523	28.4450	27.2580	29.0223	27.1127	27.9403	27.0507
19.8493	20.3360	19.2710	19.7237	19.9797	19.9330	20.3733	20.9323	20.5090
32.2363	31.3323	32.4290	32.0437	32.8197	31.2567	31.8997	29.0690	29.3163
30.8243	30.5383	29.6300	31.2420	28.3320	28.8857	30.4373	29.4390	30.9733
25.9117	25.4547	27.3610	25.4767	25.7273	24.1180	24.0123	22.7547	24.0380
33.4143	33.1580	33.2570	32.5407	31.7063	32.8653	31.4827	30.5817	29.8900
15.1047	15.2040	14.6797	14.4897	14.6830	15.2817	14.0780	14.6963	15.6630
25.2813	25.5250	25.0720	25.2763	25.0273	26.0400	23.8177	24.1130	24.6310
19.5273	19.4187	20.5627	18.8503	19.1263	18.2147	18.2860	17.1927	17.5447
15.7530	15.5740	15.9687	15.7273	15.1587	15.1447	15.2253	15.2067	15.1330
11.1937	11.4913	11.0513	11.6083	11.8327	10.8583	11.8730	11.8453	11.8610
21.9377	22.1307	22.9157	21.2663	21.9317	22.0873	21.3577	19.4533	20.9760
16.9530	18.0457	17.6623	18.1150	17.9210	18.1717	18.4620	17.6037	17.7997
11.2057	10.8570	11.7313	11.0680	11.3980	11.5500	11.0677	11.2297	10.8537
16.0740	15.3057	15.7477	16.3727	16.0690	16.8173	14.0680	15.2963	14.7520
10.6597	10.0457	10.3177	9.7637	9.9057	10.1780	10.5407	10.5080	10.5553
26.4613	26.6133	26.4267	26.3563	26.5093	27.7170	26.8443	25.7707	25.0207
16.9047	16.0560	16.5010	16.4430	16.3037	16.2010	16.1360	16.0830	15.5880
10.7950	11.2377	10.7213	11.2007	10.4923	10.9517	10.3013	10.9770	10.4983
14.7267	15.7007	15.1830	14.7853	15.0930	15.5860	13.9083	14.5263	15.5997
13.8143	13.9720	13.0567	13.9523	13.9880	14.2050	14.8297	14.4793	15.9473
17.2000	16.4463	16.5657	15.4917	15.2547	16.5250	15.3230	16.4067	16.1253
20.4680	21.0660	20.6977	20.8097	20.3083	20.2023	19.7303	20.1250	19.8187

648.08nm	651.14nm	654.01nm	657.06nm	660.11nm	663.15nm	666nm	669.04nm	672.08nm
19.6290	18.9477	19.3793	18.7713	19.3353	18.8820	18.7977	19.1737	18.4993
21.8347	21.5903	19.5190	20.9287	21.0793	22.2520	18.6393	20.6003	20.7373
15.6933	15.5430	15.0730	15.9517	16.1570	14.8177	16.1943	15.0417	16.2337
26.3450	27.1570	25.6217	25.4150	27.1463	25.2727	24.0320	23.2500	24.5667
27.1033	25.7480	26.2987	26.6640	24.4543	26.1953	26.3290	26.1737	26.8047
9.3220	9.5660	9.0383	8.7393	7.9037	9.8997	9.5157	8.2587	9.5480
18.2593	20.0337	17.7990	19.5470	19.7807	18.4580	17.6353	18.4267	17.3930
18.9387	17.5533	18.0673	19.2053	20.1563	17.3320	16.4170	18.1487	16.1543
9.9087	11.2730	11.4243	9.7173	10.7470	10.5237	10.0133	9.1467	12.6233
10.1193	10.4233	10.3990	9.8083	9.6697	10.2917	10.3683	10.2243	9.4173
12.5260	13.1000	12.5047	13.3497	15.1750	12.9443	11.9597	13.6047	11.6997
7.7090	8.4723	8.7577	7.9190	8.5273	8.0370	8.3843	7.8660	8.1980
27.9573	27.1400	27.8827	28.2527	28.1610	27.4877	26.8697	27.7787	28.0607
13.4147	16.1503	14.1850	13.6700	13.5793	13.1390	12.1317	13.1777	13.1507
7.4510	6.5303	8.3897	7.9110	8.4827	7.5870	6.6683	7.2147	7.4753
19.9583	21.7047	22.0937	21.0437	22.4980	21.4260	19.5443	20.1520	20.1807
24.4197	25.6110	25.2350	24.6913	26.6937	23.1387	23.1237	25.7993	21.3620
8.7880	9.0990	8.6603	10.1067	10.0170	8.7467	8.5803	9.0580	8.5183
12.0347	12.1403	11.8033	11.7687	11.1850	12.8083	12.2350	12.1350	12.5593
23.0540	24.7460	22.7257	24.5827	25.3620	24.2243	21.8480	20.6750	21.4307
17.7357	17.7270	19.4227	18.9943	17.8047	17.7293	17.8037	17.7847	16.9373
13.2473	12.5453	13.2733	12.9137	13.3800	12.8767	13.5953	14.5727	13.8223
19.1820	19.9617	20.0147	19.7637	21.4830	19.3610	20.2020	19.2017	18.8333
20.5423	20.3503	19.7467	20.0633	20.6703	19.0343	20.7053	21.4030	20.2030
36.4597	34.4933	35.4763	34.9797	36.8013	30.9020	33.3953	30.2100	31.6100
22.5007	23.5027	22.8390	19.4287	22.6133	21.1660	20.2407	20.4863	20.4607
24.1993	24.3073	24.3707	22.9737	25.3370	22.6647	21.3663	22.8167	21.6917
10.4977	12.1160	11.2417	11.8270	12.0410	12.3040	10.5170	10.8643	9.9203
22.9890	20.7067	21.8080	20.3227	21.4067	21.4073	21.4623	20.5563	21.8537
22.5977	20.9960	21.0877	20.3810	23.9337	19.3213	21.6377	21.8107	19.7840

16.6203	15.2263	15.4970	15.9643	16.2533	14.6540	15.5310	15.9203	15.2823
12.7703	13.5650	13.7353	12.5463	13.3153	13.3987	14.0650	13.4753	12.9297
9.9740	10.8097	10.9040	11.5390	10.5437	10.8627	10.4013	10.2127	10.4623
16.3817	16.7663	16.5167	15.3927	15.9413	15.7730	15.8667	15.3403	15.4530
18.2253	17.2553	17.3060	16.5767	17.4063	18.0350	17.3427	17.0360	17.2507
12.7843	12.6127	12.7010	13.6607	12.2413	12.1517	12.5937	15.0517	14.0707
8.3833	8.0713	6.8980	6.2333	7.7177	7.4753	6.9007	8.3067	7.5370
6.5123	7.5007	7.4647	7.3040	8.2443	7.7937	7.7283	7.5127	7.9487
31.0990	30.2757	31.0803	29.8010	32.8267	30.5670	30.1080	30.0750	29.0747
23.3350	26.7750	24.9560	25.4970	25.3540	24.1020	25.9877	26.0530	24.9207
15.3290	14.5860	14.4647	12.6417	13.5910	14.1460	13.8430	14.7737	13.0010
9.3740	9.4340	9.9973	10.2740	9.1937	8.4703	10.3433	8.7790	9.3283
10.0603	10.7190	10.9957	12.9303	12.3487	11.9907	11.0717	12.1087	13.3767
16.8617	17.2267	16.6223	16.4170	17.8210	15.9640	15.7033	16.5837	14.7230
11.5727	11.0857	10.9127	11.1220	11.2037	10.7873	10.8077	10.9410	10.7367
19.2337	20.1713	19.4357	18.8020	19.5433	18.9670	19.7820	19.5083	19.7600
11.5613	10.5817	11.1137	10.5080	11.7970	10.6100	12.6257	11.6420	11.6020
20.1153	20.1273	18.9270	20.3620	20.3153	18.9527	18.9047	18.1663	16.4130
28.3237	26.9610	27.6957	27.6463	27.8230	27.5647	28.0987	27.0217	26.4637
11.8613	11.6447	11.9760	11.5117	12.6177	12.0220	11.4040	12.0203	11.7220
31.3903	32.1477	32.3437	31.8657	33.1570	31.0233	32.1960	31.1783	30.3107
9.2727	10.4507	9.8183	9.4433	10.2143	9.1520	10.0317	9.7983	8.8707
9.1153	9.6023	9.4270	9.1897	8.7847	9.1980	9.0470	9.5650	9.4840
10.2347	11.1040	10.2613	11.0117	11.7373	8.9240	8.4713	7.9823	9.6273
14.0183	14.1450	15.0260	14.5607	15.1623	14.4480	15.6527	13.9260	14.4860
12.6450	12.0163	12.9040	12.0303	11.8463	12.0943	12.3317	13.0003	12.6827
12.9447	14.0853	12.6333	14.1147	14.8043	12.5717	11.4963	13.6937	11.8520
16.7660	16.1027	14.3250	16.2127	17.1997	11.9670	13.8907	14.7777	14.1047
18.7127	18.7767	19.0793	17.4903	19.5577	18.8410	19.7683	17.8273	17.5410
7.3467	6.9600	6.3887	7.1933	7.0180	5.7810	6.2497	7.3013	7.0947
16.6740	15.8490	16.1990	16.0247	15.8793	15.3163	15.9850	15.5947	15.1243
4.2510	4.7780	3.7983	4.2923	4.8107	4.5217	4.4867	5.2313	4.7703
9.3437	9.9220	9.2777	9.3667	9.2930	9.6067	9.3027	10.5677	8.2350

16.1130	16.4230	16.6647	16.4807	15.5970	15.9333	17.3110	16.0710	16.6743
17.1640	18.3037	17.3350	16.6857	16.9603	16.2287	18.4203	16.8417	17.2797
7.3163	6.7083	5.8747	6.0573	7.6537	6.7523	5.9367	5.2310	4.8337
9.5850	9.5563	9.0903	9.6700	9.3373	8.2963	7.9973	9.8110	7.6363
6.7233	8.1953	7.9923	8.0803	9.1997	6.9950	6.4620	6.9390	6.3183
21.2730	20.6830	20.9707	21.4150	21.0937	21.3830	20.7910	20.2310	19.4427
17.1987	18.0237	18.1703	17.5917	17.1413	18.3207	17.1357	16.7283	17.5407
7.7963	7.9203	8.0943	7.3347	7.9650	8.4040	7.2643	7.0243	7.6167
26.1047	28.0450	26.4880	25.3540	26.4203	26.0927	23.9693	24.9647	25.6687
20.4017	20.1120	20.3643	20.8717	19.6870	20.5363	20.9823	20.2463	21.0923
29.8743	29.3787	28.5333	29.6513	29.3233	28.8210	26.7367	28.5457	28.8103
28.8147	29.1220	29.5647	28.1397	30.9070	30.2600	29.8453	28.0380	28.9420
21.8443	22.5087	21.2963	24.1483	23.3587	21.0787	21.3570	20.3403	18.4523
30.4157	28.9373	30.5067	30.5870	32.2520	30.7347	28.2933	30.1477	28.3623
14.8180	15.2817	14.9393	15.1900	14.7957	15.6943	16.6820	15.4977	16.4117
24.6993	24.3443	23.4427	24.0680	24.6153	22.4770	23.2400	22.7263	24.2950
18.7067	16.7993	17.5847	19.0010	17.2387	15.1893	16.8007	16.5950	14.2117
14.7813	14.8577	14.6113	14.5643	15.5783	15.5050	14.5003	15.1303	14.4593
12.6850	11.7337	11.8217	11.4783	11.5333	11.0080	10.8927	11.3010	11.3830
20.9457	19.9657	20.7200	20.4423	20.3417	19.3653	18.6077	19.9570	20.3433
17.5813	18.0697	17.9437	18.2637	17.6010	17.5243	17.4727	18.1017	16.9893
11.5100	10.8993	11.2627	11.3427	11.2633	10.7867	10.4703	12.2157	11.6963
15.8820	14.4403	15.9330	16.0390	17.5047	16.4140	16.2667	16.1573	15.8370
10.6323	10.8453	10.4143	9.5953	10.0213	10.5253	10.8647	10.0310	10.2767
27.1120	26.3707	27.2800	26.3377	26.3177	25.4463	25.6180	26.3297	26.1187
15.1987	15.8437	15.0467	15.4340	15.7473	15.6173	15.7020	15.2410	15.0527
10.5087	10.2527	11.1073	10.1820	10.6633	10.6497	9.8537	10.0383	9.1547
15.8987	15.4450	16.1500	14.9043	15.2900	14.6443	14.5803	14.8093	15.2120
14.2867	15.0950	15.0300	15.0990	14.4410	14.8110	15.0357	15.0963	15.0723
16.7547	15.7767	15.9823	15.9300	15.2793	15.9797	16.0330	15.2863	13.4250
20.1253	19.8810	20.1883	20.1283	19.8900	20.2040	20.6547	19.2903	20.1177

675.1nm	678.13nm	681.15nm	684.17nm	687.18nm	690nm	693.01nm	696.01nm	699.01nm
18.1993	18.7163	19.2510	16.8757	16.9040	14.9670	18.1827	16.6080	19.2363
20.1897	22.1820	21.9267	20.4547	19.9863	20.3680	20.4567	21.3600	20.8240
15.7433	17.2843	15.1887	16.1423	15.4087	14.0040	13.6613	15.6047	15.1263
22.8337	24.3700	23.8217	22.5623	26.0523	23.8757	23.0153	24.2143	23.8090
27.5487	26.6487	25.3237	25.9213	26.5287	25.8303	25.9213	26.9450	23.8923
9.9820	8.8977	8.1397	9.1753	9.8500	8.9357	7.7020	9.3973	8.1043
14.9260	17.4793	18.0747	16.2530	16.4760	15.7187	14.6080	14.5313	19.2800
18.0290	17.9637	15.6837	16.6893	18.6220	16.8540	14.5010	16.3370	16.8353
8.7047	10.3510	11.6233	9.5527	9.4307	8.4657	10.7130	10.4953	11.3690
10.9660	9.5633	9.9597	9.9213	10.8823	9.3147	9.6027	10.8600	9.5970
11.6000	12.1743	13.3817	12.9787	12.6343	12.4577	11.3220	10.1983	15.1233
6.1977	8.7783	8.1367	6.9190	7.8953	4.7250	6.2273	7.5747	9.1307
27.8107	27.1770	26.7693	25.5800	25.6593	25.5820	25.7223	27.2193	26.7993
11.1933	12.3370	11.6350	11.3097	13.0550	12.1797	11.5967	10.0897	13.8213
7.4070	6.9457	6.4960	6.4820	5.6377	6.0983	5.1593	5.1403	5.9157
19.4110	20.7007	20.4560	20.7707	18.2230	19.5600	17.7383	18.3423	21.2843
20.4040	21.1107	22.4493	22.5677	22.1887	19.5930	18.0950	17.5730	24.2083
7.0430	10.0443	9.0783	7.3480	9.0480	7.4750	6.5347	6.8457	9.9923
11.3660	12.1037	12.1370	11.7843	10.8380	11.8093	11.2947	13.3517	10.5710
20.8077	20.9693	21.7983	22.1257	20.4023	21.3157	21.7557	17.7207	21.0743
17.9083	16.9633	17.6280	17.5737	17.8427	17.6593	18.1153	17.5113	17.1887
12.4457	13.7810	12.2327	13.0180	13.4870	11.9173	11.3317	12.2523	11.3390
18.3190	18.0623	20.4337	16.7140	18.7377	16.9343	18.7620	15.5163	21.3873
19.7607	20.8707	20.5627	19.4497	19.8020	19.8033	20.3620	19.5673	19.7900
31.4190	32.3300	32.9653	30.4907	29.2030	28.5973	28.5467	29.5343	34.8280
19.1550	20.2260	21.7970	19.5193	19.1577	19.4510	19.2697	19.8837	19.0893
20.0697	22.9993	22.4357	22.4477	20.5133	22.4213	18.1673	19.8980	21.2143
10.7263	11.0413	11.1657	10.6263	10.2080	11.9123	9.2210	9.6307	10.5900
18.6233	19.4897	21.7233	20.8263	20.1460	21.1100	19.7540	20.8690	19.5983
18.2060	21.0923	21.1003	19.3790	19.6053	21.4130	18.4570	17.4007	21.8027

15.5743	16.5793	15.7053	16.1133	15.7163	16.4260	16.2423	14.8120	15.2650
13.5263	12.7123	12.5643	13.0583	13.2107	13.7043	12.9620	13.4857	13.3300
10.7810	10.0953	9.9890	9.1167	9.8400	10.9740	8.3587	8.4683	9.8210
16.1447	14.5273	15.8493	15.3873	15.4943	16.6827	15.3927	15.3513	15.1207
15.7527	18.1147	17.5030	17.7643	17.7477	18.5440	16.7923	16.5833	17.3413
12.5117	12.0487	14.5577	13.5730	14.9870	13.2527	13.6480	12.9307	12.6417
7.6993	7.3623	7.5523	6.8867	7.2880	7.0597	7.8710	6.4977	7.4597
7.8107	7.1507	7.9607	8.5397	7.4240	7.0013	5.5707	8.9297	7.5667
29.8427	28.8003	30.4427	31.2690	29.7587	28.2237	28.7090	28.1797	32.4487
25.2223	24.5753	25.3493	23.6983	24.4930	25.7250	26.1440	24.3443	24.4667
12.4063	14.5590	14.0383	12.5227	13.5373	14.2673	13.1773	12.7693	13.2743
8.8300	9.6643	8.0057	9.4887	9.8953	9.1697	9.3807	9.6590	8.0210
11.5697	10.2353	12.8710	11.7047	11.6970	13.8000	12.5300	10.6283	13.6750
15.6343	15.1037	16.8183	14.8137	15.0743	16.6517	13.8223	13.3293	15.9230
9.9823	10.1573	10.9507	10.9387	10.3410	10.6053	8.7677	12.0717	10.4637
18.5470	17.3753	18.4920	19.4477	19.6247	20.3173	17.1607	20.3550	21.7227
11.8537	11.2547	10.7690	12.1153	11.1790	12.8383	12.1200	11.6710	11.0040
19.1910	18.1897	18.3023	17.2557	16.9430	16.8210	14.9550	16.0700	16.9687
25.6740	27.4943	27.1970	27.5017	27.0510	26.7193	26.5777	25.8253	27.4283
11.7120	11.3020	11.1547	10.0920	10.9927	10.9473	10.6830	10.9770	11.6977
30.3727	29.6447	31.0093	30.0560	29.8547	31.5037	28.7803	28.7727	31.6143
8.7027	9.2140	9.5603	8.6440	9.6043	9.9777	8.9900	8.1827	8.2957
9.4233	10.2230	8.9327	10.0030	9.4493	9.1133	8.4067	8.3067	9.5493
7.7650	9.2003	9.4597	8.3940	7.7733	6.1197	7.4617	6.7613	9.7730
13.8917	14.4740	14.8707	15.6033	13.8293	14.0250	14.4397	14.3367	13.6737
12.2333	12.0727	11.3347	13.2017	12.0480	12.3263	11.2450	11.7030	11.3830
13.3170	14.4830	13.9813	11.8547	12.3080	12.9110	12.2740	10.8960	13.2193
13.8190	14.2087	15.1700	14.4093	13.3157	14.3217	11.5507	13.2947	14.9220
17.2283	18.5420	18.6953	18.6453	18.5390	16.9127	14.5627	15.2910	16.9380
7.6590	6.9023	6.6197	6.9723	5.7250	6.5017	6.1043	5.8673	6.0313
15.4387	15.2813	14.8290	15.2020	15.0037	14.2027	14.7833	15.6097	14.8430
4.3987	4.6117	4.0927	4.6257	5.0717	4.9503	4.7333	4.3620	4.0240
10.0377	9.9373	9.8597	10.2183	9.5883	9.4090	10.1817	9.0773	9.9577

16.8763	16.2753	16.5987	16.3587	17.6727	17.1940	17.7060	17.1203	14.6943
17.7277	16.9763	16.4990	17.2567	16.0527	18.0790	16.2500	16.2110	16.5077
5.2140	5.6360	5.7967	6.0593	4.7897	4.3367	5.5287	4.4030	6.1520
7.4650	9.0553	8.8860	7.9803	8.8293	7.9783	8.6610	7.3587	9.7950
5.9740	7.9810	8.0453	7.0280	5.6117	4.3980	4.8187	6.1320	7.6710
21.1150	20.2040	21.4683	22.2193	20.9387	20.4387	20.4827	19.7820	19.1360
14.9473	16.1933	16.0690	16.1487	15.0257	15.8817	14.3467	14.8187	16.7683
7.0743	7.2200	6.7297	6.5917	6.5543	6.2617	6.8203	7.4100	6.8087
26.7197	27.5967	26.2553	25.4510	25.1900	24.9073	23.6600	23.6523	25.4800
22.0023	20.5613	20.4747	21.8363	21.7743	21.4450	21.7117	21.1137	20.0340
25.7847	28.0413	27.6230	27.5440	27.2180	25.5883	26.0867	26.9357	28.5097
28.6883	29.1590	27.9467	27.9657	29.4117	28.5267	28.8797	26.2643	26.5777
18.4857	23.0757	21.9823	18.7807	17.7527	17.7500	17.7713	16.9380	21.2840
28.4160	29.4297	29.8013	30.0823	27.6610	28.1270	26.5947	27.9533	30.4757
16.9177	16.1493	15.5657	15.9300	16.7500	16.6917	15.1937	14.9790	14.4387
22.7830	22.3317	23.3113	23.5890	24.0537	22.8237	21.6487	21.7207	21.9030
13.6113	14.5467	16.1710	14.2100	14.4217	14.2590	14.5187	12.3923	18.1873
15.2457	14.6797	15.3250	15.3330	14.8863	14.4653	14.0047	13.8433	15.2947
11.1137	10.7567	10.5093	11.3433	11.0873	10.2977	12.1167	11.1533	10.5620
17.4287	19.8547	19.4140	18.1490	17.1117	16.7243	16.4833	17.3497	19.3677
18.6743	17.8090	18.2403	16.6940	18.6770	16.7803	17.3743	16.8440	16.6307
11.1070	11.7967	10.7517	10.9897	11.2010	11.3280	10.7977	10.3307	11.2990
14.7493	16.0200	16.2390	14.0483	15.5897	15.8020	14.7697	16.0763	16.7817
11.0990	10.7320	10.7323	10.7587	11.5857	10.0440	11.1750	10.5447	9.8333
24.6917	25.5247	26.3853	24.5437	25.9147	24.4070	24.5253	24.4810	26.4347
15.0243	15.1510	14.8567	14.9330	15.0087	14.0850	15.2113	13.9210	14.4103
10.1890	9.3257	10.1543	10.1857	10.1787	8.4297	8.3483	10.2533	9.2147
15.5807	14.6093	15.5193	15.2637	14.5380	14.7270	15.6767	14.8060	14.8137
15.7943	14.4953	14.1333	14.8213	15.2303	15.3957	15.1193	13.8870	14.2440
14.9717	15.5220	16.0843	16.1053	14.4273	14.9907	14.5187	14.7747	14.7543
20.0837	19.1670	19.0750	19.0067	18.7313	18.7327	19.1130	18.2213	19.2377

702nm	705.17nm	708.16nm	711.14nm	714.12nm	717.09nm	720.05nm	723.02nm	726.16nm
17.1330	15.9377	15.4053	15.1973	15.2303	17.3130	15.8770	14.7550	15.1000
20.1107	17.7843	19.4813	19.5053	19.9583	19.0517	17.1120	19.5103	15.6283
14.9667	15.2237	16.8033	16.2187	14.7407	14.3100	14.5387	13.2080	13.9533
22.2733	20.3297	20.4730	22.8910	20.3420	21.0533	19.7983	19.2867	19.0257
25.5700	26.9370	28.8143	27.8430	25.7390	27.1313	28.0093	27.8330	26.5677
10.6407	10.8393	8.8310	8.8917	8.6970	9.8627	10.5963	9.6697	9.6470
14.3810	13.7403	11.7277	11.6787	15.3783	12.9857	9.8987	10.9053	8.4137
17.3250	16.1380	15.7577	15.2487	16.3310	13.5253	14.4113	12.8580	10.6860
10.8840	10.2933	10.3860	12.1960	10.4773	10.4053	9.7697	8.0787	12.8923
9.5133	9.9030	10.6503	10.0717	8.4830	8.8417	10.3007	9.0580	7.3983
11.6463	9.2053	10.9367	10.9603	9.2300	8.4503	10.3373	7.4367	11.6657
5.5167	4.7343	4.9113	6.5977	5.9897	4.0230	3.2590	5.1823	1.8113
25.8110	25.1813	25.8320	25.5507	26.1593	24.0703	26.7803	23.9277	25.2043
10.9737	9.6147	8.8850	7.0480	9.1627	9.4300	7.6207	9.8440	8.5053
4.9687	4.5450	5.2600	6.2003	5.6463	4.1363	5.1707	2.6490	1.9197
18.7047	18.5023	17.2083	17.5493	17.7170	16.2433	18.4623	14.6890	17.4800
19.7630	16.1873	16.0297	17.7277	16.7983	14.4860	13.6140	12.7470	14.4047
6.4680	7.3233	6.0427	6.8370	6.5473	5.2390	6.1840	6.6890	5.4530
10.6380	12.5730	12.4483	11.8063	13.1260	10.0107	10.9540	12.7667	13.2800
19.9333	19.8917	18.4023	19.7767	17.9000	18.3027	17.7353	17.5100	13.7267
18.4113	18.2437	17.2297	17.3250	16.3393	17.7860	16.4790	18.8030	18.6173
13.1577	12.7573	13.7420	12.7423	13.3203	13.6397	11.8273	10.9700	12.8087
16.0253	18.0390	16.4797	16.9423	16.1513	14.3417	14.0537	14.1800	11.0483
19.7243	18.1647	19.8303	19.0670	19.4237	20.4017	19.6923	18.2730	18.6783
27.9750	26.7100	26.0970	27.0410	26.8123	27.6027	27.6203	24.4893	24.7157
17.9467	17.4587	17.9890	16.2913	17.8667	18.3820	15.0570	18.6697	14.3273
17.3977	17.5743	18.9427	19.6200	20.1540	19.0910	17.6550	13.7337	16.7090
8.9547	10.6667	8.4213	9.6783	9.5363	9.0967	10.2573	9.1617	8.6907
19.8093	16.5277	17.3947	20.6997	19.3883	20.3923	18.2133	18.2343	16.2843
16.2337	17.4883	19.4027	16.7607	17.0140	17.7313	15.0683	16.3160	14.9423

14.3253	14.4497	15.0130	14.5910	14.8727	14.2310	11.6670	15.7170	14.2857
13.5510	12.8153	11.0023	12.7613	12.5237	11.0267	11.3857	10.5467	11.0620
9.1803	7.3483	8.3180	7.5217	8.4543	7.9630	5.6523	7.0517	6.9367
15.8383	14.7073	14.0587	14.6570	15.4487	15.6607	15.4130	15.9677	14.6640
16.0803	17.9830	17.4027	16.2263	16.0973	16.8180	16.3863	15.8797	16.0647
13.7000	13.9807	15.9950	14.0180	14.3503	14.6423	14.2953	15.9297	13.7407
6.4803	5.5233	5.6380	6.0273	6.1627	6.1687	5.3530	5.5187	3.3163
6.5527	7.3327	6.7923	6.6483	5.9223	5.5373	5.3880	4.3420	5.5557
26.3473	25.5663	26.7043	27.0000	28.4547	25.1817	27.7363	28.2623	25.4927
23.9333	24.4867	23.9400	22.3487	24.8763	22.5440	23.2780	21.1937	24.2307
13.3897	12.2477	12.0523	11.9300	13.7627	11.2240	11.3377	9.9917	10.8957
9.8890	9.2723	8.7877	8.5417	8.5533	9.7303	9.0520	7.7913	6.6100
11.8917	12.0927	10.9207	11.0217	13.3233	14.6343	10.9013	14.8150	11.4413
13.7007	10.5923	11.6880	13.2437	13.2650	10.2783	13.4537	9.0887	12.5427
10.0463	9.3860	9.5333	10.4983	8.0697	9.8987	8.1637	6.5233	8.6597
19.8803	18.1470	18.2213	19.9660	19.2857	19.2780	18.5313	17.9270	20.7440
11.1010	10.3423	12.1490	11.7480	11.9720	11.7987	9.7290	12.6817	10.8230
15.3903	16.2603	15.4553	15.5303	15.6693	15.4537	15.0550	13.8623	13.3357
25.9130	24.7600	26.6550	24.3513	26.5917	25.3503	24.1510	25.6407	23.6227
10.5533	10.2627	9.5520	10.4147	10.2453	11.8100	10.4627	10.5953	9.8193
29.5037	27.0810	28.2740	28.8093	28.5800	27.0497	27.3717	26.9033	26.1330
8.7097	8.6587	8.5913	8.0623	7.3397	10.1273	7.7470	10.2270	6.6537
9.4750	10.8923	9.9197	9.9820	9.5760	8.7650	9.6047	8.3830	10.2247
6.7033	3.9953	4.1240	6.1620	3.0027	3.4133	-0.2210	1.3330	2.9023
14.8107	14.5710	14.8950	14.5003	13.2243	13.7060	14.5100	15.0723	12.8543
13.6807	11.2697	13.4623	13.4717	12.5490	11.9300	12.8730	12.9003	11.5067
10.5657	9.3687	11.2643	11.9933	11.8363	9.8247	9.8787	7.1397	10.5457
11.9653	8.5133	13.0070	13.3323	10.9103	11.4983	9.2347	11.3660	9.4697
17.0220	15.6693	16.9107	16.1303	14.2767	16.5203	13.8490	14.0560	12.8810
6.6907	5.2717	4.9547	5.6567	5.1570	5.2777	3.9027	5.2177	4.2630
14.2603	13.9680	13.3073	13.3173	13.6773	12.8677	14.0923	12.3980	12.1473
4.9173	4.1603	4.9010	3.9863	3.8303	4.1760	4.4053	3.3183	2.6023
10.4273	11.0737	8.5687	8.3883	9.9290	9.1837	11.1627	10.5753	8.1477

17.2400	17.9247	18.3700	17.5163	16.8180	16.5960	18.3343	17.0703	18.4753
14.9867	16.0503	16.3540	15.3503	14.6673	15.4473	16.4910	15.1907	14.2857
5.9407	3.9727	4.1747	3.0770	2.7543	3.3783	2.1800	0.1983	-1.6370
8.1980	7.6030	6.0917	5.4690	5.2753	6.6727	6.8223	5.9697	3.9040
4.2760	4.3877	1.8857	5.8890	5.9330	3.4587	1.0710	-2.3670	-0.0330
18.2350	20.4640	19.6167	18.5513	18.4777	19.6743	16.8880	18.1780	16.7953
14.7777	14.6797	15.0363	13.6290	14.4673	14.5763	12.4703	13.1310	11.8937
6.9900	6.5553	6.5663	6.8973	6.4523	5.4570	6.1847	6.8347	6.4490
25.1343	21.4897	21.6600	22.3553	22.9680	22.3380	22.7110	21.7507	22.4453
22.1080	23.4373	20.0100	21.1890	20.2490	20.3793	20.5920	20.7283	22.9207
27.0167	25.0950	22.4713	23.6560	24.8627	23.9153	24.8177	22.0153	25.2950
26.5430	26.2717	26.3063	26.9537	24.6343	27.2077	25.6853	25.5053	24.4607
18.8407	17.9490	15.7323	16.9753	18.4170	17.3927	15.6807	13.8280	15.4867
27.8163	25.6977	27.4417	27.9183	27.1620	26.0817	22.7600	26.3073	24.2033
16.3547	15.1140	16.2723	16.2267	14.8403	16.9103	16.8933	17.1370	18.8977
21.3070	19.0237	21.2070	20.5133	21.0113	20.8340	19.5057	17.9313	18.7600
12.4880	11.5300	10.5053	11.8900	11.9573	11.1493	11.9373	11.6620	8.6970
15.1283	14.3673	14.3303	14.5953	14.1903	12.7783	13.6663	12.0807	13.2563
12.1143	12.2353	11.0647	10.8367	10.6090	10.8757	10.0880	11.2200	10.2230
18.0183	15.0523	14.4143	15.8153	14.4543	15.1083	14.6043	12.2367	13.1843
17.5463	18.0643	16.7013	17.4003	16.6500	16.3423	16.8430	16.3330	16.3330
11.3273	10.3297	11.2797	11.7353	12.1240	10.1647	11.8453	10.5240	11.6277
16.8303	13.1800	13.9547	14.9157	13.6927	13.6667	12.1527	11.9797	12.7613
10.9957	12.7803	10.7537	11.0153	9.9443	11.3407	11.0697	11.5320	11.5340
24.3737	23.1960	23.2987	24.1840	23.3827	23.9843	22.9830	20.5847	23.4437
14.7700	13.5183	13.1833	13.0670	14.1113	13.7697	13.2777	12.3153	12.7290
9.2657	8.1827	7.5810	8.2363	7.4117	9.4537	8.0557	7.6107	7.2810
15.6487	15.4323	14.6633	14.1977	15.7530	14.2873	15.0473	14.8097	13.8263
15.2823	15.8293	16.5407	15.2340	14.9150	16.9477	17.0930	15.4860	16.0303
13.8197	11.1787	13.9697	15.6357	15.0483	14.3493	14.2653	15.6857	13.3223
19.0447	18.8093	18.5223	18.6667	18.5323	17.8773	17.5547	18.2777	16.7817

729.12nm	732.07nm	735.01nm	738.14nm	741.08nm	744.01nm	747.12nm	750.04nm	753.15nm
16.6870	14.6203	12.1357	14.6337	12.8203	15.9297	14.9250	11.8670	13.1273
18.4363	20.3257	16.0907	15.9140	18.1997	19.2203	16.5410	17.2807	15.7447
15.0813	14.2437	15.0727	14.6857	15.1937	13.5460	10.5270	14.1297	11.3443
21.9050	18.2213	17.7407	18.7783	18.8203	17.2547	16.8827	18.8867	16.9260
26.2413	27.2933	28.8617	27.8803	26.5177	29.7340	28.6940	27.2203	26.9130
9.2107	8.6963	9.0617	10.2140	9.8480	8.4837	10.0810	8.9237	10.5850
10.3053	10.4020	11.0373	9.8127	11.5830	7.1790	7.3487	6.6057	8.8113
13.4463	13.0810	11.1660	13.6937	13.2257	15.9883	12.3423	10.9553	12.7573
9.7540	9.9127	11.9303	7.7783	10.2853	7.1747	8.7187	12.4443	9.8497
9.4293	7.7893	8.0360	8.9160	10.0183	10.0323	8.7407	10.0263	9.7220
8.3833	9.6273	8.9793	7.4787	10.5270	8.0923	9.9877	9.0583	11.5550
3.4220	5.7747	2.7070	3.7513	3.0387	3.3637	5.2510	-1.2457	-0.9853
25.5340	26.2533	26.3960	25.2310	22.7820	26.5840	21.0733	26.2433	26.8757
7.5517	9.9500	9.1173	9.0197	11.0747	8.0350	5.6153	8.8683	7.5000
4.0257	3.0463	2.4793	2.8623	2.7830	2.6390	1.5963	3.8640	-0.7200
17.7083	18.4240	15.2910	17.2467	15.1870	15.2723	18.0270	15.5943	14.4570
15.4653	13.8547	13.4633	13.8587	10.8277	10.0897	8.3983	9.2147	7.1343
3.3477	6.4230	7.1223	5.3937	4.7447	0.6100	0.2247	-0.9583	3.3447
12.5473	12.0297	10.3747	12.7443	11.8593	11.1697	12.3400	11.0770	14.6933
17.6190	20.0663	17.4283	17.4393	15.9120	13.7803	17.7950	15.8053	11.1397
14.8840	16.1970	18.9900	16.8747	19.7763	16.2863	17.9357	16.0690	12.8840
13.2173	14.5590	11.8073	11.3810	8.4407	12.0353	13.5247	11.7210	10.6843
14.8067	14.1663	14.1470	11.7827	8.8973	13.0070	11.0607	8.7823	12.9653
20.7580	16.3457	17.1850	18.3050	17.3933	16.3417	13.1433	17.2443	13.5680
27.8237	25.3460	24.7307	24.7373	24.1170	19.1170	22.9953	21.3567	23.3063
14.2717	15.3693	13.9923	14.4233	18.0957	13.1910	15.9733	17.4897	13.4357
16.5623	17.1757	17.7897	16.8120	16.6993	13.4237	16.9757	13.9273	13.0580
9.2100	10.3127	7.5740	5.9293	6.6540	7.2143	4.2183	2.5417	7.4400
17.4313	17.6373	16.1830	18.6377	14.3537	15.9500	16.7917	14.4103	18.0857
12.7170	14.7440	14.5147	15.1900	14.1253	14.3787	14.1770	12.2270	13.8183

14.4467	14.8940	12.8477	13.8640	11.5180	12.0007	8.1233	14.3240	11.8283
10.4700	12.3990	10.4890	11.8617	13.0887	14.2837	11.7180	12.4683	12.1600
6.6217	9.4057	6.5807	8.4307	6.9713	7.6770	6.1910	6.5147	4.3237
13.0957	13.9193	14.9980	15.6817	16.1300	12.2103	14.1220	14.8903	13.9220
15.4673	16.9737	15.2743	14.5123	17.8940	15.4673	16.4587	16.4767	15.4140
14.6523	15.9777	15.1083	14.2523	12.9797	14.5010	12.7680	9.1587	11.6957
6.5023	5.0120	5.3830	4.2223	4.2140	4.1693	4.6307	2.7283	-0.7177
4.2167	6.2733	4.8217	2.9683	4.7127	5.6537	4.3613	4.8510	4.8690
27.3220	27.2610	26.0733	24.4443	24.2733	21.9347	24.2883	23.0957	25.8320
21.4260	22.9730	24.9277	24.2293	23.5100	23.1870	23.9103	19.5310	26.8940
11.5180	12.5050	9.1800	13.2617	15.2800	8.0550	10.9080	10.1657	10.1433
8.2470	7.6193	8.2787	6.5347	7.8970	8.6230	10.5073	7.1267	7.9413
11.7083	13.6463	10.8810	11.9730	14.0577	12.6247	9.4153	14.2387	11.3150
11.0193	15.0290	12.0533	11.6940	13.9083	9.8540	7.2987	7.5133	9.0673
5.7733	9.3653	8.1637	8.1277	6.7083	7.7957	4.4927	6.1477	11.0743
20.1983	18.5970	19.4780	16.5700	19.2897	17.1067	17.3997	18.6420	16.3367
10.0117	9.1247	11.4837	11.7380	10.9150	8.5387	10.3503	12.1473	10.0433
13.5207	13.7893	14.9787	14.8163	12.9800	12.5247	14.0753	13.8433	11.7047
23.4207	24.6233	24.5200	24.6193	24.0350	23.3430	23.7437	22.1413	24.8963
10.0493	9.9083	9.1923	10.5257	9.1197	9.2700	8.2537	8.3460	6.4680
24.6660	26.7280	26.0910	24.5887	26.0630	22.5907	21.3803	25.5560	20.0570
8.4470	8.8660	8.5783	8.3703	7.2823	7.6277	6.6513	6.2970	7.5197
9.4853	9.2460	9.1847	9.6417	10.5647	8.7047	7.6340	10.1790	7.6743
2.3300	4.9883	1.4303	2.1297	-3.6137	-1.4240	-1.2990	-1.5957	-0.3820
15.6660	14.4997	14.9200	15.8900	14.5687	15.5240	15.0823	13.6637	14.4517
13.0130	12.0997	12.9050	13.6027	14.5790	12.4963	13.6263	13.5460	12.9097
9.9713	12.6477	9.2133	9.0993	7.0127	8.5670	6.9923	9.8047	7.0180
11.8490	10.8027	8.7763	7.1293	10.2503	3.5963	7.7183	7.7363	8.1267
13.7217	14.5463	13.7247	13.3307	16.5330	10.7057	11.9567	12.1650	10.9683
5.1457	4.0413	3.8337	4.3507	4.8903	3.6427	5.5843	1.6197	3.7313
12.5930	13.1567	12.5530	11.4377	11.6637	9.7723	11.6773	11.6370	11.3923
3.1867	4.1507	4.3497	2.7547	5.0377	4.0397	2.5620	1.8427	2.2940
10.0450	9.8760	7.6300	8.5937	9.3930	10.2430	8.1767	11.4960	8.8903

18.1547	17.3030	17.9257	17.5830	19.8060	22.0770	17.0120	19.4097	19.4517
15.7710	16.8613	16.1373	15.8523	16.8897	14.0593	14.7253	14.0357	11.5920
1.4997	2.1790	-0.2257	2.4443	-1.0370	0.8733	-1.9890	-1.8137	-5.4767
5.1767	6.2173	5.2530	4.9037	6.1287	3.2003	4.0753	7.2253	4.7723
2.6570	4.5473	2.2493	-1.3267	0.4993	-1.7643	-1.9200	-0.5657	-1.4857
19.4730	18.2040	17.9930	17.8587	19.7340	17.8663	15.6027	19.1183	20.6867
13.6357	13.2650	8.6703	11.9577	10.2000	12.0807	10.9327	11.7613	10.3767
5.5923	6.7570	6.1283	7.3530	5.7803	6.4487	7.1970	6.4557	5.4687
24.0103	21.3020	21.4913	22.5230	20.2037	21.8377	21.0053	22.3503	21.3623
23.9363	21.3943	19.4963	21.3410	22.2447	20.7627	24.0700	20.8567	23.5150
23.7310	23.9930	23.0647	21.0850	23.0083	20.5757	23.4140	22.8210	18.2003
27.0937	23.9623	27.3033	26.2157	26.6713	22.4840	25.0787	22.9660	25.0330
17.0070	14.6157	15.6307	13.1027	13.2667	13.1490	13.1237	12.6113	10.7470
26.5473	24.6713	20.9937	22.5110	24.5780	21.0687	21.6707	22.9260	18.7203
16.3920	16.4867	17.5593	18.0673	17.2243	17.8927	19.8393	17.5367	16.8173
18.9033	18.1917	18.8763	20.7613	16.6467	19.5220	19.0787	15.3213	16.9960
10.5657	12.3437	10.0950	8.9600	7.3163	6.9673	7.8643	7.9883	3.0390
12.6237	12.8667	10.3933	12.4490	11.5943	10.6703	10.7227	13.0520	8.8680
10.3960	10.2317	10.7767	11.3070	10.0977	9.3087	11.7470	10.8417	12.0627
14.2713	14.4750	12.2783	11.8187	13.7180	9.9640	12.8497	13.5570	8.2133
17.5783	15.6737	16.6760	15.6493	16.4990	15.6933	14.6757	15.3747	14.3947
11.1607	10.2813	9.3833	11.4100	10.3093	9.8480	11.2833	9.9050	11.5827
13.9427	12.9720	12.9173	13.1503	12.0277	8.1403	12.0447	10.3103	10.8623
10.0253	10.5437	9.9537	10.2997	10.9923	11.9113	12.9053	8.7910	9.1410
24.4670	21.3747	22.1750	23.1987	24.4463	21.2443	24.9700	21.0633	21.7380
12.8490	13.0073	11.5537	13.5560	13.9970	10.9277	10.9360	12.5313	12.7023
8.5057	8.3817	5.1283	7.2297	5.6423	4.9220	5.7937	6.1050	5.7583
15.9390	14.7637	15.6043	16.7353	15.2703	15.2797	12.9660	14.9650	15.1633
17.5190	15.9213	15.7120	16.2157	16.4563	16.2550	17.0287	15.6473	16.5657
12.1527	11.5230	12.0643	13.3400	11.0727	12.8573	13.3927	9.9587	11.1797
18.3313	16.9097	18.6073	17.6493	17.4973	16.2543	16.7953	16.9530	17.6727

756.06nm	759.15nm	762.06nm	765.14nm	768.04nm	771.11nm	774nm	777.06nm	780.12nm
11.9653	14.6873	15.8563	15.4230	15.9880	11.6393	13.7680	13.6803	13.1403
15.8143	13.2213	17.3330	15.9643	16.9570	15.9083	14.7980	15.7550	15.5840
11.8133	13.8833	12.7933	14.8403	13.4520	8.9870	7.7820	10.7430	7.6077
19.7073	19.0783	20.6410	17.1223	15.0517	16.4457	12.7700	12.3227	15.2200
26.1903	29.0827	27.5117	25.3953	26.6730	27.1403	28.8333	24.8133	26.1767
10.9370	9.1247	9.9243	8.2223	7.7267	11.1320	10.7553	13.0520	12.1667
2.6567	5.1197	10.3367	10.7217	6.5077	6.2423	1.5963	4.3447	-0.9813
13.5583	12.2213	13.1913	15.3963	11.1110	10.5273	9.8150	9.3337	7.3433
11.4250	5.7980	9.3713	9.8370	8.8990	10.1613	9.5470	10.5200	10.4780
9.1700	8.2843	8.1873	10.0590	8.7913	8.6687	10.3963	8.8010	12.2440
9.6117	8.2837	8.0090	10.4697	8.2620	5.3760	6.2097	3.9307	10.1423
-0.4830	2.1290	1.1660	3.5083	1.7970	4.8783	-1.4187	-5.0387	-1.1783
27.0820	24.4953	27.4693	25.0397	25.2297	26.0713	21.7847	25.3377	25.1713
8.7263	4.5613	10.4010	11.2227	11.4807	6.9533	6.9577	8.0857	5.2077
1.7863	-1.0917	1.5710	3.9500	0.0493	-3.1457	1.9567	-2.4587	-5.4200
10.4100	14.7197	12.0483	17.1307	14.9553	13.8383	11.8440	15.8583	17.5653
10.0657	7.5930	9.0487	15.7460	11.2543	9.3287	3.9793	7.4480	6.4410
2.6343	-1.9653	3.6487	3.4993	1.7213	-2.0900	1.6757	-0.1553	-2.4087
12.3970	13.3017	11.2563	11.5087	11.5560	11.3573	12.8797	13.0600	14.5007
8.2700	17.1530	18.0143	16.1487	17.7777	14.0100	14.8307	12.8770	13.4280
16.7823	17.8110	14.8153	14.2570	17.5413	14.6477	15.8627	19.4373	20.9543
11.1940	12.7033	11.0727	11.7887	10.5827	11.9893	8.8383	12.4113	11.9023
11.4110	8.9813	13.3030	13.5543	9.7790	8.3603	6.6690	7.9183	9.3203
15.8533	14.2730	16.9643	18.7750	15.1693	14.6507	12.1283	15.5643	17.4950
22.0883	19.5833	20.3377	25.5797	19.9673	18.9270	17.4103	18.6667	18.6003
17.8570	12.9213	15.0760	14.0553	16.0260	14.1230	13.2537	15.4820	8.6477
12.7040	15.5097	14.1693	16.2667	15.9877	11.6577	13.4187	12.8063	15.3417
5.2987	4.0007	7.3900	9.0427	4.1613	3.2423	6.0553	-1.8223	4.0990
17.2347	16.2130	15.6280	17.7560	13.0943	18.1047	17.0983	13.0807	17.3507
13.0980	9.7420	10.3227	14.3873	13.9557	8.4013	13.0890	12.0240	5.6017

12.9277	13.3907	13.8970	13.8657	11.2167	9.0840	8.2093	12.3183	6.5503
13.1540	14.0440	14.0643	12.2930	11.0003	12.4690	12.8663	12.0743	9.0207
6.4420	4.9097	5.9957	8.6850	6.7900	4.3887	3.7613	-0.3257	3.3800
11.0700	13.1077	12.5230	13.2747	11.3677	10.7587	15.1407	9.2187	11.3510
16.6843	16.9610	16.0443	14.2030	15.2397	15.6900	13.0530	14.0093	20.4080
17.2133	18.4657	14.9260	13.5900	15.4160	19.2693	15.4213	16.1867	16.1407
6.6337	1.9070	2.4827	6.0810	4.0417	-0.3310	1.0373	-2.2503	1.0673
4.2033	4.0400	4.4240	6.2383	4.6013	4.9053	4.1247	2.9583	-0.0753
23.4113	23.7813	25.1073	25.8013	23.8070	22.2643	23.4733	22.4100	18.9200
23.1823	22.2450	21.4820	23.0310	21.6353	21.7607	22.6190	25.1960	25.7013
9.7717	9.5970	10.6803	12.9920	9.1297	4.3260	8.3653	5.3620	2.9093
10.7330	6.6027	9.6073	8.2320	8.6493	9.6873	5.9457	6.0427	9.3297
14.4123	12.2883	13.5763	12.8990	10.9317	10.8510	6.5760	12.0263	13.9853
8.9363	7.4540	14.9467	12.6023	8.6610	9.3720	4.5670	9.0587	5.9457
4.6433	9.2960	4.4277	10.6713	5.1410	10.0047	5.2203	1.4510	6.8750
20.1217	18.2363	17.4737	19.2290	17.0257	17.9970	16.9867	16.6667	15.3397
11.3010	10.6220	10.8033	9.3367	10.1747	9.6890	9.9603	9.7693	9.8843
10.2120	12.0307	12.8183	11.6200	10.6307	11.1773	7.8397	8.4607	13.5610
23.9453	21.7687	22.0827	24.0327	21.8600	22.6310	20.6637	22.3403	22.7367
6.5457	7.2643	8.7007	6.9933	8.2873	6.3223	6.9183	6.0757	10.3213
24.0117	23.2377	25.8603	26.5003	23.6170	21.7730	19.9643	22.1133	24.7747
6.7063	6.9677	4.9547	6.8753	7.4533	7.2620	5.4900	8.9140	6.1883
7.5683	7.5493	8.1340	9.1280	9.4053	9.3863	9.0190	8.7987	9.1797
-2.6707	-3.9703	0.0220	3.1903	1.5140	-8.7967	-0.4947	-6.4267	-6.7457
17.3483	14.0977	12.6840	12.5620	13.3497	13.2520	16.0313	15.6730	14.0663
12.7947	13.1717	12.6863	12.2580	10.8333	11.6747	12.4043	16.4103	14.7180
7.9900	5.3957	6.6667	8.5427	7.9913	6.5613	5.9030	3.8630	7.6837
8.0357	5.9817	7.0773	13.3897	10.0077	7.3857	8.8933	6.4497	6.1960
12.6090	9.4793	9.6297	13.2160	8.5377	7.8473	7.6910	7.9823	6.5007
0.4930	4.8377	3.4100	4.1397	1.0580	4.3357	1.1457	-0.0460	3.2747
10.2400	10.7643	9.2240	13.2533	10.3227	11.3250	8.8490	6.6033	5.9130
3.0047	2.1853	1.9613	4.0953	4.7763	3.3653	2.5457	2.4317	2.6307
8.0590	10.3397	9.0723	9.9290	8.5387	10.5850	7.4910	8.5343	9.2457

18.9520	18.8440	19.6330	16.9750	18.6913	19.4697	20.5023	21.0017	22.5223
14.7400	13.4463	12.5257	13.6883	13.5293	13.8697	11.5697	11.8750	11.9347
-2.2007	-2.6693	-3.5023	0.1310	-1.0740	-3.5330	-5.8767	-7.9153	-7.0853
3.9433	4.0187	4.4860	5.5920	5.6417	3.3727	-0.2033	0.6657	1.6957
-3.9390	-2.0147	2.8887	2.5507	-2.3690	0.0863	-1.7493	-1.9830	-5.7533
19.2420	19.6543	17.8910	18.4330	18.6900	19.8603	16.4190	16.3720	17.1303
10.0253	10.8877	12.5510	14.1083	9.1963	7.5337	10.7713	8.4870	8.7130
5.1333	6.9693	4.2260	6.5737	4.3260	6.2360	4.8107	5.6540	6.1877
21.1737	20.0000	16.9327	20.1693	22.2347	19.8600	18.3277	17.7273	24.4810
25.1740	22.4973	19.3597	20.1357	20.9820	18.4083	25.1593	24.0540	23.2767
22.1667	21.5437	21.1837	19.9800	18.7430	21.6017	21.3407	22.0720	15.4620
27.5740	23.3277	25.9557	25.0340	24.3840	26.8967	26.1347	25.4223	20.7957
10.1967	11.5093	12.6897	14.4717	9.9230	11.8403	9.9410	6.5357	6.6927
22.3390	22.2000	22.9977	22.5140	19.7640	20.2560	16.9490	18.2567	18.9053
15.9830	17.4887	15.6923	16.7893	17.4517	19.9233	19.6547	16.4387	19.8403
16.2993	17.7710	16.8190	17.8917	16.0397	16.6963	14.4797	14.1287	13.8457
6.0733	5.3770	8.9023	10.7873	6.1423	2.2833	3.9207	1.2490	2.0323
8.8707	9.2953	10.9480	10.9553	9.5280	9.2840	7.7580	3.9943	9.0570
11.4907	8.6100	12.0700	9.3747	10.6187	8.1633	9.0347	8.0097	7.6303
11.1863	11.0607	9.4890	13.3887	13.7517	9.8073	5.6177	9.6220	13.0393
16.5047	13.2337	17.6403	16.9627	17.5733	13.7347	16.2490	14.9883	14.6540
11.5337	9.5033	11.3587	10.6013	12.4203	11.1617	10.5687	10.4400	10.2430
10.1947	10.1973	10.7890	11.4170	8.2260	12.2420	10.7587	4.7107	6.6647
10.2750	10.1770	11.1970	10.5130	10.2670	10.4667	10.9930	9.2900	13.1517
22.2117	19.3353	21.8050	22.2580	23.1317	22.9693	19.9617	19.7077	23.1133
13.2957	11.4607	10.6153	12.3783	14.0887	9.9740	9.6740	10.0793	10.1560
8.6847	4.1917	5.5117	7.2520	5.4393	3.8293	1.4417	1.8350	1.4957
15.2223	13.7260	13.8810	15.2553	14.0820	14.7790	15.3327	15.5057	14.9533
19.2707	15.9367	17.9583	13.9627	16.1220	15.8110	17.5813	18.2400	18.6937
8.4563	11.2240	11.5837	11.7350	14.7123	11.9193	10.1430	12.1160	10.8817
15.3393	15.9813	16.7680	16.4343	15.7127	15.6277	14.1147	18.8830	16.6767

783.17nm	786.04nm	789.09nm	792.12nm	795.16nm	798.01nm	801.03nm	REDchroma	LIGTHness
9.2037	18.5670	13.1867	14.9170	15.0230	10.6000	11.5340	0.1650	3506.1517
13.3523	13.0550	15.8077	15.0880	13.3330	14.6283	11.9560	0.2118	3139.2883
9.7220	6.7967	6.4090	9.7453	6.5077	15.4470	9.2857	0.2440	2094.6473
17.9060	15.9617	9.7633	13.6067	14.4530	15.3183	17.6940	0.1945	4011.3297
31.7627	28.8037	28.1937	27.8137	32.5800	32.2093	30.9483	0.2325	3840.9813
12.0447	11.1113	11.1647	9.1547	9.0920	12.2270	9.0383	0.1975	1525.4643
2.3017	0.1753	1.6753	-6.5900	-0.8403	0.8367	-9.7423	0.2214	2368.0913
8.2997	10.3997	12.3940	11.3887	8.5543	15.9290	13.5903	0.1886	2940.7107
7.9350	13.4960	12.4130	9.2677	10.1677	9.9353	10.1643	0.1872	1817.6153
8.2990	6.5243	9.1657	12.0303	6.9460	12.6463	11.4907	0.2013	1631.6763
7.7687	4.9703	5.5897	8.7017	4.8943	6.5193	7.9370	0.1832	2165.7607
-1.1563	0.2640	-1.2793	-1.3313	-4.4857	0.1600	-4.6470	0.1828	1220.1580
25.8413	20.7410	24.2243	25.3127	26.9067	21.4837	26.1977	0.1964	4493.3693
6.2407	9.0277	3.8823	5.5597	7.6923	12.8937	9.6367	0.1502	2578.5853
-3.9217	-12.8483	-1.3810	-4.7137	-6.0560	-6.3577	-9.6017	0.1586	1222.0420
15.8573	11.5730	13.5200	17.0350	12.0160	18.3660	18.1410	0.1948	3350.1793
3.8350	-0.8150	1.5507	-0.5133	2.7290	1.9487	-6.9073	0.1893	3629.3120
-1.5343	-1.0950	-3.2893	-4.1650	-3.6297	-5.5333	-2.1620	0.2001	1246.9390
14.4583	11.8413	13.8017	11.9983	12.9500	8.9337	8.8983	0.2002	1939.4890
16.0767	11.4893	14.4460	12.3293	18.2763	12.1317	8.6450	0.2068	3444.9040
18.2107	20.2157	13.8367	20.2327	21.0473	18.4723	19.3157	0.2069	2852.5670
14.0327	15.2740	10.1093	11.8087	10.6973	11.9210	7.9060	0.2043	2058.9770
8.0843	8.2317	8.1863	3.9007	3.9100	9.5777	8.0737	0.1837	3164.6047
16.4157	13.1090	17.7057	11.7777	17.0630	12.8100	14.5703	0.2269	2883.3200
15.0003	17.3007	14.7713	16.3807	9.5050	21.8487	23.5810	0.2324	4555.4047
12.2187	13.0820	11.9310	9.9490	11.3143	6.6007	8.2933	0.2019	3266.8113
13.2163	9.9963	16.5573	16.9027	9.5983	8.1617	18.5570	0.2163	3294.4317
3.3093	1.3950	0.3567	1.6107	1.3843	-0.6793	2.6243	0.1975	1685.9677
10.0117	14.6857	8.2230	16.6143	10.5557	15.4857	19.2857	0.2792	2528.6097
5.5620	11.7897	11.9340	14.2490	5.7887	6.3473	6.6723	0.1781	3617.5470

3.7503	7.5297	13.2983	13.0683	3.4477	7.4750	9.8320	0.2330	2188.1593
11.5277	13.5910	14.3920	10.4270	13.1757	9.2377	7.6260	0.2444	1812.0430
1.5153	5.7637	0.8287	5.4347	1.2057	2.3390	0.6143	0.2501	1280.9703
9.8770	10.7430	8.6500	8.6123	12.4967	7.5867	9.7707	0.2250	2270.8457
12.8267	14.0690	16.5433	16.0740	15.5280	18.8630	11.8277	0.2437	2408.5413
15.5497	17.9360	16.1270	17.6803	16.9727	14.1277	16.4763	0.2368	1928.2020
-1.8017	2.0993	3.4620	-2.7823	-4.2567	-3.5610	-8.9223	0.2843	785.8923
1.5627	2.7470	6.7160	0.4367	-0.4047	3.8163	-1.4960	0.2299	997.1593
20.0430	18.2603	24.2593	20.3993	19.3477	17.7383	19.4607	0.1744	5423.4883
24.1613	20.9853	15.6117	24.7410	26.0187	25.8600	26.4750	0.2108	3904.2110
2.0843	2.5100	8.0793	2.8390	-1.4933	3.8137	2.8743	0.2148	1998.0863
4.1833	6.8500	7.1137	2.0910	2.2520	5.5647	9.0233	0.2114	1406.5220
11.8370	15.3683	6.0777	11.8390	12.2503	10.4437	13.7767	0.3024	1392.0387
2.3420	3.2867	11.2787	6.2520	8.4473	3.3717	-3.9070	0.2798	1851.5337
2.2527	6.7890	2.4490	1.4593	3.9567	3.1193	-0.4910	0.2061	1599.9547
11.1723	11.7167	16.1160	19.8463	17.0983	13.0020	17.5573	0.2405	2708.5937
4.0113	11.4943	11.5517	8.9973	8.6717	3.4447	10.6107	0.2619	1495.7397
7.2063	11.8733	10.1240	10.5747	8.2523	10.2793	11.7397	0.1709	3315.6400
19.5150	21.6057	22.0897	19.5320	21.7743	19.2243	22.6710	0.1929	4492.7797
2.8847	5.5507	5.8887	2.5813	6.0117	4.1410	3.8777	0.1967	1839.7563
21.3643	20.5873	20.3103	23.1653	23.4387	23.3343	18.8597	0.1935	5116.5880
1.2333	3.9757	7.6150	2.7937	7.1610	6.8773	3.7037	0.2019	1473.9700
8.4813	9.9353	9.0743	7.9463	9.7587	10.5507	8.5560	0.2719	1199.4897
-4.7343	-2.3333	-3.5817	-6.4713	-10.3817	-12.6023	-11.7300	0.1330	1930.6970
13.5447	20.3183	15.8283	15.4173	13.4287	16.1263	16.8383	0.2593	1958.8010
14.8507	16.2333	15.2497	13.8273	14.7630	13.0657	14.9320	0.2264	1837.6477
1.0147	8.7303	8.4703	3.5007	3.6927	6.8667	6.4923	0.2392	1711.8650
0.5447	1.9240	4.5050	4.3527	6.1107	3.5037	5.9903	0.1734	2553.3040
6.8197	9.3037	10.1383	7.8080	10.8207	4.7927	11.3083	0.1576	3492.4173
4.0887	1.8523	-1.6087	2.4960	2.2037	1.7320	4.6743	0.1661	1200.9130
10.2317	6.7910	6.7667	1.3993	7.6917	7.6453	4.4060	0.2072	2335.5753
2.7067	0.3697	3.0683	-1.4623	2.5867	2.4293	2.0310	0.2993	499.6460
9.5390	10.0807	9.7190	7.8627	9.5913	6.0897	10.7037	0.2139	1494.0320

18.4093	22.0247	20.8583	22.7970	17.4727	22.6867	17.7813	0.2343	2454.3077
11.0720	12.1233	13.4943	11.1367	7.2657	14.5070	12.3827	0.2448	2289.4973
-9.5353	-7.7233	-6.9840	-7.9890	-11.0470	-13.6703	-15.0493	0.1380	1117.9803
-0.9403	2.5217	-0.1817	-4.5133	-4.7117	-3.0500	0.9207	0.1758	1483.9577
-2.1797	-3.6180	-3.2460	-4.8010	-8.3453	-8.3033	-10.5527	0.1562	1234.3017
20.6927	20.8423	15.3833	15.0720	20.1297	15.0630	15.8190	0.1976	3379.6797
7.9727	7.0227	7.9387	10.1837	7.0727	7.1743	10.1160	0.1630	3167.0907
5.2683	3.7920	6.0290	-0.4480	3.4307	7.4607	6.1153	0.1647	1373.6907
19.9980	17.4163	21.1900	21.0370	16.2887	18.1150	15.8963	0.2671	3275.4923
21.0557	22.1370	21.0300	21.5330	23.9097	16.7750	19.2143	0.3039	2498.8867
21.3913	18.3777	16.8763	22.0977	21.4833	16.6207	18.1157	0.1783	4962.9397
25.7063	23.6327	22.2923	29.9743	23.2667	25.5183	24.0163	0.1983	4674.9280
7.1310	7.4373	8.2283	11.2257	7.8213	5.1877	5.6050	0.1783	3600.5963
20.1643	16.3890	21.5353	16.1890	18.0807	17.6043	19.2903	0.2048	4582.2220
15.4640	21.7673	18.2577	22.6140	20.5777	19.7650	19.7823	0.2621	2132.7647
12.1200	9.5313	11.8400	14.2717	13.5863	8.2857	13.6630	0.3017	2618.6273
1.0767	-4.6880	4.2180	-4.3513	-2.6143	-1.3107	-5.8773	0.2273	2118.0777
3.9773	8.2967	3.8313	2.2253	7.4593	2.0520	4.4923	0.2131	2170.6237
8.0350	9.0633	10.4423	8.9117	7.2513	8.1810	11.1870	0.2228	1668.9633
8.6873	3.9840	3.3403	7.7837	7.4263	9.6083	8.1537	0.2364	2567.2100
16.2113	16.3923	13.1257	14.1820	12.4987	12.2670	15.8593	0.2507	2375.2097
9.4793	9.0457	10.0937	8.7440	10.1383	9.4280	11.8257	0.3037	1320.1367
5.3310	6.5597	7.6193	2.5210	2.5613	3.6873	4.2870	0.2518	1971.6767
11.9677	11.8307	7.5360	9.3833	8.4163	12.5023	12.1117	0.2399	1497.4340
20.6940	18.5270	23.1753	19.1640	21.0650	18.7747	20.4337	0.2477	3475.4767
9.6953	9.9527	8.1677	5.1867	11.3397	4.5147	6.8670	0.2091	2307.1823
0.2837	3.1287	2.4777	1.7353	0.6757	1.5430	1.6727	0.2000	1507.0843
13.6210	14.1320	14.0510	11.4270	15.3493	11.7200	16.8957	0.2439	2110.9570
17.3693	16.9113	17.0690	19.7677	17.9557	14.7483	16.2320	0.2456	2117.8100
13.6513	11.9307	12.5420	10.7220	9.8060	8.0697	9.0137	0.2377	2116.2307
16.8683	14.8243	14.3510	15.4050	13.5890	14.2870	12.9017	0.2398	2714.5570

MAX(500-700)	HUE
26.7700	528
22.4177	615
17.2843	678
30.1540	609
27.5487	675
9.9820	675
22.8703	627
22.8357	567
12.6233	672
10.9660	675
18.2423	531
12.5970	531
30.2593	558
23.0270	528
11.8463	528
24.8043	579
34.7913	528
11.2840	543
13.3517	696
28.4643	606
19.4523	618
14.5727	669
24.9197	546
21.4030	669
41.9673	603
26.0753	603
28.3100	597
13.6567	594
23.1457	615
30.2910	543

16.9240	618
14.2443	645
12.6713	603
17.0740	600
18.7133	600
15.0517	669
9.2547	618
8.9297	696
42.6847	534
26.8370	615
15.5970	636
10.5703	615
13.8000	690
20.2190	597
12.4663	603
21.7227	699
12.8383	690
24.6843	537
31.6250	501
14.7487	546
39.4310	534
10.8327	555
10.2230	678
22.5360	528
15.6527	666
13.2017	684
15.5073	600
20.2710	573
27.6210	528
9.5837	516
18.9630	540
5.2313	669
10.6510	612

17.7060	693
18.4203	666
14.3847	528
11.6543	558
16.5347	528
23.5663	555
24.7957	540
10.1407	507
29.0223	636
22.0023	675
35.5790	588
31.2420	630
29.5197	573
36.6183	597
16.9177	675
26.0400	636
21.5640	606
16.9903	543
12.6850	648
23.1033	606
18.6770	687
12.2157	669
17.5047	660
11.5857	687
27.7170	636
17.7313	597
12.0533	603
16.1500	654
15.9473	645
17.2000	621
21.0660	624
